# Leveraging Adventive and Endemic Parasitoids Against Polyphagous Agromyzid Leafminers in Australia

**DOI:** 10.3390/insects16090968

**Published:** 2025-09-16

**Authors:** Peter M. Ridland, Elia I. Pirtle, Paul A. Umina, Ary A. Hoffmann

**Affiliations:** 1 Pest & Environmental Adaptation Research Group, School of BioSciences, Bio21 Institute, The University of Melbourne, Parkville, VIC 3010, Australia; pumina@unimelb.edu.au; 2 Cesar Australia, 95 Albert St., Brunswick, VIC 3056, Australia; eliapirtle@gmail.com

**Keywords:** *Liriomyza*, biocontrol, Agromyzidae, mass-rearing, augmentative control

## Abstract

The recent incursions of three agromyzid leafminers (*Liriomyza huidobrensis*, *L. sativae*, and *L. trifolii*) into Australia pose a significant threat to horticultural production. Outbreaks of these polyphagous pests are consistently associated with the disruption of their parasitoids caused by the widespread use of non-selective insecticides and to increasing pesticide resistance in these agromyzids. An analysis of the international literature for records of parasitoids associated with *L. huidobrensis*, *L. sativae*, and *L. trifolii* revealed that they are attacked by a similar profile of parasitoid families: Eulophidae (63% of records), Braconidae (19%), Pteromalidae (9%), and Figitidae (8%). *Diglyphus isaea*, *Neochrysocharis formosa* and *Hemiptarsenus varicornis* (all already recorded in Australia) were the most commonly reported parasitoids worldwide. These species, along with Australia’s broader parasitoid fauna, will play an important role in managing invasive *Liriomyza* species, provided insecticide use does not disrupt their populations. However, key knowledge gaps remain, especially regarding the taxonomy of local parasitoids, their potential use in conservation biocontrol programs, and the local landscape features that support parasitoid populations. The use of various species in augmentative releases is considered, including constraints and opportunities related to strain selection and the potential use of thelytokous strains for mass-rearing.

## Simple Summary

The larvae of agromyzid leafmining flies (Diptera: Agromyzidae) feed between the epidermal layers of host plant leaves, creating characteristic feeding tunnels or ‘mines’. Three polyphagous agromyzid leafmining flies (*Liriomyza huidobrensis*, *L. sativae*, and *L. trifolii*) have recently arrived in Australia and threaten commercial vegetable and cut flower production. Overseas, outbreaks of these pests have been linked to the widespread use of non-selective insecticides, which disrupt biological control by parasitoid wasps that attack larvae within the leaf mines. An analysis of international records of parasitoids associated with the three *Liriomyza* species showed that *Diglyphus isaea*, *Neochrysocharis formosa*, and *Hemiptarsenus varicornis* (all already present in Australia) are the most commonly recorded worldwide. Protecting open-field production will require fostering parasitoid populations as part of integrated pest management programs. The high cost of mass-reared parasitoids will likely restrict augmentative releases to greenhouse production, but costs could be reduced if female-only strains can be identified or selected. Biological control should be a key tool for Australian growers to manage *Liriomyza* pests when minimizing insecticide use.

## 1. Introduction

The leafminers *Liriomyza huidobrensis* (Blanchard), *Liriomyza sativae* Blanchard and *Liriomyza trifolii* (Burgess) (Diptera: Agromyzidae) are recognized globally as significant pests of vegetables and ornamental flowers [1]. The global spread of these polyphagous *Liriomyza* species has occurred largely through international trade of flowers and vegetables [2,3,4]. *Liriomyza huidobrensis* and *L. trifolii* present greater control challenges than *L. sativae* due to their higher levels of resistance to insecticides [4,5,6,7,8,9,10]. In the USA, *L. trifolii* has largely displaced *L. sativae* in horticultural regions [8], with a similar trend emerging in southern China [8,11,12]. *Liriomyza trifolii* was first recorded in Kenya in 1976 on chrysanthemum cuttings imported from Florida [3] and later found in South Africa in 1983 [13]. Infested chrysanthemum cuttings from Kenya and Malta are believed to be the source of *L. trifolii* for its introduction to Europe in the early 1980s [3]. However, *L. huidobrensis* has emerged as the predominant agromyzid across all elevations in Kenya [14] and is now recognized as the most widespread potato pest in South Africa [15,16]. In Europe, both *L. huidobrensis* and *L. trifolii* are major pests of greenhouse-grown vegetables and flowers.

In Australia, all three polyphagous *Liriomyza* species have now been detected. *Liriomyza sativae* was first recorded on the Australian mainland at Seisia, near the tip of Cape York, in 2015 [17], following its identification in the Torres Strait in 2008 [18,19]. Ten years on, it remains localized at Seisia. *Liriomyza huidobrensis* was first confirmed from field-grown vegetables in Sydney in October 2020, and in southern Queensland in November 2020 [20,21]. *Liriomyza trifolii* was first recorded in the Kununurra region in far northern Western Australia in March 2021 and on Thursday Island, Queensland in May 2021. Additional specimens have since been found in the Northern Peninsula Area of Cape York Peninsula and the Northern Territory [22]. In 2025, *L. trifolii* was detected for the first time in the Lockyer Valley horticultural region in SE Queensland, 2700 km southeast by road from the known initial outbreak in the Cape York Peninsula [23]. COI haplotypes have now been established for Australian populations of all three species, along with their *Wolbachia* infection status [24,25].

With the recent incursions of *L. huidobrensis* and *L. trifolii* in Australia, it is essential to reassess the parasitoids in Australia that could target these species, as invading *Liriomyza* populations are often rapidly exploited by local parasitoids [26]. An extensive literature survey [27] highlighted a substantial local endemic and adventive parasitoid community in Australia, primarily consisting of species in the Eulophidae, Pteromalidae and Braconidae, known to attack agromyzids. Surveys of *Liriomyza brassicae* (Riley), *Liriomyza chenopodii* (Watt), *Phytomyza plantaginis* Goreau and *Phytomyza syngenesiae* (Hardy) and their associated parasitoids have been conducted in eastern Australia [24,28,29,30,31,32,33,34]. Three cosmopolitan species, *Diglyphus isaea* (Walker), *Hemiptarsenus varicornis* (Girault) and *Neochrysocharis formosa* (Westwood), were the most abundant, along with four Australian endemic species, *Zagrammosoma latilineatum* Ubaidillah, *Trigonogastrella parasitica* Girault, *Closterocerus mirabilis* Edwards & La Salle and *Opius cinerariae* Fischer, about which little is known regarding their biology and ecology.

This review complements the recent synthesis of parasitoids of *L. sativae* [27] and examines global records of parasitoid wasps reared from *L. huidobrensis* and *L. trifolii* to assess whether Australia’s existing assemblage of agromyzid parasitoids may be adequate for control of these newly invasive species. We then discuss the challenges and opportunities for augmentative and conservation biological control of *Liriomyza* spp. in Australia.

Any compilation of species names derived from scientific literature inherently contains some uncertainty regarding the accuracy of species identifications, and the completeness of any list will depend on the extent of scientific engagement with the issue [35,36,37].

During the 1950s and 1960s, there were widespread difficulties in correctly identifying *Liriomyza* spp. [38,39]. Accurate identification of their parasitoids is even more difficult, with very few taxonomists available to confirm identifications, even for species that have been formally described. Consequently, reared parasitoids are sometimes only identified to genus level, and some questionable identifications have been published—errors that can be perpetuated in subsequent parasitoid lists [36]. Additionally, there is growing evidence of cryptic species among parasitoids, as in the case of *N. formosa* [40,41,42] and *D. isaea* [43,44], which may represent unresolved species complexes. Molecular diagnosis is then required to verify records in different regions, and voucher specimens should be deposited in appropriate insect collections to be databased and available for future reference [45,46].

## 2. Parasitoids Reared from *Liriomyza* spp. Worldwide

For this review, we returned to the primary literature sources but used some previous lists as guides. Since 1987, several lists of parasitoids reared from polyphagous *Liriomyza* spp. have been published (Table 1). For *L. trifolii*, all data were retrieved from primary sources (Table S1). For *L. huidobrensis*, we used data from Weintraub et al. [4] as a foundation, with Supplementary Data added where found (Table S2). Data for *L. sativae* was based on Ridland et al. [27], again with Supplementary Data added where found (Table S3). Host and location records for parasitoids of each *Liriomyza* species were combined to prevent overestimating the importance of a particular species, especially in countries with disproportionate research effort (e.g., Japan, Turkey, and China). For example, we listed 10 records of *D. isaea* attacking *L. trifolii* in Turkey; these were combined to give a single location record of *D. isaea* attacking *L. trifolii* in Turkey. Parasitoids reared from mixed collections of *Liriomyza* spp. were excluded. Data collection concluded in October 2020.

The parasitoid assemblages for *L. huidobrensis*, *L. sativae,* and *L. trifolii* are very similar in family and sub-family composition (Table 2). Most species (63%) belong to the family Eulophidae, with representation equally split between the Eulophinae and the Entedoninae subfamilies. The Braconidae is the next most abundant family (19%), followed by Pteromalidae (9%) and the Eucoilinae subfamily of the Figitidae (8%).

Parasitoids of agromyzids are typically considered generalists, attacking a range of agromyzid species [26,27]. The degree of overlap in parasitoid species among the three *Liriomyza* species for the major subfamilies ranged from 46% (Eulophinae) to 31% (Opiinae) (Table 2). About one third of parasitoid species have been recorded from all three *Liriomyza* host species, while a further 13% have been recorded from two species (Table 3). Seventy-nine species have been recorded from a single host species, largely reflecting records of native parasitoids in the region of origin of a given *Liriomyza* species.

As the international movement of all three *Liriomyza* spp. is a relatively new phenomenon, endemic parasitoid species often attack the invasive agromyzids [26,51], increasing the level of species overlap. Adventive parasitoids arriving in a country with other invasive agromyzids will also enhance the level of biological control when a new agromyzid species establishes in a country. The unintentional introduction of parasitoids with their invasive hosts is becoming increasingly common due to the increasing volume of global trade [52]. For example, two Asian parasitoids of *Drosophila suzukii* (Matsumura) (Diptera: Drosophilidae), *Leptopilina japonica* Novković & Kimura (Hymenoptera: Figitidae) and *Ganaspis kimorum* Buffington (Hymenoptera: Figitidae), under evaluation for possible release in North America, have now established adventitiously in British Columbia [53]. Furthermore, *L. japonica* has now been recorded parasitizing *D. suzukii* in cherries in Italy [54], which is the first record from Europe. A similar case was observed with the plane leafminer, *Phyllonorycter platani* (Staudinger) (Lepidoptera: Gracillariidae). When this insect arrived in the British Isles, it was accompanied by its specialized eulophid parasitoid, *Minotetrastichus platanellus* (Mercet). This species now causes a higher percentage of parasitism on *P. platani* than the specialist parasitoids that target native leafmining hosts [55]. However, the parasitoid communities that develop on introduced hosts are generally less diverse and tend to include more generalist species compared to those on native hosts [56]. These groups need time to establish, as native parasitoids must adapt to new host species. Early interactions may involve occasional attacks from species that are not well-suited to the invasive host, which continue only because the invader coexists with native hosts that support the parasitoid populations [56]. This suggests that many groups of parasitoids associated with newly introduced hosts, like *Liriomyza* species, may not have yet adapted effectively for sustained use of these hosts.

The family and sub-family composition of parasitoid assemblages summarized in Table 2 is reflected in Table 4. Here four genera of Eulophidae [*Diglyphus* (Eulophinae), *Chrysocharis* (Entedoninae), *Neochrysocharis* (Entedoninae) and *Hemiptarsenus* (Eulophinae)], one genus of Braconidae [*Opius* (Opiinae)] and one genus of Pteromalidae [*Halticoptera* (Miscogasterinae)] are the six most commonly recorded genera parasitizing the three *Liriomyza* species worldwide, together accounting for 59% of records.

The four most common parasitoid species recorded parasitizing *L. huidobrensis*, *L. sativae* and *L. trifolii* worldwide (Table 5) are *D. isaea*, *N. formosa*, *H. varicornis* and *Chrysocharis pentheus* (Walker).

In Australia, *D. isaea* and *N. formosa* are recent adventive species recorded parasitizing *Liriomyza* spp. in south-eastern Australia [30,32,33]. *Hemiptarsenus varicornis* is a cosmopolitan species widely distributed in Australia and other parts of the world [33,58]. *Chrysocharis pentheus* is a common larval-pupal endoparasitoid of *Liriomyza* spp. in Asia [59,60,61] but has not yet been recorded in Australia. *Chrysocharis pubicornis* (Zetterstedt) is primarily a pupal parasitoid of agromyzids and is the only representative of the genus in Australia [58] and is not considered an important parasitoid of *Liriomyza* spp. [29,62]. While several *Opius* spp. parasitize agromyzids in Australia [63], additional research is needed to elucidate the taxonomy and biology of the opiine braconid complex that target agromyzids in the region.

The local parasitoid assemblage lacks species from the Miscogastrinae, Alysiinae and Eucoilinae taxa. The miscogastrine genus, *Halticoptera*, is the most common pteromalid genus attacking polyphagous *Liriomyza* spp. in North and South America [64]. Only two species from the Miscogastrinae are described in Australia: the cosmopolitan *Ammeia pulchella* Delucchi and the indigenous *Rhicnocoelia incisa* Boucček. Two additional Australian species, *Lamprotatus damia* (Walker) and *Lamprotatus nicon* (Walker) are considered to be *incertae sedis* as both type species are missing [58]. However, at least one *Halticoptera* sp. has been recorded in Australia and a second species in Papua New Guinea. These are likely introduced species, but no Australo-Papuan species of *Halticoptera* have yet been formally described [58].

The adventive alysiine, *Dacnusa areolaris* (Nees), which exclusively attacks *Phytomyza syngenesiae*, *Phytomyza asteris* Hendel and *Phytomyza nigra* Meigen [65], remains the only *Dacnusa* species recorded in Australia and New Zealand [66]. The endemic *O. cinerariae* could be evaluated as a mass-rearing alternative to *Dacnusa sibirica* Telenga (Hymenoptera: Braconidae: Alysiinae) to complement *D. isaea* releases in protected cropping. Until recently, there were no confirmed records of eucoilines parasitizing agromyzids in Australia [27,67]. However, in 2021, an unidentified eucoiline species was reared from puparia of *L. huidobrensis* infesting celery crops in Queensland (PM Ridland unpublished data).

Further collections in Queensland may reveal more species in the Eucoilinae and Miscogastrinae, as a few specimens of the eucoiline, *Gronotoma domestica* Girault, and the pteromalid, *Halticoptera* sp., have been recorded in that state without host records [58].

The braconid *Opius scabriventris* Nixon, the eulophid *Chrysocharis flacilla* (Walker), and the pteromalids *Halticoptera arduine* (Walker) and *Halticoptera helioponi* De Santis, are the most abundant species parasitizing *L. huidobrensis* in Argentina [68,69] and Peru [70]. Note that the sub-genus *Phaedrotoma* was split from *Opius* in 1997 [71] but has recently been synonymized with *Opius* [72]. The first three species have recently been introduced into Kenya for the biological control of *L. huidobrensis* [73,74,75,76], with *O. scabriventris* appearing to have established successfully [77]. The long-term outcomes of these releases will provide insights into the utility of importing and releasing exotic parasitoids in a classical biological control program targeting *L. huidobrensis*.

Before investing in the time-consuming and costly importation of additional exotic parasitoid species [78], the impact of endemic and adventive parasitoids in Australia on invasive *Liriomyza* spp. needs thorough evaluation in the field. With increased surveying efforts across Australia, together with the recent rise in *L. huidobrensis* and *L. trifolii* populations in horticultural areas, the number of identified parasitoid species is likely to increase markedly.

However, detecting candidate parasitoids is only the initial step in developing an effective biological control program for polyphagous *Liriomyza* species. Key challenges include ensuring that parasitoids are not adversely affected by the overuse of inappropriate insecticides and identifying and managing the reservoirs of both parasitoids and non-target agromyzids [27]. The dispersal of agromyzids and their parasitoids across cropping regions, adjacent non-crop areas, and sequentially planted crops remain poorly understood [27]. In some cases, growers could use augmentoria constructed with fine mesh to allow parasitoids to emerge from mined plant foliage while preventing *Liriomyza* adults from escaping [27,79].

Generally, the longer a crop remains in the ground before harvest, the greater the control of *Liriomyza* spp. by parasitoids due to multiple generations of parasitoids. Rapid rotation crops such as spinach and leafy greens, which remain in the ground for only 4–6 weeks, are unlikely to benefit directly from parasitoids, since the crop will be harvested before the parasitoids can develop a second generation, and mined leaves will be harvested. However, high parasitism in other crops and non-crop plants should reduce the invasion pressure of *Liriomyza* flies on new crops. In intensive lettuce production areas in Arizona, growers rely heavily on insecticides to manage a range of pests, with pest monitoring and the choice of appropriate chemicals the primary pest management tools [80]. *Liriomyza trifolii* and *L. sativae* are now only occasional pests in Arizona, largely due to the use of selective insecticides targeting lepidopteran pests and thrips, such as spinetoram and diamides, that preserve the natural enemies of the leafminers [81].

## 3. Augmentative Releases Overseas

*Liriomyza bryoniae* emerged as a major pest of tomatoes in north-western European glasshouses in the mid-1970s, following the adoption of rockwool or nutrient film plant cultivation systems [82,83,84]. Previously, winter soil disinfestation effectively eliminated overwintering puparia, so leafminer infestations during the crop’s growth occurred only in spring or summer due to invading flies once the plants were well-established. When this management practice ceased, *L. bryoniae* emerging from the overwintering puparia could infest crops immediately. The arrival of *L. trifolii* in European glasshouses in the late 1970s exacerbated problems, as growers were unable to achieve adequate results with conventional insecticides due to resistance. In many crops, insecticide use was curtailed to facilitate biological control of greenhouse whitefly, *Trialeurodes vaporarium* (Westwood), by *Encarsia formosa* Gahan [85]. This decrease in insecticide pressure allowed two endemic species of braconid parasitoids, *Dacnusa sibirica* and *Opius pallipes* Wesmael (Hymenoptera: Braconidae: Opiinae), to overwinter in glasshouses alongside *L. bryoniae* puparia, which contributed to early season leafminer control [84]. Later in the season, the endemic eulophid ectoparasitoid, *Diglyphus isaea* (Walker), arrived in crops from surrounding areas and often controlled leafminer infestations when broad-spectrum insecticide applications were curtailed [86].

In the 1980s, numerous trials were undertaken in tomato glasshouses involving the natural colonization of crops by *L. bryoniae* and its parasitoids [82,87,88]. The highly variable colonization results prompted trials evaluating experimental releases of mass-reared parasitoids during the early stages of crop development. No systematic selection process was undertaken in Europe to identify the most appropriate species (or strains); instead, endemic species already present in glasshouses were mass reared. The imported eulophid endoparasitoid, *Chrysocharis oscinidis* Ashmead (previously known as *C. parksi*), was also assessed in several trials. Although *C. oscinidis* was an abundant parasitoid of *L. sativae* in field tomatoes in California [89], its control was inconsistent in trials [90,91,92,93], leading commercial rearing companies to concentrate on producing *D. isaea* sourced from local populations.

The main mass-reared parasitoid used for controlling *Liriomyza* spp. is *D. isaea,* supplemented by *D. sibirica* early in the season in northern Europe where *Liriomyza* spp. overwinter in glasshouses [94]. Both species are sold in over 20 countries and rank among the most important invertebrate biological control agents globally in augmentative biological control [95]. Other parasitoids mass-reared for *Liriomyza* spp. control include *N. formosa*, available in Asia since 1990, *Diglyphus begini* (Ashmead), reared in Latin America, and *O. pallipes*, which continues to be reared in small quantities in Europe [95,96,97].

In Brazil, detailed biological studies found that the endemic parasitoids, *Chrysocharis vonones* (Walker) and *O. scabriventris*, achieved total mean parasitism of *L. sativae* that was comparable to that achieved by *D. isaea* and *N. formosa* in Japan [98,99,100]. This demonstrated the great potential in assessing endemic parasitoids for augmentative control of *Liriomyza* species in invaded areas.

## 4. Augmentative Releases in Australia

To date, the only Australian work on augmentative releases was undertaken as part of a project on control of agromyzid leafminers in Australia and Indonesia [101]. *Hemiptarsenus varicornis* were released in the middle of 600 × 4000 mm beds, each planted with three rows of Chinese cabbage seedlings. Parasitoids were detected by placing sentinel Chinese cabbage leaves mined by *L. brassicae* larvae among the plants. Results showed a high level of parasitoid attack (2–3× greater than control plots), coupled with a similar difference in host mortality due to host feeding. In glasshouse releases of *H. varicornis* and *D. isaea*, both species persisted and impacted *L. brassicae* populations [102].

## 5. Assessing Candidates for Augmentative Control in Australia

Understanding the lifecycle of the polyphagous *Liriomyza* spp. is crucial when considering biological control agents in protected cropping. Female flies use their ovipositor to create fan-shaped feeding punctures in the epidermis, from which both female and male flies obtain nourishment through leaf exudate. The feeding punctures create a distinctive stippling on the leaf, indicating the presence of leafminers. In contrast, when the female deposits an egg beneath the leaf epidermis, the oviposition puncture is tubular [103,104].

After hatching, the first-instar larva starts to tunnel beneath the epidermis, creating the distinctive species-specific mine. The third instar exits the mine and drops to the ground to pupariate. When larval densities are high on celery, pupariation has also been seen on leaves, at the base of petioles, as well as on the soil surface [105].

Adult fly eclosion from the puparium begins around sunrise, which is also an important time for *Liriomyza* spp. management because of (i) mating and oviposition, and (ii) the exit of third-instar larva from mines [106,107,108]. The larval stage only occupies about 31% of the total immature developmental period, averaged over five temperatures from 10 °C to 35 °C. The egg stage accounts for 23% and the pupal stage 46% [109]. The first, second, and third larval instars of *L. sativae* occupy an average of 38%, 27% and 35%, respectively, of the total larval development time [110], and these periods are likely to be very similar in other *Liriomyza* species. Parasitoids attacking early third-instar larvae, therefore, have a limited window within the host lifecycle.

### 5.1. Idiobiont Eulophid Parasitoids

Most eulophid wasps (Hymenoptera: Eulophidae) parasitizing agromyzid leafminers are synovigenic idiobionts, meaning that they paralyze their host larva before oviposition or attack sessile stages such as puparia. They cause mortality by host parasitism, feeding, and stinging (where the larva is paralyzed but the female wasp does not oviposit or host feed) [111,112,113,114,115].

#### 5.1.1. Host Parasitism

Paralysis of the agromyzid larva determines the food resource available to parasitoids at the time of oviposition. To maximize resources for wasp larvae, eggs are usually laid in or near the third instar (the oldest larval stage). Ectoparasitoids such *D. isaea*, *H. varicornis*, *Cl. mirabilis* and *Z. latilineatum* deposit their eggs next to the paralyzed larvae. After hatching, parasitoid larvae feed on the paralyzed host, pupate beside the dead host, and subsequently emerge from the mine. In contrast, idiobiont endoparasitoids, such as *Neochrysocharis okazakii* Kamijo and *N. formosa*, also paralyze the host larva but oviposit inside the larva, enabling the parasitoid larvae to feed and pupate within the host before emerging [116].

#### 5.1.2. Host Feeding

Synovigenic idiobiont females emerge with very few eggs and need to feed on the hemolymph of hosts to sustain ovigenesis and egg maturation. Females locate and puncture smaller host larvae within mines. Wasps of both sexes then feed on hemolymph from these punctured larvae, which then die. Host feeding is closely associated with wasp fecundity and longevity [113,114,117].

#### 5.1.3. Host Stinging

Occasionally, females paralyze agromyzid larvae without ovipositing or feeding. This stinging kills the larvae and may serve to protect the wasp’s progeny by preventing other parasitoids from ovipositing in the same host [115].

In the case of idiobiont eulophids, adult wasps emerge from the mine, complicating commercial production, as producers must sell the wasps as adults with a very short shelf life, rather than as pupae. Typically, these wasps are sold in bottles containing either 250 or 500 individuals.

#### 5.1.4. Mass-Rearing Systems for Idiobiont Parasitoids

Systems for mass rearing of several idiobiont eulophid species have been documented, including for *D. begini* [118], *D. isaea* [119], *H. varicornis* [120,121,122], *N. formosa* [123,124], and *N. okazakii* [125]. However, apart from *D. isaea* and *D. sibirica*, no species has been commercialized on a large scale. Major commercial producers in Europe, Israel and North America have reared leafminer parasitoids for many years but their precise rearing protocols remain closely guarded intellectual property [126].

### 5.2. Koinobiont Parasitoids

Four braconid wasps parasitizing *Liriomyza* spp. overseas, namely *D. sibirica*, *O. dissitus*, *O. pallipes* and *O. scabriventris*, are common koinobiont larval-pupal endoparasitoids. These wasps do not paralyze the host larva at oviposition; the agromyzid larva continues mining until pupation. The parasitoid begins development only after the host larva pupates. The wasp will oviposit into any larval instar, as its development is delayed until host pupation. In most cases, the parasitoid can immunosuppress the host to prevent egg encapsulation by host haemocytes [127]. However, there have been several reports where indigenous parasitoids fail to successfully attack newly introduced agromyzids. In Europe, eggs of *O. pallipes*, but not those of *D. sibirica*, were encapsulated by *L. trifolii* [90]. Consequently, mass rearing and release in Europe focused on *D. sibirica* [83]. In contrast, the inability of *D. sibirica* to develop in *L. sativae* was considered important for the dominance of *L. sativae* over *L. trifolii* observed in Japan [128,129].

Mass-rearing techniques for *D. sibirica* and *O. pallipes* [130] using *L. bryoniae* on tomato have been developed, allowing one-week-old puparia to be stored at 5 °C. In the USA, mass rearing and release of *Opius dissitus* for control of *L. sativae* has been undertaken for many years at the EPCOT Center, Florida [131]. *Liriomyza sativae* is reared on bush lima beans (*Phaseolus lunatus* L. cv. Henderson) [132] and the process of collecting and counting *O. dissitus* pupae has been mechanized [133]. *Dacnusa sibirica* is released only in northern Europe during winter when temperatures are too cold for *D. isaea* to be effective [134].

*Banacuniculus* (=*Ganaspidium*) *utilis* (Beardsley) (Figitidae, Eucoilinae) is a koinobiont larval endoparasitoid. It was introduced into Hawaii from Texas and has become a dominant parasitoid of *Liriomyza* spp. [135,136]. It was subsequently introduced into Guam and Tonga, where it has apparently become the dominant parasitoid of *L. trifolii* [137].

## 6. Constraints Affecting Commercial Production and Deployment Success

Mass-rearing protocols published for several parasitoids of *Liriomyza* spp. [118,119,138,139,140] consist of three components: plant production, leafminer production and parasitoid production. Steps include:

- Maintaining a supply of host plants;
- Exposing host plants to leafminers for a short time to synchronize leafminer populations;
- Exposing leafminer-infested plants to parasitoids for a short time to synchronize parasitoid populations;
- Collecting leafminer puparia to maintain leafminer cultures;
- Maintaining parasitized leafminers (species-dependent);
- Collecting and packaging parasitoids.

Modelling of the rearing system of *D. begini* (closely related to *D. isaea)* has helped optimize production efforts [141,142].

Unfortunately, *Diglyphus* species are very expensive to rear. In the Philippines, the full cost of rearing 1000 *D. isaea* was estimated at USD 1132 [119]. Although established commercial insectaries have developed efficient large-scale rearing methods, an internet survey in June 2025 found the median retail unit cost in 2025 was USD 0.26 per wasp (assuming 1:1 sex ratio, the unit cost was USD 0.52 per female). Typically, 3–5 weekly releases are made in glasshouses per crop. Larger enterprises regularly ordering large quantities of wasps likely receive a lower unit cost per wasp. With typical release rates ranging from 0.25 to 1 wasp per m^2^ (equivalent to 2500–10,000 wasps per ha; see Table 6) and a cost of USD 0.13 per *D. isaea* wasp, each release can cost between USD 325 to USD 1300 per ha. Given these expenses, augmentative use of commercially reared *D. isaea* in open-field situations is unlikely to be cost-effective.

*Diglyphus isaea* and *D. sibirica* are the major parasitoid species of *Liriomyza* spp. reared worldwide, with one hundred thousand to millions of individuals sold per week [97]. Production of *D. begini* (Latin America) and *N. formosa* (Asia) is in the range of ten thousand to one hundred thousand individuals sold per week. In contrast, small numbers of *O. pallipes* (hundreds to a few thousand wasps sold per week) are distributed in Europe [97].

### 6.1. Storage

Production costs remain high because the adult wasps have a very short shelf life, and cold storage can affect a range of fitness-related traits [144]. For mass-reared *D. isaea*, cold storage has been investigated to extend the storage life and accommodate unpredictable demand. In laboratory trials, *D. isaea* wasps, stored at 4 °C for 120 days (and fed with honey and sucrose solution solidified in agar), had higher survival and fecundity than wasps stored at 10 °C [145]. In contrast, trials storing *D. isaea* at 15 °C, 20 °C and 26 °C for one month found optimal performance at 20 °C [146]. Notably, the sex ratio (% males) decreased to 29% at 26 °C, compared with 54% at 15 °C and 44% at 20 °C [146]. Additionally, *D. isaea* kills many hosts for food, thereby diminishing the yield of parasitoids. For example, at 27 °C, *D. isaea* females had a mean lifetime fecundity of 126 eggs but caused the death of 319 host larvae (through oviposition, host feeding and host stinging) [113].

To maximize the fertility of *H. varicornis* for mass rearing, suitable conditions for storing wasps were found to be, in priority: (i) female wasps at 15 °C for 10 days; (ii) female wasps at 15 °C for 20–30 days or 25 °C for 10–15 days; and (iii) 1-day-old pupae at 10 °C for 1–4 weeks [147]. For field releases, where host-killing is more important than fertility, suitable conditions for storing wasps were, in priority: (i) female wasps at 15 °C for 10–30 days or 25 °C for 10–15 days; and (ii) female wasps at 15 °C for 40 days or 1-day-old pupae at 10 °C for 1–4 weeks [147].

### 6.2. Quality Control of Mass-Reared Natural Enemies

Maintaining high-quality, mass-reared natural enemies is challenging. Selection for traits favoring production efficiency in mass-rearing facilities, such as reduced ability to disperse and high parasitism rates at high host density, can reduce effectiveness in the field, where high dispersal activity and high parasitism levels at low host density are preferred [94,148,149]. To overcome the rapid rate of laboratory adaptation in laboratory cultures, populations destined for field release should be reared under conditions that minimize field fitness loss and/or colonies should be renewed regularly from field material [150]. Quality control protocols for mass-reared arthropods used in augmentative releases in protected cropping have been implemented extensively in commercial insectaries in Europe and the USA [151,152].

### 6.3. Biased Sex Ratios

A major issue in mass rearing *D. isaea* is the overproduction of males. For example, samples from commercial insectaries showed a male proportion of 0.77 (*n* = 241) [153]. Assuming a conservative cost of USD 0.13 per wasp, the cost per female is approximately USD 0.57. This issue can be partly mitigated by manipulating host sizes offered to the wasps [154,155], but this approach adds complexity to the rearing system.

### 6.4. On-Farm Management

Leafminer damage can occur before parasitoids are able to reduce populations. In glasshouse chrysanthemum crops in the Netherlands, preventative introductions (five-weekly releases) of *D. isaea* still required an initial treatment with cyromazine (an insect growth regulator) to reduce initial numbers of leafminer larvae [156]. However, multiple cyromazine applications prevented the establishment of *D. isaea*. Establishing *D. isaea* was also difficult in winter when growth lights attracted and killed adult parasitoids [156]. Costs could be reduced by redistributing parasitoids produced early in the season to other parts of the glasshouse later in the season, effectively creating a within-crop rearing system [79,157], a method also used successfully in Kenya [158]. Growers can harvest heavily mined and parasitized milk thistle (*Sonchus arvensis*) plants from outside crops as a source of *D. isaea* [134]. This requires placing leaf material in small, screened cages (an augmentorium) with a mesh smaller than 625 μm [159,160], allowing parasitoids to exit while retaining leafminer flies.

Management issues for other pests can also drive adoption of leafminer biological control. For example, in Canada, biological control in ornamental crops has increased greatly in recent years due to difficulties controlling western flower thrips (*Frankliniella occidentalis* Pergande) resistant to spinosad [161]. Canadian chrysanthemum and gerbera growers largely rely on *D. isaea* releases because chemicals registered for control of *L. trifolii* harm predatory mites used to control thrips, as well as these chemicals becoming ineffective due to increasing resistance issues in *L. trifolii* [9].

## 7. Overcoming Barriers to Mass Rearing

Mass-produced agromyzid parasitoids are expensive when needed in large quantities because hosts must be reared and exposed to wasps on living plants. They are expected to be economical for protected cropping, particularly when multiple early releases of idiobiont wasps, such as *D. isaea*, *H. varicornis*, or *N. formosa*, are needed to ensure substantial host killing alongside parasitism. Koinobiont species such as *Opius* spp. do not host feed, and parasitized *Liriomyza* larvae continue mining leaves until pupation. In Australia, releasing *Opius* spp. in protected cropping would only be worthwhile for long-lived crops (where multiple generations of *Liriomyza* occur) or in situations where *Liriomyza* spp. overwinter in greenhouses.

*Diglyphus isaea* is widely reared and used overseas, largely because of its relatively high fecundity. While attempts to mass rear *H. varicornis* and *N. formosa* have been made overseas, these are seldom mass reared commercially. Based on the long-term experiences in Europe and the USA, we recommend *D. isaea* as the prime target parasitoid for mass rearing against *L. sativae.* Fortuitously, *D. isaea* is already present in Australia. It was first found in Sydney, NSW, in September 2001 [162] and has since been found in Victoria [30], Tasmania (P.M. Ridland, unpublished data) and the Australian Capital Territory [31], frequently parasitizing *P. syngenesiae* on sow thistle (*Sonchus oleraceus*) and *P. plantaginis* on *Plantago lanceolata* [34]. The species has been cultured in the laboratory in Australia [163] using *L. brassicae* reared on Chinese cabbage (*Brassica rapa* ssp. *pekinensis*) at 22 °C, with egg-adult development times of 13 days for females and 12 days for males.

As with other adventive biological agents in Australia, such as the aphid parasitoid, *Diaeretiella rapae* (McIntosh) [164], Australian founding populations of *D. isaea* likely have reduced genetic diversity compared to global populations. It would be important to test the biological characteristics of *D. isaea* populations from different Australian regions to select the most suitable strain for mass rearing [165].

While mortality caused by host feeding by idiobiont wasps is important for growers, commercial insectaries must maximize female wasp production from a given batch of host *Liriomyza* larvae. Male wasps are less useful since they do not paralyze larvae and only host-feed on larvae paralyzed by females [166].

To improve the economics of mass-reared parasitoids, thelytokous strains could be used, since all parasitized hosts will produce only females [167,168,169,170]. This benefits both growers and commercial insectaries since every wasp released will target *Liriomyza* larvae.

Thelytokous strains may result from endosymbiont infection or through host nuclear changes. Strains would need to be tested to ensure fecundity and longevity match or exceed those of arrhenotokous populations [171].

Thelytokous strains of *N. formosa* are associated with *Rickettsia* infection in Japan [172,173,174,175], China [176], and Australia [32,33]. Life table and host killing studies show thelytokous *N. formosa* have superior biocontrol potential compared to arrhenotokous populations [177]. However, in Japan, the thelytokous strain of *N. formosa* does not appear to have been widely utilized, with growers relying on *D. isaea* and *D. sibirica* imported from Europe [129]. In reported studies of bacterial endosymbionts infecting Eulophidae, *Rickettsia* infections were much more abundant (53%; *n* = 379) compared to *Wolbachia* (3.6%; *n* = 250) and *Cardinium* infections (0%; *n* = 215) [178]. These data come from 36 species from 20 genera, and may be skewed towards testing of observed thelytokous strains, with only a very small number of the >6000 described eulophid species tested [179].

In Australia, three eulophid species parasitizing agromyzids appear to be thelytokous: *N. formosa*, *Proacrias* sp. and *Aprostocetus* sp. [32,33]. All are infected with the endosymbiont *Rickettsia*; however, none of these species has high fecundity to match *D. isaea*. More detailed testing is needed to assess the extent of infection within each species and to treat lines with antibiotics to see if *Rickettsia* removal leads to arrhenotoky [180]. In Queensland, thelytokous *N. formosa* (infected with *Rickettsia*) and arrhenotokous *N. okazakii* (infected with the same *Rickettsia* and also a *Wolbachia* strain) were found in the same celery field [33]. Further studies are needed to determine if *Wolbachia* prevents thelytoky in *N. okazakii*, or if *N. formosa* might acquire the same *Wolbachia* strain. A female-only line of *D. isaea* could be the ideal, economically sustainable mass-rearing candidate. Transfer of thelytoky-inducing *Rickettsia* into Australian cultures of *D. isaea* through microinjection might be possible but is challenging [181].

Thelytoky can result from alleles in the insect’s own genome [182], and these alleles and their associated phenotypes may segregate within and between populations of the same species. For instance, arrhenotokous and thelytokous populations of *Anaphes diana* Girault, a mymarid parasitoid of *Sitona* spp., occur sympatrically in the Mediterranean region [167,183]. Recently, a thelytokous strain of *Diglyphus wani* Liu, Zhu & Yefremova was identified in Chinese horticultural regions, predominantly located at higher latitudes (36–43° N), whereas an arrhenotokous strain was observed at lower latitudes (25° N) [184,185]. This appears to be a case of geographic parthenogenesis [186] and suggests searching for thelytokous strains of species like *D. isaea* and *H. varicornis* could be focused on higher latitudes in Australia.

In the case of *D. wani*, life table analyses showed the thelytokous strain had a higher intrinsic growth rate (r_m_) and total host-killing ability than arrhenotokous strains [187]. Although a very low level (0.1%) of *Rickettsia* was detected in the thelytokous strain, there was no evidence that the endosymbiont induced the observed thelytoky; female wasps consistently produced only female offspring for five generations following tetracycline treatment or heat treatments aimed at eliminating *Rickettsia* [188,189], indicating that the *Rickettsia* infection was not responsible. Cytological examination and genetic analyses have confirmed this as the first documented case of apomictic thelytoky in the Eulophidae unrelated to endosymbionts [189].

## 8. Concluding Remarks

Australia’s rich endemic parasitoid fauna is likely to play a central role in controlling invasive, polyphagous *Liriomyza* spp. as they continue to spread into horticultural regions. Before considering the importation of exotic parasitoid species, it is essential to thoroughly assess the composition and impact of endemic parasitoid communities. Protecting these natural enemies from the overuse of non-selective insecticides and managing reservoirs of both parasitoids and non-target agromyzids remain critical priorities [27]. These principles apply equally if exotic parasitoids are introduced.

Improved taxonomic resolution of parasitoids attacking *Liriomyza* spp. and the development of molecular markers will enhance identification and impact assessment. Metabarcoding approaches offer a high-throughput means of screening large numbers of wasp samples and tracking community dynamics.

Advances in mass rearing may come from the identification of thelytokous strains or manipulating parasitoids with endosymbionts to create novel thelytokous strains. The suitability of these strains must be assessed through tests of fecundity, host feeding, thermal biology, endosymbiont status, searching efficiency and parasitism efficiency at low host density [86,187]. Modern genetic and genomic tools also offer opportunities to improve the efficacy of existing and new biocontrol agents [190].

While adventive species like *D. isaea* are likely to play an important role in biocontrol, potentially valuable endemic species should not be overlooked. For example, the capacity of *L. trifolii* and *L. sativae* to encapsulate eggs of endemic Australian *Opius* species remains unknown [63], despite *O. cinerariae* being a common parasitoid of *L. huidobrensis* in Queensland [33]. *Opius* attack a broad host range of agromyzids in Australia, including *L. brassicae*, *P. plantaginis* and *P. syngenesiae* [27], and *O. cinerariae* has already been successfully reared in the laboratory using *L. brassicae* as a host [29]. Likewise, endemic eulophids such as *Z. latilineatum* and *C. mirabilis*, and the pteromalid, *T. parasitica,* warrant closer study for their potential as effective biocontrol agents [27].

## Figures and Tables

**Table 1:** Comprehensive lists of parasitoids recorded from polyphagous *Liriomyza* species worldwide.

Reference	Description
Waterhouse and Norris (1987) [47]	List of parasitoids of *L. huidobrensis*, *L. sativae* and *L. trifolii.* They differentiate between parasitoids identified to species or only to genus. They also provide lists of parasitoids reared from ‘*Liriomyza* spp.’ or ‘*Liriomyza*. sp.’ (implying *L. sativae*, *L. trifolii* or *L. huidobrensis* or mixed populations of two or three of these). Note that they include records of *Liriomyza langei* Frick from California and Hawaii as *L. huidobrensis*.
Grenouillet et al. (1993) [35]	A world list of all known parasites and predators of the 26 economically important species of *Liriomyza* is given. Relatively few records of parasitoids of *L. huidobrensis*, since the review was completed before the major expansion of *L. huidobrensis* worldwide. Because of space limitations, the majority of the 69 references listed were not linked by the authors to individual species.
Vega (2003) [48]	Lists of 73 parasitoid species reared from *Liriomyza bryoniae* (Kaltenbach), *Liriomyza strigata* (Meigen), *L. huidobrensis* and *L. trifolii*. No references given and nomenclature is outdated.
Liu et al. (2011) [49]	This review provides a list of 143 parasitoid species recorded from *Liriomyza* spp. but without references for individual species. Nomenclature is outdated for many species.
Weintraub et al. (2017) [4]	This is a very comprehensive record of parasitoids of *L. huidobrensis*, with host plant records for nearly all records. Primary references are cited.
Ridland et al. (2020) [27]	Seventy-one parasitoid species recorded from *L. sativae* worldwide (50 species of Eulophidae; 12 species of Braconidae; 5 species of Pteromalidae; 5 species of Figitidae). Primary references are cited.
UCD Community (2023) [50]	The Universal Chalcidoidea Database provides an exhaustive catalog of Chalcidoidea, with extensive references and up-to-date nomenclature. *L. huidobrensis* (30 species of Eulophidae; 8 species of Pteromalidae), *L. sativae* (39 species of Eulophidae; 4 species of Pteromalidae) and *L. trifolii* (45 species of Eulophidae; 6 species of Pteromalidae; 1 species of Tetracampidae).

**Table 2:** Hymenopteran parasitoids reared from *L. huidobrensis* (Lh), *L. trifolii* (Lt) and *L. sativae* (Ls) worldwide summarized by family and sub-family. Data for all parasitoid species are listed in Table S4.

Family/ Sub-Family		Lh		Lt		Ls		∑ ^1^	Pooled ^2^		Overlap ^3^	
	*n*	%	*n*	%	*n*	%	*n*	%	*n*	%	*n*	%
**Eulophidae**	**49**	**66%**	**61**	**66%**	**50**	**69%**	**160**	**67%**	**92**	**63%**	**68**	**43%**
Eulophinae	27	36%	29	32%	26	36%	82	34%	44	30%	38	46%
Entedoninae	21	28%	28	30%	24	33%	73	31%	44	30%	29	40%
Tetrastichinae	1	1%	4	4%	0	0%	5	2%	4	3%	1	20%
**Braconidae**	**13**	**18%**	**13**	**14%**	**12**	**17%**	**38**	**16%**	**27**	**19%**	**11**	**29%**
Opiinae	10	14%	10	11%	12	17%	32	13%	22	15%	10	31%
Alysiinae	2	3%	3	3%	0	0%	5	2%	4	3%	1	20%
Braconinae	1	1%		0%	0	0%	1	0%	1	1%	0	0%
**Pteromalidae**	**7**	**9%**	**8**	**9%**	**5**	**7%**	**20**	**8%**	**13**	**9%**	**7**	**35%**
Miscogastrinae	6	8%	4	4%	4	6%	14	6%	8	6%	6	43%
Pteromalinae	1	1%	4	4%	1	1%	6	3%	5	3%	1	17%
**Figitidae**	**4**	**5%**	**9**	**10%**	**5**	**7%**	**18**	**8%**	**11**	**8%**	**7**	**39%**
Eucoilinae	4	5%	9	10%	5	7%	18	8%	11	8%	7	39%
**Tetracampidae**	**1**	**1%**	**1**	**1%**	**0**	**0%**	**2**	**1%**	**2**	**1%**	**0**	**0%**
Tetracampinae	0	0%	1	1%	0	0%	1	0%	1	1%	0	0%
Platynocheilinae	1	1%	0	0%	0	0%	1	0%	1	1%	0	0%
**∑**	**74**		**92**		**72**		**238**		**145**		**93**	

^1^ Sum of all named species reared from each *Liriomyza* host species. ^2^ Pooled species reared from all three *Liriomyza* species (Note: this total is not the sum of species from each individual *Liriomyza* species, as some parasitoid species occurred in more than one *Liriomyza* host species). ^3^ Overlapping species (∑-pooled), representing those reared from more than one *Liriomyza* host species.

**Table 3:** Overlap of parasitoid species across agromyzid hosts. Data for all parasitoid species are listed in Table S4.

	*L. huidobrensis*		*L. trifolii*		*L. sativae*	
Species Overlap	*n*	%	*n*	%	*n*	%
Lh/Lt/Ls	27	36%	27	29%	27	38%
Lh/Lt	11	15%	11	12%	NA	NA
Lh/Ls	12	16%	NA	NA	12	17%
Lt/Ls	NA	NA	16	17%	16	22%
Lh only	24	32%	NA	NA	NA	NA
Lt only	NA	NA	38	41%	NA	NA
Ls only	NA	NA	NA	NA	17	24%
∑	74		92		72	

Lh = *L. huidobrensis*, Lt = *L. trifolii*, Ls = *L. sativae*. NA = not applicable (comparisons not applicable to a particular host species).

**Table 4:** Most commonly recorded parasitoid genera by location records in descending order (pooling all records for a particular location for each *Liriomyza* species).

Family	Sub-Family	Genus	*L. huidobrensis*	*L. trifolii*	*L. sativae*	∑	% ^1^
Eulophidae	Eulophinae	*Diglyphus*	48	35	21	104	18%
Braconidae	Opiinae	*Opius * ^2^	31	30	17	78	16%
Eulophidae	Entedoninae	*Chrysocharis*	30	19	18	67	11%
Eulophidae	Entedoninae	*Neochrysocharis*	14	34	15	63	11%
Eulophidae	Eulophinae	*Hemiptarsenus*	14	19	11	44	7%
Pteromalidae	Miscogastrinae	*Halticoptera*	17	12	6	35	6%
Eulophidae	Entedoninae	*Closterocerus*	5	6	5	16	3%
Eulophidae	Eulophinae	*Pnigalio*	6	8	1	15	3%
Eulophidae	Eulophinae	*Zagrammosoma*	4	6	5	15	3%
Eulophidae	Entedoninae	*Asecodes*	3	5	4	12	2%
Figitidae	Eucoilinae	*Gronotoma*	5	4	2	11	2%
Braconidae	Alysiinae	*Dacnusa*	9	2	0	11	2%
Eulophidae	Entedoninae	*Chrysonotomyia*	2	7	1	10	2%
Eulophidae	Eulophinae	*Diaulinopsis*	3	4	3	10	2%

^1^ Percentage of all location records (588). ^2^ *Phaedrotoma* has recently been synonymized with *Opius* [57].

**Table 5:** Most commonly recorded parasitoid species of *L. huidobrensis*, *L. trifolii* and *L. sativae* by location records in descending order (excluding specimens only identified to genus; pooling all records for a particular location for each *Liriomyza* sp.). (Full data set in Table S5).

Family	Sub-Family	*Species*	*L. huidobrensis*	*L. trifolii*	*L. sativae*	∑
Eulophidae	Eulophinae	*Diglyphus isaea*	15	12	5	32
Eulophidae	Entedoninae	*Neochrysocharis* *formosa*	7	16	7	30
Eulophidae	Eulophinae	*Hemiptarsenus* *varicornis*	6	11	9	26
Eulophidae	Entedoninae	*Chrysocharis pentheus*	5	5	5	15
Eulophidae	Eulophinae	*Diglyphus crassinervis*	5	6	2	13
Braconidae	Opiinae	*Opius dissitus*	4	5	3	12
Pteromalidae	Miscogastrinae	*Halticoptera circulus*	5	5	2	12
Eulophidae	Entedoninae	*Neochrysocharis* *okazakii*	3	5	3	11
Braconidae	Opiinae	*Opius dimidiatus*	2	4	3	9
Eulophidae	Eulophinae	*Hemiptarsenus* *zilahisebessi*	3	5	1	9
Eulophidae	Eulophinae	*Diglyphus begini*	4	2	3	9
Eulophidae	Entedoninae	*Chrysocharis caribea*	2	2	4	8
Eulophidae	Entedoninae	*Chrysocharis oscinidis*	1	4	3	8

**Table 6:** Indicative release rates of *Diglyphus isaea* [Miglyphus^®^] [143].

Miglyphus^®^	Light Infestation	Heavy Infestation
Rate m^2^/unit (500 wasps)	2000	500
Rate wasps/m^2^	0.25	1
Rate wasps/ha	2500	10,000
Interval (days)	7	7
Frequency	min. 3×	min. 3×
∑wasps/ha	7500	30,000
Remarks	if >1 larva/10 plants	if >1 larva/3 plants

## Data Availability

No new data were created in this study. Data sharing is not applicable to this article.

## Supplementary Materials

The following supporting information can be downloaded at: https://www.mdpi.com/article/10.3390/insects16090968/s1, Table S1: Parasitoid species recorded from *Liriomyza trifolii*; Table S2: Parasitoid species recorded from *Liriomyza huidobrensis*; Table S3: Parasitoid species recorded from *Liriomyza sativae*; Table S4: Overlap of parasitoid species recorded from the three Liriomyza species; Table S5: Parasitoid species by location records in descending order (pooling all records for a particular location for each Liriomyza species).

## References

[1] Lonsdale O., Murphy S.T., Scheffer S.J. (2023). Agromyzidae (Diptera) Plant Pests.

[2] Minkenberg O.P.J.M. (1988). Dispersal of *Liriomyza trifolii*. Bull. OEPP/EPPO Bull..

[3] Spencer K.A., Kahn R.P. (1989). Leaf miners. Plant Protection and Quarantine Volume. II Selected Pests and Pathogens of Quarantine Significance.

[4] Weintraub P.G., Scheffer S.J., Visser D., Valladares G., Correa A.S., Shepard B.M., Rauf A., Murphy S.T., Mujica N., MacVean C. (2017). The invasive *Liriomyza huidobrensis* (Diptera: Agromyzidae): Understanding its pest status and management globally. J. Insect Sci..

[5] van der Staay M. (1992). Chemical control of the larvae of the leafminer *Liriomyza huidobrensis* (Blanchard) in lettuce. Meded. Van De Fac. Landbouwwet. Univ. Gent.

[6] Leibee G.L., Capinera J.L. (1995). Pesticide resistance in Florida insects limits management options. Fla. Entomol..

[7] Ferguson J.S. (2004). Development and stability of insecticide resistance in the leafminer *Liriomyza trifolii* (Diptera: Agromyzidae) to cyromazine, abamectin, and spinosad. J. Econ. Entomol..

[8] Reitz S., Gao Y., Lei Z., Trdan S. (2013). Insecticide use and the ecology of invasive *Liriomyza* leafminer management. Insecticides—Development of Safer and More Effective Technologies.

[9] Conroy L., Scott-Dupree C., Harris C., Murphy G., Broadbent A. (2008). Susceptibility of two strains of American serpentine leafminer (*Liriomyza trifolii* (Burgess)) to registered and reduced risk insecticides in Ontario. J. Entomol. Soc. Ont..

[10] Nguyen D.T., Chen Y.Z., Herron G.A. (2024). Insecticide resistance in Australian populations of the serpentine leaf miner *Liriomyza huidobrensis* (Blanchard) (Diptera: Agromyzidae). Austral Entomol..

[11] Gao Y.L., Reitz S.R., Wei Q.B., Yu W.Y., Lei Z.R. (2012). Insecticide-mediated apparent displacement between two invasive species of leafminer fly. PLoS ONE.

[12] Gao Y.L., Reitz S., Xing Z.L., Ferguson S., Lei Z.R. (2017). A decade of leafminer invasion in China: Lessons learned. Pest Manag. Sci..

[13] Bolton M. (1986). Die Amerikaanse blaarmyner: ‘n belangrike plaag by tamaties. Roodeplaat Bull..

[14] Foba C.N., Salifu D., Lagat Z.O., Gitonga L.M., Akutse K.S., Fiaboe K.K.M. (2015). Species composition, distribution, and seasonal abundance of *Liriomyza* leafminers (Diptera: Agromyzidae) under different vegetable production systems and agroecological zones in Kenya. Environ. Entomol..

[15] Potatoes South Africa (2017). Descriptions of 16 Potato Pests in South Africa.

[16] Mugala T., Visser D., Malan A.P., Addison P. (2022). Review of *Liriomyza huidobrensis* (Blanchard, 1926) (Diptera: Agromyzidae) on potatoes in South Africa, with special reference to biological control using entomopathogens and parasitoids. Afr. Entomol..

[17] International Plant Protection Convention Detection of *Liriomyza sativae* in Far North Queensland. IPPC Pest Report, AUS 80/1. https://www.ippc.int/en/countries/Australia/pestreports/2017/04/detection-of-liriomyza-sativae-in-far-north-queensland/.

[18] International Plant Protection Convention *Liriomyza sativae* in Torres Strait. IPPC Pest Report AUS-65/2. https://www.ippc.int/en/countries/Australia/pestreports/2014/10/liriomyza-sativae-in-torres-strait-/.

[19] Blacket M.J., Rice A.D., Semeraro L., Malipatil M.B. (2015). DNA-based identifications reveal multiple introductions of the vegetable leafminer *Liriomyza sativae* (Diptera: Agromyzidae) into the Torres Strait Islands and Papua New Guinea. Bull. Entomol. Res..

[20] International Plant Protection Convention Liriomyza huidobrensis (serpentine leafminer) in New South Wales and Queensland. IPPC Pest Report AUS 103/3. https://www.ippc.int/en/countries/australia/pestreports/2021/07/liriomyza-huidobrensis-in-new-south-wales-and-queensland/.

[21] Mulholland S., Gopurenko D., Mirrington R., Löcker B., Gillespie P., Rossiter L., Anderson C. (2022). First report of the serpentine leafminer *Liriomyza huidobrensis* (Blanchard) (Diptera: Agromyzidae) and its impacts in Australia. Austral Entomol..

[22] International Plant Protection Convention *Liriomyza trifolii* (American serpentine leafminer) in Queensland and Western Australia. IPPC Pest Report AUS 104/1. https://www.ippc.int/en/countries/australia/pestreports/2021/07/liriomyza-trifolii-american-serpentine-leafminer-in-queensland-and-western-australia/.

[23] AUSVEG (2025). American Serpentine Leafminer ASLM Detected in the Lockyer Valley. https://ausveg.com.au/knowledge-hub/american-serpentine-leafminer-aslm-detected-in-the-lockyer-valley/.

[24] Xu X.-F., Coquilleau M.P., Ridland P.M., Umina P.A., Yang Q., Hoffmann A.A. (2021). Molecular identification of leafmining flies from Australia including new *Liriomyza* outbreaks. J. Econ. Entomol..

[25] Xu X.-F., Ridland P.M., Umina P.A., Gill A., Ross P.A., Pirtle E., Hoffmann A.A. (2021). High incidence of related *Wolbachia* across unrelated leaf-mining Diptera. Insects.

[26] Murphy S.T., LaSalle J. (1999). Balancing biological control strategies in the IPM of New World invasive *Liriomyza* leafminers in field vegetable crops. Biocontrol News Inf..

[27] Ridland P.M., Umina P.A., Pirtle E.I., Hoffmann A.A. (2020). Potential for biological control of the vegetable leafminer, *Liriomyza sativae* (Diptera: Agromyzidae), in Australia with parasitoid wasps. Austral Entomol..

[28] Kleinschmidt R.P. (1970). Studies of some Agromyzidae in Queensland. Qld. J. Agric. Anim. Sci..

[29] Lardner R.M. (1991). Comparative Host Stage Utilization of Two Parasitoids of *Liriomyza brassicae* (Diptera: Agromyzidae). Ph.D. Thesis.

[30] Bjorksten T.A., Robinson M., La Salle J. (2005). Species composition and population dynamics of leafmining flies and their parasitoids in Victoria. Aust. J. Entomol..

[31] Lambkin C.L., Fayed S.A., Manchester C., La Salle J., Scheffer S.J., Yeates D.K. (2008). Plant hosts and parasitoid associations of leaf mining flies (Diptera: Agromyzidae) in the Canberra region of Australia. Aust. J. Entomol..

[32] Xu X.-F., Hoffmann A.A., Umina P.A., Coquilleau M.P., Gill A., Ridland P.M. (2022). Identification of two leafminer parasitoids (Hymenoptera: Eulophidae), *Neochrysocharis formosa* and *Proacrias* sp. from Australia, with both showing thelytoky and infection by *Rickettsia*. Austral Entomol..

[33] Xu X.-F., Hoffmann A.A., Umina P.A., Ward S.E., Coquilleau M.P., Malipatil M.B., Ridland P.M. (2023). Molecular identification of hymenopteran parasitoids and their endosymbionts from agromyzids. Bull. Entomol. Res..

[34] Coquilleau M.P., Ridland P.M., Xu X.-F., Umina P.A., Hoffmann A.A. (2025). The abundance and phenology of four common agromyzid leafmining flies (Diptera: Agromyzidae) and their associated parasitoid wasps in southern Victoria. Austral Entomol..

[35] Grenouillet C., Martinez M., Rasplus J.Y., Bordat D., Coly E.V., Ferron P., LeClant F., Martinez M., Martin C., Mestres R., Theissen G., Vercambre B. (1993). Liste des parasitoïdes et des prédateurs des *Liriomyza* d’importance economique dans le monde (Diptera: Agromyzidae). Liriomyza: Colloque sur les Mouches Mineuses des Plantes Cultivees, 24–26 Mar 1993.

[36] Noyes J.S. (1994). The reliability of published host-parasitoid records: A taxonomist’s view. Nor. J. Agric. Sci. Suppl..

[37] Shaw M.R., Hawkins B.A., Sheehan W. (1994). Parasitoid host ranges. Parasitoid Community Ecology.

[38] Spencer K.A. (1965). A clarification of the status of *Liriomyza trifolii* (Burgess) and some related species. Proc. Entomol. Soc. Wash..

[39] Spencer K.A. (1973). Agromyzidae (Diptera) of Economic Importance.

[40] Fisher N., La Salle J. (2005). A new species of *Neochrysocharis* Kurdjumov (Hymenoptera: Eulophidae), a parasitoid of serpentine leafminers (Diptera: Agromyzidae) in Southeast Asia. Zootaxa.

[41] Hernández R., Harris M., Crosby K., Liu T.X. (2010). *Liriomyza* (Diptera: Agromyzidae) and parasitoid species on pepper in the lower Rio Grande Valley of Texas. Southwest. Entomol..

[42] Hansson C., Navone P. (2017). Review of the European species of *Diglyphus* Walker (Hymenoptera: Eulophidae) including the description of a new species. Zootaxa.

[43] Sha Z.-L., Zhu C.-D., Murphy R.W., La Salle J., Huang D.-W. (2006). Mitochondrial phylogeography of a leafminer parasitoid, *Diglyphus isaea* (Hymenoptera: Eulophidae) in China. Biol. Control..

[44] Sha Z.-L., Zhu C.-D., Murphy R.W., Huang D.-W. (2007). *Diglyphus isaea* (Hymenoptera: Eulophidae): A probable complex of cryptic species that forms an important biological control agent of agromyzid leaf miners. J. Zool. Syst. Evol. Res..

[45] Lue C.H., Buffington M.L., Scheffer S., Lewis M., Elliott T.A., Lindsey A.R., Driskell A., Jandova A., Kimura M.T., Carton Y. (2021). DROP: Molecular voucher database for identification of *Drosophila* parasitoids. Mol. Ecol. Resour..

[46] Lue C.H., Abram P.K., Hrcek J., Buffington M.L., Staniczenko P.P.A. (2023). Metabarcoding and applied ecology with hyperdiverse organisms: Recommendations for biological control research. Mol. Ecol..

[47] Waterhouse D.F., Norris K.R. (1987). Biological Control: Pacific Prospects.

[48] Vega P.B. (2003). Dípteros de interés agronómico. Agromícidos plaga de cultivos hortícolas intensivos. Boletín De La Soc. Entomológica Aragonesa.

[49] Liu T., Kang L., Lei Z., Hernández R., Liu T., Kang L. (2011). Hymenopteran parasitoids and their role in biological control of vegetable *Liriomyza* leafminers. Recent Advances in Entomological Research: From Molecular Biology to Pest Management.

[50] UCD Community (2023). Universal Chalcidoidea Database Website. https://ucd.chalcid.org.

[51] Liu T.-X., Kang L., Heinz K.M., Trumble J. (2009). Biological control of *Liriomyza* leafminers: Progress and perspective. CABI Rev..

[52] Weber D.C., Hajek A.E., Hoelmer K.A., Mason P.G., Gillespie D.R., Vincent C. (2017). Accidental introductions of natural enemies: Causes and implications. Proceedings of the 5th International Symposium on Biological Control of Arthropods.

[53] Abram P.K., McPherson A.E., Kula R., Hueppelsheuser T., Thiessen J., Perlman S.J., Curtis C.I., Fraser J.L., Tam J., Carrillo J. (2020). New records of *Leptopilina*, *Ganaspis*, and *Asobara* species associated with *Drosophila suzukii* in North America, including detections of *L. japonica* and *G. brasiliensis*. J. Hymenopt. Res..

[54] Puppato S., Grassi A., Pedrazzoli F., De Cristofaro A., Ioriatti C. (2020). First report of *Leptopilina japonica* in Europe. Insects.

[55] Godfray H.C.J., Agassiz D.J.L., Nash D.R., Lawton J.H. (1995). The recruitment of parasitoid species to two invading herbivores. J. Anim. Ecol..

[56] Cornell H.V., Hawkins B.A. (1993). Accumulation of native parasitoid species on introduced herbivores—A comparison of hosts as natives and hosts as invaders. Am. Nat..

[57] Burgio G., Lanzoni A., Navone P., Van Achterberg K., Masetti A. (2007). Parasitic Hymenoptera fauna on agromyzidae (Diptera) colonizing weeds in ecological compensation areas in northern Italian agroecosystems. J. Econ. Entomol..

[58] Bouček Z. (1988). Australasian Chalcidoidea (Hymenoptera). A Biosystematic Revision of Genera of Fourteen Families, with a Reclassification of Species.

[59] Chien C.-C., Chang S.-C. (2008). Morphology and life history of *Chrysocharis pentheus* (Walker) (Hymenoptera: Eulophidae). Formos. Entomol..

[60] Chien C.-C., Chang S.-C. (2013). Effect of temperature on killing of *Liriomyza huidobrensis* (Diptera: Agromyzidae) by the parasitoids *Chrysocharis pentheus* and *Closterocerus okazakii* (Hymenoptera: Eulophidae). J. Taiwan Agric. Res..

[61] Sasaki F., Ueno T. (2016). Development and sex ratio of the parasitoid *Chrysocharis pentheus* (Hymenoptera: Eulophidae) on the leafminer *Liriomyza trifolii* (Diptera: Agromyzidae). J. Fac. Agric. Kyushu Univ..

[62] Baeza Larios G.L., Ohno K., Fukuhara F. (2007). Effects of photoperiod and temperature on preimaginal development and summer diapause of *Chrysocharis pubicornis* (Zetterstedt) (Hymenoptera: Eulophidae), a pupal parasitoid of leafminers (Diptera: Agromyzidae). Appl. Entomol. Zool..

[63] Belokobylskij S.A., Wharton R.A., La Salle J. (2004). Australian species of the genus *Opius* Wesmael (Hymenoptera: Braconidae) attacking leaf-mining Agromyzidae, with the description of a new species from South-east Asia. Aust. J. Entomol..

[64] Gates M.W., Heraty J.M., Schauff M.E., Wagner D.L., Whitfield J.B., Wahl D.B. (2002). Survey of the parasitic Hymenoptera on leafminers in California. J. Hymenopt. Res..

[65] Griffiths G.C.D. (1966). The Alysiinae (Hym. Braconidae) parasites of the Agromyzidae (Diptera). III. The parasites of *Paraphytomyza* Enderlein, *Phytagromyza* Hendel and *Phytomyza* Fallén. Beiträge Zur Entomol..

[66] Wharton R.A., Austin A.D. (1991). Revision of Australian Dacnusini (Hymenoptera: Braconidae: Alysiinae), parasitoids of cyclorrhaphous Diptera. Aust. J. Entomol..

[67] Paretas-Martínez J., Forshage M., Buffington M., Fisher N., La Salle J., Pujade-Villar J. (2013). Overview of Australian Cynipoidea (Hymenoptera). Aust. J. Entomol..

[68] Salvo A., Valladares G. (1995). Complejo parasítico (Hymenoptera: Parasitica) de *Liriomyza huidobrensis* Diptera: Agromyzidae) en haba. Agriscientia.

[69] Salvo A., Valladares G. (2002). Plant-related intraspecific size variation in parasitoids (Hymenoptera: Parasitica) of a polyphagous leafminer (Diptera: Agromyzidae). Environ. Entomol..

[70] Mujica N., Kroschel J. (2011). Leafminer fly (Diptera: Agromyzidae) occurrence, distribution, and parasitoid associations in field and vegetable crops along the Peruvian coast. Environ. Entomol..

[71] Li X.Y., van Achterberg C., Tan J.C. (2013). Revision of the subfamily Opiinae (Hymenoptera, Braconidae) from Hunan (China), including thirty-six new species and two new genera. ZooKeys.

[72] van Achterberg C. (2023). Illustrated key to the European genera of Opiinae (Hymenoptera, Braconidae), with the description of two new Palaearctic genera and two new species. ZooKeys.

[73] Muchemi S.K., Zebitz C.P.W., Borgemeister C., Akutse K.S., Foba C.N., Ekesi S., Fiaboe K.K.M. (2018). Interaction between *Chrysocharis flacilla* and *Diglyphus isaea* (Hymenoptera: Eulophidae), two parasitoids of *Liriomyza* leafminers. J. Econ. Entomol..

[74] Muchemi S.K., Zebitz C.P.W., Borgemeister C., Akutse K.S., Foba C.N., Ekesi S., Fiaboe K.K.M. (2018). Acceptability and suitability of three *Liriomyza* species as host for the endoparasitoid *Halticoptera arduine* (Hymenoptera: Pteromalidae). Environ. Entomol..

[75] Muchemi S.K., Zebitz C.P.W., Borgemeister C., Akutse K.S., Foba C.N., Ekesi S., Fiaboe K.K.M. (2018). Acceptability and suitability of three *Liriomyza* leafminer species as host for the endoparasitoid *Chrysocharis flacilla* (Hymenoptera: Eulophidae). J. Econ. Entomol..

[76] Muchemi S.K., Zebitz C.P.W., Borgemeister C., Akutse K.S., Foba C.N., Ekesi S., Fiaboe K.K.M. (2018). Interaction between two leafminer parasitoids, *Halticoptera arduine* (Hymenoptera: Pteromalidae) and *Diglyphus isaea* (Hymenoptera: Eulophidae), in the management of *Liriomyza huidobrensis* (Diptera: Agromyzidae). Environ. Entomol..

[77] Foba C.N., Salifu D., Lagat Z.O., Gitonga L.M., Akutse K.S., Fiaboe K.K.M. (2016). *Liriomyza* leafminer (Diptera: Agromyzidae) parasitoid complex in different agroecological zones, seasons, and host plants in Kenya. Environ. Entomol..

[78] van Lenteren J.C., Bale J., Bigler F., Hokkanen H.M.T., Loomans A.J.M. (2006). Assessing risks of releasing exotic biological control agents of arthropod pests. Annu. Rev. Entomol..

[79] Jacobson R. (2010). Boosting Biocontrols Within IPM Programmes. Factsheet Protected Edibles PC 240.

[80] Palumbo J.C., Castle S.J. (2009). IPM for fresh-market lettuce production in the desert southwest: The produce paradox. Pest Manag. Sci..

[81] Palumbo J.C. (2023). Trends in insect losses and management on desert lettuce: A 19-year summary. UA VegIPM Update.

[82] Wardlow L.R. (1984). Monitoring the activity of tomato leafminer (*Liriomyza bryoniae* Kalt.) and its parasites in commercial glasshouses in Southern England. Meded. Van De Fac. Landbouwwet. Rijksuniv. Gent.

[83] Minkenberg O.P.J.M., van Lenteren J.C. (1986). The leafminers *Liriomyza bryoniae* and *L. trifolii* (Diptera: Agromyzidae), their parasites and host plants: A review. Agric. Univ. Wagening. Pap..

[84] Parrella M.P., Hansen L.S., van Lenteren J., Bellows T.S., Fisher T.W. (1999). Glasshouse Environments. Handbook of Biological Control.

[85] van Lenteren J.C., Ramakers P.M.J., Woets J. (1979). The biological control situation in Dutch glasshouses: Problems with *Trialeurodes vaporariorum* (Westwood), *Liriomyza bryoniae* Kalt. and *Myzus persicae* Sulz. Meded. Fac. Landbouwwet. Rijksuniv. Gent.

[86] Minkenberg O.P.J.M., van Lenteren J.C. (1990). Evaluation of parasitoids for the biological control of leafminers on glasshouse tomatoes: Development of a preintroduction selection procedure. SROP/WPRS Bull..

[87] Hendrikse A., Zucchi R. (1979). The importance of observing parasite behaviour for the development of biological control of the tomato leafminer (*Liriomyza bryoniae* Kalt.). Meded. Van De Fac. Landbouwwet. Rijksuniv. Gent.

[88] Woets J., van der Linden A. (1982). On the occurrence of *Opius pallipes* Wesmael and *Dacnusa sibirica* Telenga (Braconidae) in cases of natural control of the tomato leafminer *Liriomyza bryoniae* Kalt. (Agromyzidae) in some large greenhouses in the Netherlands. Meded. Van De Fac. Landbouwwet. Rijksuniv. Gent.

[89] Johnson M.W., Oatman E.R., Wyman J.A. (1980). Natural control of *Liriomyza sativae* [Dip: Agromyzidae] in pole tomatoes in southern California. Entomophaga.

[90] Woets J., van der Linden A. (1985). First experiments on *Chrysocharis parksi* Crawford (Hym.: Eulophidae) as a parasite for leafminer control (*Liriomyza* spp.) (Dipt.: Agromyzidae) in European greenhouse tomatoes. Meded. Van De Fac. Landbouwwet. Rijksuniv. Gent.

[91] Frijters A., Minkenberg O., Woets J., Ravensberg W. (1986). *Chrysocharis parksi* in commercial greenhouses for the biological control of leafminers, *Liriomyza bryoniae* and *L. trifolii*, on tomatoes: Case studies and sampling techniques. Meded. Van De Fac. Landbouwwet. Rijksuniv. Gent.

[92] van der Linden A. (1986). Addition of the exotic leaf miner parasites *Chrysocharis parksi* and *Opius dimidiatus* to the native Dutch parasite complex on tomato. Meded. Van De Fac. Landbouwwet. Univ. Gent.

[93] van der Linden A. (1990). Survival of the leafminer parasitoids *Chrysocharis oscinidis* Ashmead and *Opius pallipes* Wesmael after cold storage of host pupae. Meded. Van De Fac. Landbouwwet. Univ. Gent.

[94] van Lenteren J.C., Tommasini M.G., Albajes R., Lodovica Gullino M., van Lenteren J.C., Elad Y. (1999). Mass production, storage, shipment and quality control of natural enemies. Integrated Pest and Disease Management in Greenhouse Crops.

[95] van Lenteren J.C. (2012). The state of commercial augmentative biological control: Plenty of natural enemies, but a frustrating lack of uptake. Biocontrol.

[96] Cock M.J.W., van Lenteren J.C., Brodeur J., Barratt B.I.P., Bigler F., Bolckmans K., Cônsoli F.L., Haas F., Mason P.G., Parra J.R.P. (2010). Do new Access and Benefit Sharing procedures under the Convention on Biological Diversity threaten the future of biological control?. Biocontrol.

[97] van Lenteren J.C., Bolckmans K., Köhl J., Ravensberg W.J., Urbaneja A. (2018). Biological control using invertebrates and microorganisms: Plenty of new opportunities. Biocontrol.

[98] Costa-Lima T.C., Chagas M.C.M., Parra J.R.P. (2014). Temperature-dependent development of two neotropical parasitoids of *Liriomyza sativae* (Diptera: Agromyzidae). J. Insect Sci..

[99] Costa-Lima T.C., Chagas M.C.M., Parra J.R.P. (2019). Comparing potential as biocontrol agents of two neotropical parasitoids of *Liriomyza sativae*. Neotrop. Entomol..

[100] Hondo T., Koike A., Sugimoto T. (2006). Comparison of thermal tolerance of seven native species of parasitoids (Hymenoptera: Eulophidae) as biological control agents against *Liriomyza trifolii* (Diptera: Agromyzidae) in Japan. Appl. Entomol. Zool..

[101] Bjorksten T.A. (2005). Evaluating potential for field release of the leafminer parasitoid, *Hemiptarsenus varicornis*, to control *Liriomyza brassicae* in the field. ACIAR CP/2000/090 Res. Rep. 2003 2004 Liriomyza Huidobrensis Leafminer Dev. Eff. Pest Manag. Strateg. Indones. Aust..

[102] Bjorksten T.A. (2005). Evaluating the potential of *Diglyphus isaea* and *Hemiptarsenus varicornis* for glasshouse release to control *Liriomyza brassicae*. ACIAR CP/2000/090 Res. Rep. 2003 2004 Liriomyza Huidobrensis Leafminer Dev. Eff. Pest Manag. Strateg. Indones. Aust..

[103] Bethke J.A., Parrella M.P. (1985). Leaf puncturing, feeding and oviposition behavior of *Liriomyza trifolii*. Entomol. Exp. et Appl..

[104] Choi I., Kim J., Kim G., Kim C. (2003). Injury aspects of the stone leek leafminer, *Liriomyza chinensis* Kato (Diptera: Agromyzidae) on Welsh onion. Korean J. Appl. Entomol..

[105] Trumble J.T. (1981). *Liriomyza trifolii* could become a problem on celery. Calif. Agric..

[106] Leibee G.L. (1984). Influence of temperature on development and fecundity of *Liriomyza trifolii* (Burgess) (Diptera: Agromyzidae) on celery. Environ. Entomol..

[107] Parrella M.P. (1987). Biology of *Liriomyza*. Annu. Rev. Entomol..

[108] Petitt F.L. (1991). Effect of photoperiod on larval emergence and adult eclosion rhythms in *Liriomyza sativae* (Diptera: Agromyzidae). Fla. Entomol..

[109] Haghani M., Fathipour Y., Talebi A.A., Baniamer V. (2007). Thermal requirement and development of *Liriomyza sativae* (Diptera: Agromyzidae) on cucumber. J. Econ. Entomol..

[110] Petitt F.L., Allen J.C., Barfield C.S. (1991). Degree-day model for vegetable leafminer (Diptera: Agromyzidae) phenology. Environ. Entomol..

[111] Heinz K.M., Parrella M.P. (1989). Attack behavior and host size selection by *Diglyphus begini* on *Liriomyza trifolii* in chrysanthemum. Entomol. Exp. Appl..

[112] Patel K.J., Schuster D.J., Smerage G.H. (2003). Density dependent parasitism and host-killing of *Liriomyza trifolii* (Diptera: Agromyzidae) by *Diglyphus intermedius* (Hymenoptera: Eulophidae). Fla. Entomol..

[113] Zhang Y.B., Lu S.L., Liu W.X., Wang W.X., Wang W., Wan F.H. (2014). Comparing immature development and life history traits in two coexisting host-feeding parasitoids, *Diglyphus isaea* and *Neochrysocharis formosa* (Hymenoptera: Eulophidae). J. Integr. Agric..

[114] Cheng X.Q., Cao F.Q., Zhang Y.B., Guo J.Y., Wan F.H., Liu W.X. (2017). Life history and life table of the host-feeding parasitoid *Hemiptarsenus varicornis* (Hymenoptera: Eulophidae). Appl. Entomol. Zool..

[115] Xuan J.-L., Liu W.-X., Zhang Y.-B., Cheng X.-Q., Guo J.-Y., Wan F.-H. (2018). Interactions between *Diglyphus isaea* and *Neochrysocharis formosa* (Hymenoptera: Eulophidae), two parasitoids of agromyzid leafminers. Biol. Control..

[116] Osmankhil M.H., Mochizuki A., Hamasaki K., Iwabuchi K. (2010). Oviposition and larval development of *Neochrysocharis formosa* (Hymenoptera: Eulophidae) inside the host larvae, *Liriomyza trifolii*. Jpn. Agric. Res. Q..

[117] Chien C.-C., Chang S.-C., Ku S.-C. (2004). Influence of both population increase and host-killing capability of *Hemiptarsenus varicornis* (Hymenoptera: Eulophidae). Formos. Entomol..

[118] Parrella M.P., Yost J.T., Heinz K.M., Ferrentino G.W. (1989). Mass rearing of *Diglyphus begini* (Hymenoptera, Eulophidae) for biological control of *Liriomyza trifolii* (Diptera, Agromyzidae). J. Econ. Entomol..

[119] Maharjan R., Kwon M., Kim J., Jung C. (2017). Mass production of *Diglyphus isaea* (Hymenoptera: Eulophidae), a biological control agent of a Korean population of potato leaf miner *Liriomyza huidobrensis* (Blanchard) (Diptera: Agromyzidae). Entomol. Res..

[120] Ozawa A., Ota M., Saito T. (2004). Biological control of the American serpentine leafminer, *Liriomyza trifolii* (Burgess), on cherry tomato in greenhouses by the parasitoids, *Hemiptarsenus varicornis* (Girault). Annu. Rep. Kanto-Tosan Plant Prot. Soc..

[121] Ho T.T.G., Ueno T. (2007). Improving parasitoid performance by improving adult food quality: A case study for the leafminer parasitoid *Hemiptarsenus varicornis* (Hymenoptera: Eulophidae). J. Fac. Agric. Kyushu Univ..

[122] Ho T.T.G., Ueno T. (2011). The effects of honey as a dietary supplement on the survivorship and nutrition-storing capacity of *Hemiptarsenus varicornis* (Hymenoptera: Eulophidae), a parasitoid of *Liriomyza* (Diptera: Agromyzidae) leafminers. Int. J. Trop. Insect Sci..

[123] Hondo T., Kandori I., Sugimoto T. (2006). Mass production process of *Neochrysocharis formosa* as the biological control agent against *Liriomyza trifolii*. Mem. Fac. Agric. Kinki Univ..

[124] Saleh A., Allawi T.F., Ghabeish I. (2010). Mass rearing of *Neochrysocharis formosa* (Westwood) (Eulophidae: Hymenoptera), a parasitoid of leafminers (Agromyzidae: Diptera). J. Pest Sci..

[125] Tran D.H., Ueno T., Takagi M. (2007). Comparison of the suitability of *Liriomyza chinensis* and *L. trifolii* (Diptera: Agromyzidae) as hosts for *Neochrysocharis okazakii* (Hymenoptera: Eulophidae). Biol. Control..

[126] Bolckmans K.J.F., Albajes R., Lodovica Gullino M., van Lenteren J.C., Elad Y. (1999). Commercial aspects of biological pest control in greenhouses. Integrated Pest and Disease Management in Greenhouse Crops.

[127] Strand M.R., Pech L.L. (1995). Immunological basis for compatibility in parasitoid host relationships. Annu. Rev. Entomol..

[128] Abe Y., Takeuchi T., Tokumaru S., Kamata J. (2005). Comparison of the suitability of three pest leafminers (Diptera: Agromyzidae) as hosts for the parasitoid *Dacnusa sibirica* (Hymenoptera: Bracenidae). Eur. J. Entomol..

[129] Abe Y. (2017). Invasion of Japan by exotic leafminers *Liriomyza* spp. (Diptera: Agromyzidae) and its consequences. Appl. Entomol. Zool..

[130] Hendrikse A. (1980). A method for mass rearing two braconid parasites (*Dacnusa sibirica* and *Opius pallipes*) of the tomato leafminer (*Liriomyza bryoniae*). Meded. Van De Fac. Landbouwwet. Rijksuniv. Gent.

[131] Petitt F.L. (1993). Biological control in the integrated pest management program at The Land, EPCOT Center. IOBC/WPRS Bull..

[132] Petitt F.L., Wietlisbach D.O. (1994). Laboratory rearing and life-history of *Liriomyza sativae* (Diptera: Agromyzidae) on lima bean. Environ. Entomol..

[133] Petitt F.L., Shuman D.S., Wietlisbach D.O., Coffelt J.A. (1996). An automated system for collection and counting of parasitized leafminer (Diptera: Agromyzidae) larvae. Fla. Entomol..

[134] Helyer N., Cattlin N.D., Brown K.C. (2014). Biological Control in Plant Protection: A Colour Handbook.

[135] Johnson M.W. (1987). Parasitization of *Liriomyza* spp. (Diptera: Agromyzidae) infesting commercial watermelon plantings in Hawaii. J. Econ. Entomol..

[136] Petcharat J., Johnson M.W. (1988). Biology of the leafminer parasitoid *Ganaspidium utilis* Beardsley (Hymenoptera, Eucoilidae). Ann. Entomol. Soc. Am..

[137] Johnson M.W. (1993). Biological control of *Liriomyza* leafminers in the Pacific Basin. Micronesica.

[138] Rathman R.J., Johnson M.W., Tabashnik B.E. (1991). Production of *Ganaspidium utilis* (Hymenoptera: Eucoilidae) for biological control of *Liriomyza* spp. (Diptera: Agromyzidae). Biol. Control..

[139] Costa-Lima T.C., Geremias L.D., Begiato A.M., Chagas M.C.M.d., Parra J.R.P. (2017). Sistema de criação de parasitoide de mosca-minadora. Embrapa Semiárido. Circ. Técnica.

[140] Costa-Lima T.C., Coelho A., Diniz A.J.F., Sampaio M.V., Souza B., Vázquez L.L., Marucci R.C. (2019). Parasitoids Insects. Natural Enemies of Insect Pests in Neotropical Agroecosystems: Biological Control and Functional Biodiversity.

[141] Heinz K.M., Nunney L., Parrella M.P. (1993). Toward predictable biological control of *Liriomyza trifolii* (Diptera: Agromyzidae) infesting greenhouse cut chrysanthemums. Environ. Entomol..

[142] Heinz K.M. (1996). Space- and cohort-dependent longevity in adult *Liriomyza trifolii* (Burgess) (Diptera: Agromyzidae) mass-rearing cultures. Can. Entomol..

[143] Koppert Miglyphus. https://www.koppert.com/miglyphus/.

[144] Colinet H., Boivin G. (2011). Insect parasitoids cold storage: A comprehensive review of factors of variability and consequences. Biol. Control..

[145] Burgio G., Nicoli G. Cold storage of *Diglyphus isaea*. Proceedings of the 7th Workshop of the Global IOBC Working Group “Quality Control of Mass Reared Arthropods”.

[146] Steinberg S., Gouldman D., Kamioslci L. The effect of storage conditions on adult longevity, fertility and sex-ratio of the leafminer parasitoid *Diglyphus isaea* (Walker). Proceedings of the Fifth Workshop of the Global lOBC Working Group, Quality Control of Mass-Reared Organisms.

[147] Chien C.-C., Ku S.-C., Chang S.-C. (2005). Study of the storage and oviposition-regulating capability of *Hemiptarsenus varicornis* (Hymenoptera: Eulophidae). Formos. Entomol..

[148] Leppla J.N.C., van Lenteren J.C. (2003). Need for quality control of mass-produced biological control agents. Quality Control and Production of Biological Control Agents: Theory and Testing Procedures.

[149] Plouvier W.N., Wajnberg E. (2018). Improving the efficiency of augmentative biological control with arthropod natural enemies: A modeling approach. Biol. Control..

[150] Hoffmann A.A., Ross P.A. (2018). Rates and patterns of laboratory adaptation in (mostly) insects. J. Econ. Entomol..

[151] van Lenteren J.C., van Lenteren J.C. (2003). Need for quality control of mass-produced biological control agents. Quality Control and Production of Biological Control Agents: Theory and Testing Procedures.

[152] van Lenteren J.C., Nicoli G., Heinz K.M., Van Driesche R.G., Parrella M.P. (2004). Quality control of mass-produced beneficial insects. Biocontrol in Protected Culture.

[153] Heimpel G.E., Lundgren J.G. (2000). Sex ratios of commercially reared biological control agents. Biol. Control..

[154] Chow A., Heinz K.M. (2005). Using hosts of mixed sizes to reduce male-biased sex ratio in the parasitoid wasp, *Diglyphus isaea*. Entomol. Exp. Appl..

[155] Chow A., Heinz K.M. (2006). Control of *Liriomyza langei* on chrysanthemum by *Diglyphus isaea* produced with a standard or modified parasitoid rearing technique. J. Appl. Entomol..

[156] Zuijderwijk M., van den Hoek C., Mostert J. (2008). Creating crop solutions in chrysanthemums by using the combined strengths of beneficials and chemicals. IOBC/WPRS Bull..

[157] Jacobson R. (2008). Towards a robust IPM programme for organic tomato. IOBC/WPRS Bull..

[158] Lee J. (2004). Kenya revisited. Horticulturist.

[159] Bethke J.A., Paine T.D. (1991). Screen hole size and barriers for exclusion of insect pests of glasshouse crops. J. Entomol. Sci..

[160] Bethke J.A., Redak R.A., Paine T.D. (1994). Screens deny specific pests entry to greenhouses. Calif. Agric..

[161] Murphy G.D., Gates C., Watson G.R. (2011). An update on the use of biological control in greenhouse ornamental crops in Canada. IOBC/WPRS Bull..

[162] Online Zoological Collections of Australian Museums. https://ozcam.ala.org.au/occurrences/1e256e99-f916-4fa5-a206-8df41ba4f75c.

[163] Bjorksten T.A., Robinson M. (2005). Juvenile and sublethal effects of selected pesticides on the leafminer parasitoids *Hemiptarsenus varicornis* and *Diglyphus isaea* (Hymenoptera: Eulophidae) from Australia. J. Econ. Entomol..

[164] Baker D.A., Loxdale H.D., Edwards O.R. (2003). Genetic variation and founder effects in the parasitoid wasp, *Diaeretiella rapae* (M’intosh) (Hymenoptera: Braconidae: Aphidiidae), affecting its potential as a biological control agent. Mol. Ecol..

[165] Lommen S.T.E., de Jong P.W., Pannebakker B.A. (2017). It is time to bridge the gap between exploring and exploiting: Prospects for utilizing intraspecific genetic variation to optimize arthropods for augmentative pest control—A review. Entomol. Exp. Appl..

[166] Waage J.K., Carl K.P., Mills N.J., Greathead D.J., Singh P., Moore R.F. (1985). Rearing entomophagous insects. Handbook of Insect Rearing.

[167] Aeschlimann J.-P. (1990). Simultaneous occurrence of thelytoky and bisexuality in hymenopteran species, and its implications for the biological control of pests. Entomophaga.

[168] Stouthamer R. (1993). The use of sexual versus asexual wasps in biological control. Entomophaga.

[169] Stouthamer R., van Lenteren J.C. (2003). The use of unisexual wasps in biological control. Quality Control and Production of Biological Control Agents: Theory and Testing Procedures.

[170] Ramirez-Romero R., Sivinski J., Copeland C.S., Aluja M. (2012). Are individuals from thelytokous and arrhenotokous populations equally adept as biocontrol agents? Orientation and host searching behavior of a fruit fly parasitoid. Biocontrol.

[171] Sivinski J. (2013). Augmentative biological control: Research and methods to help make it work. CAB Rev..

[172] Hagimori T., Abe Y., Date S., Miura K. (2006). The first finding of a *Rickettsia* bacterium associated with parthenogenesis induction among insects. Curr. Microbiol..

[173] Adachi-Hagimori T., Miura K., Stouthamer R. (2008). A new cytogenetic mechanism for bacterial endosymbiont-induced parthenogenesis in Hymenoptera. Proc. R. Soc. B-Biol. Sci..

[174] Adachi-Hagimori T., Miura K. (2008). Development of a multiplex method to discriminate between *Neochrysocharis formosa* (Hymenoptera: Eulophidae) reproductive modes. J. Econ. Entomol..

[175] Adachi-Hagimori T., Miura K., Abe Y. (2011). Gene flow between sexual and asexual strains of parasitic wasps: A possible case of sympatric speciation caused by a parthenogenesis-inducing bacterium. J. Evol. Biol..

[176] Yang Y.-M., Xuan J.-L., Ye F.-Y., Guo J.-Y., Yang L.-Y., Wan-Xue L. (2017). Molecular identification of the thelytokous strain of *Neochrysocharis formosa* (Hymenoptera: Eulophidae) newly found in China and detection of its endosymbiont *Rickettsia*. Acta Entomol. Sin..

[177] Ye F., Yang Y., Zhang Y., Pan L., Yefremova Z., Yang L., Guo J., Liu W. (2023). The thelytokous strain of the parasitoid *Neochrysocharis formosa* outperforms the arrhenotokous strain in reproductive capacity and biological control of agromyzid leafminers. Pest Manag. Sci..

[178] Gebiola M., Mauck K.E., Hunter M.S., Heraty J.M., Woolley J.B. (2025). The smallest in the small: Symbionts in Chalcidoidea. Chalcidoidea of the World.

[179] Burks R.A., Gumovsky A.V., Hansson C., Gates M.W., Domer T., Perry R.K., Heraty J.M., Woolley J.B. (2025). Eulophidae. Chalcidoidea of the World.

[180] Ohata Y., Tagami Y. (2025). Antibiotic agrochemical treatment reduces endosymbiont infections and alters population dynamics in leafminers, thrips, and parasitoid wasps. Front. Microbiol..

[181] Yamashita S., Takahashi K.H. (2018). Artificial transfer of a thelytoky-inducing *Wolbachia* endosymbiont between strains of the endoparasitoid wasp *Asobara japonica* (Hymenoptera: Braconidae). Appl. Entomol. Zool..

[182] Vorburger C. (2014). Thelytoky and sex determination in the Hymenoptera: Mutual constraints. Sex. Dev..

[183] Aeschlimann J.P. (1986). Distribution and effectiveness of *Anaphes diana* [=*Patasson lameereiI*] [Hym: Mymaridae], a parasitoid of *Sitona* spp. eggs [Col: Curculionidae] in the Mediterranean region. Entomophaga.

[184] Ye F.-Y., Zhu C.-D., Yefremova Z., Liu W.-X., Guo J.-Y., Wan F.-H. (2018). Life history and biocontrol potential of the first female-producing parthenogenetic species of *Diglyphus* (Hymenoptera: Eulophidae) against agromyzid leafminers. Sci. Rep..

[185] Du S.J., Yefremova Z., Ye F.Y., Zhu C.D., Guo J.Y., Liu W.X. (2021). Morphological and molecular identification of arrhenotokous strain of *Diglyphus wani* (Hymenoptera, Eulophidae) found in China as a control agent against agromyzid leafminers. ZooKeys.

[186] Tilquin A., Kokko H. (2016). What does the geography of parthenogenesis teach us about sex?. Philos. Trans. R. Soc. B: Biol. Sci..

[187] Du S.J., Ye F.Y., Xu S.Y., Wan W.J., Guo J.Y., Yan N.W., Liu W.X. (2023). Thelytokous *Diglyphus wani*: A more promising biological control agent against agromyzid leafminers than its arrhenotokous counterpart. J. Integr. Agric..

[188] Du S.J., Ye F.Y., Wang Q.J., Liang Y.X., Wan W.J., Guo J.Y., Liu W.X. (2022). Multiple data demonstrate that bacteria regulating reproduction could be not the cause for the thelytoky of *Diglyphus wani* (Hymenoptera: Eulophidae). Insects.

[189] Du S.J., Ye F.Y., Xu S.Y., Liang Y.X., Wan F.H., Guo J.Y., Liu W.X. (2023). Apomixis for no bacteria-induced thelytoky in *Diglyphus wani* (Hymenoptera: Eulophidae). Front. Genet..

[190] Leung K., Ras E., Ferguson K.B., Ariëns S., Babendreier D., Bijma P., Bourtzis K., Brodeur J., Bruins M.A., Centurión A. (2020). Next-generation biological control: The need for integrating genetics and genomics. Biol. Rev..

