# Supplementary material for: Leveraging Adventive and Endemic Parasitoids Against Polyphagous Agromyzid Leafminers in Australia
Source: Insects. 2025 Sep 16;16(9):968. doi: 10.3390/insects16090968 (PMC12471177; doi:10.3390/insects16090968)
Supplement: Supplementary file 1 [file insects-16-00968-s001.zip › insects-3861004-supplementary.pdf]

Leveraging adventive and endemic parasitoids against polyphagous agromyzid leafminers in Australia

Ridland, P.M.; Pirtle, E.I.; Umina, P.A.; Hoffmann A.A.

Supplementary Information

Table S1	Parasitoid species recorded from *Liriomyza trifolii*
Table S2	Parasitoid species recorded from *Liriomyza huidobrensis*
Table S3	Parasitoid species recorded from *Liriomyza sativae*
Table S4	Overlap of parasitoid species recorded from the three *Liriomyza* species
Table S5	Parasitoid species by location records in descending order (pooling all records for a particular location for each *Liriomyza* species)

Table S1 Parasitoid species reared from *Liriomyza trifolii*

Specimens reared from mixed collections of *Liriomyza* spp., which included *L. trifolii*, were not included in the list. As with any compilation of species names from the scientific literature, there will be uncertainty about the validity of some species and sampling intensity varies dramatically between countries, which usually reflects the scientific input into the problem rather than the actual size of the problem (Grenouillet *et al.* 1993; Noyes 1994; Shaw 1994). Given the nomenclatural changes that have occurred in time, the currently accepted name is given, together with the name used in the original reference. All references for the records are included.

**Family**	**Sub-family**	**Parasitoid (current)**	**Parasitoid (in reference)**	**Location**	**Reference**
Braconidae	Alysiinae	*Dacnusa nipponica* Takada, 1977	*Dacnusa nipponica*	Japan	Saito *et al.* (1996); Cai & Saito (2011)
Braconidae	Alysiinae	*Dacnusa sasakawai* Takada, 1977	*Dacnusa sasakawai*	Japan	Amano *et al.* (2008); Cai & Saito (2011)
Braconidae	Alysiinae	*Pseudopezomachus masii* Nixon, 1940	*Pseudopezomachus masii*	Egypt	Hegazi *et al.* (2014); Neama & Hegazi (2014)
Braconidae	Opiinae	*Opius ambiguus* Wesmael, 1835	*Opius ambiguus*	Turkey	Çıkman & Uygun (2003)
Braconidae	Opiinae	*Opius basalis* Fischer, 1958	*Opius basalis*	Turkey	Çikman (2006); Çikman et al. (2006); Neama & Hegazi (2014); Ali et al. (2020)
Braconidae	Opiinae	*Opius bruneipes* Gahan, 1913	*Opius bruneipes*	USA	Neuenschwander *et al.* (1987); Schuster *et al.* (1991); Schuster & Wharton (1993); Hernández *et al.* (2009)
Braconidae	Opiinae	*Opius dimidiatus* (Ashmead, 1889)	*Opius dimidatus*	China	Liu *et al.* (2012); Wang *et al.* (2017)
Braconidae	Opiinae	*Opius dimidiatus* (Ashmead, 1889)	*Opius dimidiatus*	Mexico	Valenzuela-Escoboza *et al.* (2010)
Braconidae	Opiinae	*Opius dimidiatus* (Ashmead, 1889)	*Opius dimidiatus*	Senegal	Neuenschwander *et al.* (1987)
Braconidae	Opiinae	*Opius dimidiatus* (Ashmead, 1889)	*Opius dimidiatus*	USA	Stegmaier (1972); Schuster *et al.* (1991); Schuster & Wharton (1993)
Braconidae	Opiinae	*Opius dissitus* Muesebeck, 1963	*Opius dissitus*	Egypt	Bassiony *et al.* (2017)
Braconidae	Opiinae	*Opius dissitus* Muesebeck, 1963	*Opius dissitus*	Indonesia	Yasa *et al.* (2020)
Braconidae	Opiinae	*Opius dissitus* Muesebeck, 1963	*Opius dissitus*	Mexico	Holguín-Peña *et al.* (2019)
Braconidae	Opiinae	*Opius dissitus* Muesebeck, 1963	*Opius dissitus*	Mexico	Valenzuela-Escoboza *et al.* (2010)
Braconidae	Opiinae	*Opius dissitus* Muesebeck, 1963	*Opius dissitus*	Réunion	Olivera & Bordat (1996)
Braconidae	Opiinae	*Opius dissitus* Muesebeck, 1963	*Opius dissitus*	USA	Neuenschwander *et al.* (1987); Schuster *et al.* (1991); Schuster & Wharton (1993); Hernández *et al.* (2009); Li *et al.* (2012)
Braconidae	Opiinae	*Opius exigua* Wesmael, 1835	*Opius exiguus*	India	Sharma & Kumar (2017)
Braconidae	Opiinae	*Opius exigua* Wesmael, 1835	*Opius exiguus*	Turkey	Çıkman & Uygun (2003)
Braconidae	Opiinae	*Opius gafsaensis* Fischer, 1964	*Opius gafsaensis*	Turkey	Çıkman & Uygun (2003)

Table S1 Parasitoid species reared from *Liriomyza trifolii*

Family	Sub-family	Parasitoid (current)	Parasitoid (in reference)	Location	Reference
Braconidae	Opiinae	*Opius* nr *brownsvillensis*	*Opius nr brownsvillensis*	USA	Hernández *et al.* (2009)
Braconidae	Opiinae	*Opius osogovoensis* Fischer, 1964	*Opius osogovoensis*	Turkey	Çıkman & Uygun (2003)
Braconidae	Opiinae	*Opius pallipes* Wesmael, 1835	*Opius pallipes*	Egypt	Bayoumy *et al.* (2018)
Braconidae	Opiinae	*Opius pallipes* Wesmael, 1835	*Opius pallipes*	Turkey	Çıkman & Uygun (2003)
Braconidae	Opiinae	*Opius* sp.	*Opius* sp.	Egypt	Ali *et al.* (2020)
Braconidae	Opiinae	*Opius* sp.	*Opius* sp.	Japan	Amano *et al.* (2008)
Braconidae	Opiinae	*Opius* sp.	*Opius* sp.	Spain	Cabello *et al.* (1994)
Braconidae	Opiinae	*Opius* sp.	*Opius* sp.	Taiwan	Lin & Wang (1992); Chien & Ku (1998)
Braconidae	Opiinae	*Opius* sp.	*Opius* sp.	Venezuela	Chirinos *et al.* (2014)
Braconidae	Opiinae	*Opius* sp. *1*	*Opius* sp. *1*	USA	Hernández *et al.* (2009)
Braconidae	Opiinae	*Opius* sp. *2*	*Opius* sp. *2*	USA	Hernández *et al.* (2009)
Braconidae	Opiinae	*Opius* spp.	*Opius* sp.	USA	Schuster & Price (1985); Hernández *et al.* (2009)
Braconidae	Opiinae	*Opius thoracosema* sp. *3*	*Opius thoracosema* sp. *3*	USA	Hernández *et al.* (2009)
Braconidae	Opiinae	*Opius (Phaedrotoma) biroi* Fischer, 1960	*Opius biroi*	China	Xing *et al.* (2017)
Braconidae	Tersilochinae	*Allophrys* sp.	*Allophrys* sp.	Senegal	Neuenschwander *et al.* (1987)
Eulophidae	Entedoninae	*Apleurotropis kumatai* (Kamijo, 1977)	*Apleurotropis kumatai*	Japan	Cai & Saito (2011)
Eulophidae	Entedoninae	*Asecodes delucchii* (Bouček, 1971)	*Asecodes delucchii*	India	Sharma & Kumar (2017)
Eulophidae	Entedoninae	*Asecodes delucchii* (Bouček, 1971)	*Asecodes delucchii*	Japan	Arakaki & Kinjo (1998)
Eulophidae	Entedoninae	*Asecodes erxias* (Walker, 1848)	*Asecodes erxias*	India	Sharma & Kumar (2017)
Eulophidae	Entedoninae	*Asecodes erxias* (Walker, 1848)	*Asecodes erxias; Teleopterus erxias*	Japan	Saito *et al.* (1996); Nishino & Uchida (1999)
Eulophidae	Entedoninae	*Asecodes* sp.	*Asecodes* sp.	USA	Hernández *et al.* (2009)
Eulophidae	Entedoninae	*Chrysocharis ainsliei* Crawford, 1912	*Chrysocharis ainsliei*	USA	Chandler (1982); Trumble & Nakakihara (1983)
Eulophidae	Entedoninae	*Chrysocharis caribea* Bouček, 1977	*Chrysocharis caribae*	Trinidad	Neuenschwander *et al.* (1987)
Eulophidae	Entedoninae	*Chrysocharis caribea* Bouček, 1977	*Chrysocharis caribae*	USA	Neuenschwander *et al.* (1987)
Eulophidae	Entedoninae	*Chrysocharis liriomyzae* Delucchi, 1954	*Chrysocharis liriomyzae*	Turkey	Çıkman & Uygun (2003)
Eulophidae	Entedoninae	*Chrysocharis orbicularis* (Nees, 1834)	*Chrysocharis orbicularis*	Italy	Rizzo & Massa (2002)
Eulophidae	Entedoninae	*Chrysocharis oscinidis* Ashmead, 1888	*Chrysocharis oscinidis*	China	Liu *et al.* (2012)

Table S1 Parasitoid species reared from *Liriomyza trifolii*

Family	Sub-family	Parasitoid (current)	Parasitoid (in reference)	Location	Reference
Eulophidae	Entedoninae	*Chrysocharis oscinidis* Ashmead, 1888	*Chrysocharis parksi*	Mexico	Valenzuela-Escoboza *et al.* (2010)
Eulophidae	Entedoninae	*Chrysocharis oscinidis* Ashmead, 1888	*Chrysocharis oscinidis*	Tonga	Johnson (1993)
Eulophidae	Entedoninae	*Chrysocharis oscinidis* Ashmead, 1888	*Chrysocharis parksi*	USA	Parrella et al. (1982); Trumble & Nakakihara (1983); Schuster & Price (1985; Neuenschwander et al. (1987); Schuster et al. (1991);
Eulophidae	Entedoninae	*Chrysocharis pentheus* (Walker, 1839)	*Chrysocharis pentheus*	China	Chen *et al.* (2006); Liu *et al.* (2012); Wang *et al.* (2017)
Eulophidae	Entedoninae	*Chrysocharis pentheus* (Walker, 1839)	*Chrysocharis pentheus*	Egypt	Neama & Hegazi (2014); Ali *et al.* (2020)
Eulophidae	Entedoninae	*Chrysocharis pentheus* (Walker, 1839)	*Chrysocharis pentheus*	Japan	Nishino *et al.* (1997); Arakaki & Kinjo (1998); Nishino & Uchida (1999); Amano *et al.* (2008); Saito *et al.* (2008); Cai & Saito (2011)
Eulophidae	Entedoninae	*Chrysocharis pentheus* (Walker, 1839)	*Chrysocharis pentheus*	Taiwan	Lin & Wang (1992); Chien & Ku (1998)
Eulophidae	Entedoninae	*Chrysocharis pentheus* (Walker, 1839)	*Chrysocharis pentheus*	Turkey	Civelek & La Salle (2005)
Eulophidae	Entedoninae	*Chrysocharis pubicornis* (Zetterstedt, 1838)	*Chrysocharis pubicornis*	Japan	Cai & Saito (2011)
Eulophidae	Entedoninae	*Chrysocharis* sp.	*Chrysocharis* sp.	Egypt	Abul Fadl & El-Khawas (2009)
Eulophidae	Entedoninae	*Chrysocharis* sp.	*Chrysocharis* sp.	Israel	Freidberg & Gijswijtz (1983)
Eulophidae	Entedoninae	*Chrysocharis* sp.	*Chrysocharis* sp.	Mexico	Holguín-Peña *et al.* (2019)
Eulophidae	Entedoninae	*Chrysocharis* sp.	*Chrysocharis* sp.	USA	Hernández *et al.* (2009)
Eulophidae	Entedoninae	*Chrysonotomyia ricini* Parshuram & Agnihotri, 2017	*Chrysonotomyia ricini*	India	Murugan & Dhandapani (2006); Parshuram & Agnihotri (2017)
Eulophidae	Entedoninae	*Chrysonotomyia smaragdulus* (Graham, 1963)	*Chrysonotomyia smaragdula*	Turkey	Çıkman & Uygun (2003)
Eulophidae	Entedoninae	*Chrysonotomyia* sp.	*Chrysonotomyia* sp.	Israel	Freidberg & Gijswijtz (1983)
Eulophidae	Entedoninae	*Chrysonotomyia* sp.	*Chrysonotomyia* sp.	USA	Chandler (1982)
Eulophidae	Entedoninae	*Chrysonotomyia* sp. near *leptocera*	*Chrysonotomyia* sp. near *leptocera*	Trinidad	Neuenschwander *et al.* (1987)
Eulophidae	Entedoninae	*Chrysonotomyia* sp. *F*	*Chrysonotomyia* sp. *F*	Senegal	Neuenschwander *et al.* (1987)
Eulophidae	Entedoninae	*Cirrospilus* sp. *near cinctiventris*	*Cirrospilus* sp. *near cinctiventris*	Trinidad	Neuenschwander *et al.* (1987)
Eulophidae	Entedoninae	*Closterocerus agromyzae* Narayanan, Subba Rao and Ramachandra, 1960	*Closterocerus agromyzae*	India	Reji *et al.* (2003)

Table S1 Parasitoid species reared from *Liriomyza trifolii*

Family	Sub-family	Parasitoid (current)	Parasitoid (in reference)	Location	Reference
Eulophidae	Entedoninae	*Closterocerus cinctipennis* Ashmead, 1888	*Closterocerus cinctipennis*	Mexico	Valenzuela-Escoboza *et al.* (2010); Holguín-Peña *et al.* (2019)
Eulophidae	Entedoninae	*Closterocerus cinctipennis* Ashmead, 1888	*Closterocerus cinctipennis*	USA	Stegmaier (1966); Hernández *et al.* (2009)
Eulophidae	Entedoninae	*Closterocerus cinctipennis* Ashmead, 1888	*Closterocerus cintipennis*	Venezuela	Chirinos *et al.* (2014)
Eulophidae	Entedoninae	*Closterocerus purpureus* (Howard, 1897)	*Closterocerus purpureus*	Trinidad	Neuenschwander *et al.* (1987)
Eulophidae	Entedoninae	*Closterocerus utahensis* Crawford, 1912	*Closterocerus utahensis*	USA	Palumbo *et al.* (1994)
Eulophidae	Entedoninae	*Neochrysocharis agromyzae* (Crawford, 1913)	*Derostenus agromyzae*	USA	Stegmaier (1966)
Eulophidae	Entedoninae	*Neochrysocharis ambitiosa* Hansson, 1990	*Neochrysocharis ambitiosa*	Turkey	Çıkman & Uygun (2003)
Eulophidae	Entedoninae	*Neochrysocharis arvensis* Graham, 1963	*Neochrysocharis arvensis*	Turkey	Gençer (2004)
Eulophidae	Entedoninae	*Neochrysocharis chlorogaster* (Erdös, 1966)	*Neochrysocharis chlorogaster*	Turkey	Ulubilir & Yabas (2000)
Eulophidae	Entedoninae	*Neochrysocharis diastatae* (Howard, 1881)	*Neochrysocharis punctiventris*	China	Wang *et al.* (2017)
Eulophidae	Entedoninae	*Neochrysocharis diastatae* (Howard, 1881)	*Chrysonotomyia punctiventris; Neochrysocharis punctiventris*	USA	Trumble & Nakakihara (1983); Schuster & Price (1985); Lynch & Johnson (1987); Neuenschwander *et al.* (1987); Schuster *et al.* (1991); Schuster & Wharton (1993)
Eulophidae	Entedoninae	*Neochrysocharis formosa* (Walker, 1833)	*Neochrysocharis formosa*	Turkey	Çıkman & Uygun (2003)
Eulophidae	Entedoninae	*Neochrysocharis formosa* (Westwood, 1833)	*Chrysonotomyia formosa*	China	Liu *et al.* (2012); Wang *et al.* (2017)
Eulophidae	Entedoninae	*Neochrysocharis formosa* (Westwood, 1833)	*Neochrysocharis formosa*	Egypt	Ali *et al.* (2020)
Eulophidae	Entedoninae	*Neochrysocharis formosa* (Westwood, 1833)	*Chrysonotomyia formosa*	Guam	Schreiner *et al.* (1986)

Table S1 Parasitoid species reared from *Liriomyza trifolii*

Family	Sub-family	Parasitoid (current)	Parasitoid (in reference)	Location	Reference
Eulophidae	Entedoninae	*Neochrysocharis formosa* (Westwood, 1833)	*Neochrysocharis formosa*	India	Sharma & Kumar (2017)
Eulophidae	Entedoninae	*Neochrysocharis formosa* (Westwood, 1833)	*Neochrysocharis formosa*	Indonesia	Yasa *et al.* (2020)
Eulophidae	Entedoninae	*Neochrysocharis formosa* (Westwood, 1833)	*Closterocerus formosus*	Iran	Talebi *et al.* (2005); Asadi *et al.* (2006); Dousti *et al.* (2008); Shahreki *et al.* (2016)
Eulophidae	Entedoninae	*Neochrysocharis formosa* (Westwood, 1833)	*Chysonotomyia formosa*; *Neochrysocharis formosa*	Italy	Del Bene (1989); Rizzo & Massa (2002)
Eulophidae	Entedoninae	*Neochrysocharis formosa* (Westwood, 1833)	*Neochrysocharis formosa*	Japan	Saito *et al.* (1996); Arakaki & Kinjo (1998); Nishino & Uchida (1999); Amano *et al.* (2008); Saito *et al.* (2008); Cai & Saito (2011)
Eulophidae	Entedoninae	*Neochrysocharis formosa* (Westwood, 1833)	*Neochrysocharis formosa*	Jordan	Al-Ghabeish & Allawi (2001)
Eulophidae	Entedoninae	*Neochrysocharis formosa* (Westwood, 1833)	*Neochrysocharis formosa*	Philippines	Baucas *et al.* (2003); Arida *et al.* (2013)
Eulophidae	Entedoninae	*Neochrysocharis formosa* (Westwood, 1833)	*Chrysonotomyia* sp., *formosa group*	Senegal	Neuenschwander *et al.* (1987)
Eulophidae	Entedoninae	*Neochrysocharis formosa* (Westwood, 1833)	*Chrysonotomyia formosa*	Spain	Cabello *et al.* (1994)
Eulophidae	Entedoninae	*Neochrysocharis formosa* (Westwood, 1833)	*Chrysonotomyia* sp.	Taiwan	Lin & Wang (1992); Chien & Ku (1998)
Eulophidae	Entedoninae	*Neochrysocharis formosa* (Westwood, 1833)	*Chrysonotomyia formosa*; *Closterocerus formosus*; *Neochrysocharis formosa*	Turkey	Ulubilir & Yabas (2000); Gençer (2004); Çikman *et al.* (2006); Çikman & Çömlekçioğlu (2006); Çikman (2012)
Eulophidae	Entedoninae	*Neochrysocharis formosa* (Westwood, 1833)	*Derostenus variipes*; *Neochrysocharis formosa*	USA	Stegmaier (1966); Hernández *et al.* (2009)

Table S1 Parasitoid species reared from *Liriomyza trifolii*

Family	Sub-family	Parasitoid (current)	Parasitoid (in reference)	Location	Reference
Eulophidae	Entedoninae	*Neochrysocharis formosa* (Westwood, 1833)	*Neochrysocharis formosa*	Venezuela	Chirinos *et al.* (2014)
Eulophidae	Entedoninae	*Neochrysocharis indicus* Khan, Agnihotri and Sushil, 2005	*Closterocerocus indicus*; *Closterocerus indica*; *Chrysocharis indicus*	India	Bhat *et al.* (2009); Gupta & Poorani (2009); Sharma & Kumar (2017)
Eulophidae	Entedoninae	*Neochrysocharis okazakii* Kamijo, 1978	*Neochrysocharis okazakii*; *Chysonotomyia okazakii*	China	Chen *et al.* (2006); Liu *et al.* (2012)
Eulophidae	Entedoninae	*Neochrysocharis okazakii* Kamijo, 1978	*Neochrysocharis okazakii*	Indonesia	Yasa *et al.* (2020)
Eulophidae	Entedoninae	*Neochrysocharis okazakii* Kamijo, 1978	*Neochrysocharis okazakii*	Japan	Saito *et al.* (1996); Arakaki & Kinjo (1998); Nishino & Uchida (1999); Saito *et al.* (2008); Cai & Saito (2011)
Eulophidae	Entedoninae	*Neochrysocharis okazakii* Kamijo, 1978	*Neochrysocharis okazakii*	Philippines	Baucas *et al.* (2003); Arida *et al.* (2013)
Eulophidae	Entedoninae	*Neochrysocharis okazakii* Kamijo, 1978	*Chrysonotomyia okazakii*	Taiwan	Lin & Wang (1992); Chien & Ku (1998)
Eulophidae	Entedoninae	*Neochrysocharis pictipes* (Crawford, 1912)	*Neochrysocharis albipes*	Turkey	Çıkman & Uygun (2003)
Eulophidae	Entedoninae	*Neochrysocharis sericea* (Erdös, 1954)	*Neochrysocharis sericea*	Turkey	Çıkman & Uygun (2003)
Eulophidae	Entedoninae	*Neochrysocharis* sp.	*Neochrysocharis* sp.	Egypt	Abul Fadl & El-Khawas (2009); Ali *et al.* (2020)
Eulophidae	Entedoninae	*Neochrysocharis* sp.	*Neochrysocharis* sp.	Japan	Amano *et al.* (2008); Cai & Saito (2011)
Eulophidae	Entedoninae	*Neochrysocharis* sp.	*Neochrysocharis* sp.	Mexico	Valenzuela-Escoboza *et al.* (2010)
Eulophidae	Entedoninae	*Neochrysocharis* sp.	*Neochrysocharis* sp.	USA	Patel & Schuster (1992)
Eulophidae	Entedoninae	*Pediobius metallicus* (Nees, 1834)	*Pediobius metallicus*	Turkey	Çikman (2006)
Eulophidae	Entedoninae	*Pediobius metallicus* (Nees, 1834)	*Pediobius acantha*	Turkey	Çıkman & Uygun (2003)
Eulophidae	Entedoninae	*Pediobius* sp.	*Pediobius* sp.	Japan	Arakaki & Kinjo (1998)
Eulophidae	Eulophinae	*Burkseus vittatus* (Walker, 1838)	*Cirrospilus vittatus*	Iran	Talebi *et al.* (2005); Asadi *et al.* (2006)
Eulophidae	Eulophinae	*Burkseus vittatus* (Walker, 1838)	*Cirrospilus vittatus*	Italy	Del Bene (1989)
Eulophidae	Eulophinae	*Burkseus vittatus* (Walker, 1838)	*Cirrospilus vittatus*	Spain	Cabello *et al.* (1994)
Eulophidae	Eulophinae	*Burkseus vittatus* (Walker, 1838)	*Cirrospilus vitatus*	Turkey	Çikman (2006); Çikman & Çömlekçioğlu (2006)

Table S1 Parasitoid species reared from *Liriomyza trifolii*

Family	Sub-family	Parasitoid (current)	Parasitoid (in reference)	Location	Reference
Eulophidae	Eulophinae	*Cirrospilus brevicorpus* Shafee & Rizvi, 1988	*Cirrospilus ambiguus*	Taiwan	Hansson & LaSalle (1996); Chien & Ku (1998)
Eulophidae	Eulophinae	*Cirrospilus* sp.	*Cirrospilus* sp.	Egypt	Abul Fadl & El-Khawas (2009)
Eulophidae	Eulophinae	*Cirrospilus* sp. *near cinctiventris*	*Cirrospilus* sp. *near cinctiventris*	Senegal	Neuenschwander *et al.* (1987)
Eulophidae	Eulophinae	*Cirrospilus variegatus group*	*Cirrospilus variegatus group*	USA	Hernández *et al.* (2009)
Eulophidae	Eulophinae	*Diaulinopsis arenaria* (Erdös, 1951)	*Diaulinopsis arenaria*	Iran	Dousti *et al.* (2008)
Eulophidae	Eulophinae	*Diaulinopsis arenaria* (Erdös, 1951)	*Diaulinopsis arenaria*	Jordan	Al-Ghabeish & Allawi (2001)
Eulophidae	Eulophinae	*Diaulinopsis callichroma* Crawford, 1912	*Diaulinopsis callichroma*	Trinidad	Neuenschwander *et al.* (1987)
Eulophidae	Eulophinae	*Diaulinopsis callichroma* Crawford, 1912	*Diaulinopsis callichroma*	USA	Schuster & Price (1985); Schuster *et al.* (1991); Schuster & Wharton (1993)
Eulophidae	Eulophinae	*Diglyphus albiscapus* Erdos, 1951	*Diglyphus albiscapus*	China	Zhu *et al.* (2000)
Eulophidae	Eulophinae	*Diglyphus albiscapus* Erdös, 1951	*Diglyphus albiscapus*	Japan	Saito *et al.* (1996); Arakaki & Kinjo (1998); Nishino & Uchida (1999); Amano *et al.* (2008); Saito *et al.* (2008); Cai & Saito (2011)
Eulophidae	Eulophinae	*Diglyphus begini* (Ashmead, 1904)	*Diglyphus begini*	Colombia	Neuenschwander *et al.* (1987)
Eulophidae	Eulophinae	*Diglyphus begini* (Ashmead, 1904)	*Diglyphus begini*	USA	Parrella *et al.* (1982); Trumble & Nakakihara (1983); Hara (1986); Neuenschwander *et al.* (1987); Heinz & Parrella (1990); Schuster *et al.* (1991); Schuster & Wharton (1993)
Eulophidae	Eulophinae	*Diglyphus chabrias* (Walker, 1838)	*Diglyphus chabrias*	Spain	Cabello *et al.* (1994)
Eulophidae	Eulophinae	*Diglyphus chabrias* (Walker, 1838)	*Diglyphus chabrias*	Turkey	Gençer (2004)
Eulophidae	Eulophinae	*Diglyphus crassinervis* Erdös, 1958	*Diglyphus crassinervis*	Egypt	Abul Fadl & El-Khawas (2009)
Eulophidae	Eulophinae	*Diglyphus crassinervis* Erdös, 1958	*Diglyphus crassinervis*	Iran	Talebi *et al.* (2005); Asadi *et al.* (2006); Dousti *et al.* (2008)
Eulophidae	Eulophinae	*Diglyphus crassinervis* Erdös, 1958	*Diglyphus crassinervis*	Israel	Freidberg & Gijswijtz (1983)
Eulophidae	Eulophinae	*Diglyphus crassinervis* Erdös, 1958	*Diglyphus crassinervis*	Italy	Hansson & Navone (2017)
Eulophidae	Eulophinae	*Diglyphus crassinervis* Erdös, 1958	*Diglyphus crassinervis*	Jordan	Al-Ghabeish & Allawi (2001)
Eulophidae	Eulophinae	*Diglyphus crassinervis* Erdös, 1958	*Diglyphus crassinervis*	Turkey	Çikman *et al.* (2006)

Table S1 Parasitoid species reared from *Liriomyza trifolii*

Family	Sub-family	Parasitoid (current)	Parasitoid (in reference)	Location	Reference
Eulophidae	Eulophinae	*Diglyphus horticola* Khan, 1985	*Diglyphus horticola*	India	Sharma & Kumar (2017)
Eulophidae	Eulophinae	*Diglyphus intermedius* (Girault, 1916)	*Diglyphus intermedius*	Senegal	Neuenschwander *et al.* (1987)
Eulophidae	Eulophinae	*Diglyphus intermedius* (Girault, 1916)	*Diglyphus intermedius*	USA	Stegmaier (1972); Trumble & Nakakihara (1983); Schuster & Price (1985); Hara (1986); Neuenschwander *et al.* (1987); Schuster *et al.* (1991); Patel & Schuster (1992); Schuster & Wharton (1993)
Eulophidae	Eulophinae	*Diglyphus isaea* (Walker, 1838)	*Diglyphus isaea*	China	Chen *et al.* (2006)
Eulophidae	Eulophinae	*Diglyphus isaea* (Walker, 1838)	*Diglyphus isaea*	Egypt	Abul Fadl & El-Khawas (2009); Neama & Hegazi (2014); Bassiony *et al.* (2017); Bayoumy *et al.* (2018); Ali *et al.* (2020)
Eulophidae	Eulophinae	*Diglyphus isaea* (Walker, 1838)	*Diglyphus isaea*	India	Sharma & Kumar (2017)
Eulophidae	Eulophinae	*Diglyphus isaea* (Walker, 1838)	*Diglyphus isaea*	Iran	Talebi *et al.* (2005); Asadi *et al.* (2006); Dousti *et al.* (2008)
Eulophidae	Eulophinae	*Diglyphus isaea* (Walker, 1838)	*Diglyphus isaea*	Israel	Freidberg & Gijswijtz (1983)
Eulophidae	Eulophinae	*Diglyphus isaea* (Walker, 1838)	*Diglyphus isaea*	Italy	Del Bene (1989); Rizzo & Massa (2002); Hansson & Navone (2017)
Eulophidae	Eulophinae	*Diglyphus isaea* (Walker, 1838)	*Diglyphus isaea*	Japan	Saito *et al.* (1996); Nishino & Uchida (1999); Amano *et al.* (2008); Saito *et al.* (2008); Cai & Saito (2011)
Eulophidae	Eulophinae	*Diglyphus isaea* (Walker, 1838)	*Diglyphus isaea*	Philippines	Baucas *et al.* (2003); Arida *et al.* (2013)
Eulophidae	Eulophinae	*Diglyphus isaea* (Walker, 1838)	*Diglyphus isaea*	Senegal	Neuenschwander *et al.* (1987)
Eulophidae	Eulophinae	*Diglyphus isaea* (Walker, 1838)	*Diglyphus isaea*	Spain	Cabello *et al.* (1994)
Eulophidae	Eulophinae	*Diglyphus isaea* (Walker, 1838)	*Diglyphus isaea*	Turkey	Ulubilir & Yabas (2000); Çıkman & Uygun (2003); Gençer (2004); Çikman (2006); Çikman *et al.* (2006); Çikman & Çömlekçioğlu (2006); Gençer (2009); Çikman & La Salle (2011); Çikman (2012)
Eulophidae	Eulophinae	*Diglyphus isaea* (Walker, 1838)	*Diglyphus isaea*	USA	Parrella *et al.* (1982); Hara (1986); Hernández *et al.* (2009)
Eulophidae	Eulophinae	*Diglyphus minoeus* (Walker, 1838)	*Diglyphus minoeus*	Japan	Saito *et al.* (1996); Cai & Saito (2011)
Eulophidae	Eulophinae	*Diglyphus minoeus* (Walker, 1838)	*Diglyphus minoeus*	Turkey	Çıkman & Uygun (2003); Çikman (2006); Çikman & Çömlekçioğlu (2006); Çikman (2012)
Eulophidae	Eulophinae	*Diglyphus poppoea* Walker, 1848	*Diglyphus poppoea*	Portugal	Godinho & Mexia (2000)

Table S1 Parasitoid species reared from *Liriomyza trifolii*

Family	Sub-family	Parasitoid (current)	Parasitoid (in reference)	Location	Reference
Eulophidae	Eulophinae	*Diglyphus pulchripes* (Crawford, 1912)	*Diglyphus pulchripes*	Iran	Dousti *et al.* (2008)
Eulophidae	Eulophinae	*Diglyphus pulchripes* (Crawford, 1912)	*Diglyphus pulchripes*	USA	Stegmaier (1972)
Eulophidae	Eulophinae	*Diglyphus pusztensis* (Erdös and Novicky, 1951)	*Diglyphus pusztensis*	Japan	Nishino & Uchida (1999)
Eulophidae	Eulophinae	*Diglyphus* sp.	*Diglyphus* sp.	Egypt	Ali *et al.* (2020)
Eulophidae	Eulophinae	*Diglyphus* sp.	*Diglyphus* sp.	USA	Schuster & Price (1985)
Eulophidae	Eulophinae	*Elachertus* sp. *L*	*Elachertus* sp. *L*	Trinidad	Neuenschwander *et al.* (1987)
Eulophidae	Eulophinae	*Hemiptarsenus ornatus* (Nees, 1834)	*Hemiptarsenus dropion*	Israel	Freidberg & Gijswijtz (1983)
Eulophidae	Eulophinae	*Hemiptarsenus ornatus* (Nees, 1834)	*Hemiptarsenus dropion*	Italy	Del Bene (1989)
Eulophidae	Eulophinae	*Hemiptarsenus* sp.	*Hemiptarsenus* sp.	Turkey	Gençer (2004)
Eulophidae	Eulophinae	*Hemiptarsenus varicornis* (Girault, 1913)	*Hemiptarsenus varicornis*	China	Chen *et al.* (2006); Liu *et al.* (2012); Wang *et al.* (2017)
Eulophidae	Eulophinae	*Hemiptarsenus varicornis* (Girault, 1913)	*Hemitarsenus semiablaclavus*	Ethiopia	Abate (1987)
Eulophidae	Eulophinae	*Hemiptarsenus varicornis* (Girault, 1913)	*Hemiptarsenus semialbiclavus*	Guam	Schreiner *et al.* (1986)
Eulophidae	Eulophinae	*Hemiptarsenus varicornis* (Girault, 1913)	*Hemiptarsenus varicornis*	India	Srinivasan *et al.* (1995); Bhat *et al.* (2009); Sharma & Kumar (2017)
Eulophidae	Eulophinae	*Hemiptarsenus varicornis* (Girault, 1913)	*Hemiptarsenus varicornis*	Indonesia	Yasa *et al.* (2020)
Eulophidae	Eulophinae	*Hemiptarsenus varicornis* (Girault, 1913)	*Hemiptarsenus* sp. *near semialbiclavus*	Israel	Freidberg & Gijswijtz (1983)
Eulophidae	Eulophinae	*Hemiptarsenus varicornis* (Girault, 1913)	*Hemiptarsenus varicornis*	Japan	Saito *et al.* (1996); Amano *et al.* (2008); Arakaki & Kinjo (1998); Cai & Saito (2011)
Eulophidae	Eulophinae	*Hemiptarsenus varicornis* (Girault, 1913)	*Hemiptarsenus varicornis*	Philippines	Baucas *et al.* (2003); Arida *et al.* (2013)
Eulophidae	Eulophinae	*Hemiptarsenus varicornis* (Girault, 1913)	*Hemiptarsenus semialbiclava*	Senegal	Neuenschwander *et al.* (1987)
Eulophidae	Eulophinae	*Hemiptarsenus varicornis* (Girault, 1913)	*Hemiptarsenus varicornis*	Spain	Cabello *et al.* (1994)

Table S1 Parasitoid species reared from *Liriomyza trifolii*

Family	Sub-family	Parasitoid (current)	Parasitoid (in reference)	Location	Reference
Eulophidae	Eulophinae	*Hemiptarsenus varicornis* (Girault, 1913)	*Hemiptarsenus varicornis*	Taiwan	Lin & Wang (1992); Chien & Ku (1998)
Eulophidae	Eulophinae	*Hemiptarsenus zilahisebessi* Erdös, 1951	*Hemiptarsenus zilahisebessi*	Egypt	Ali *et al.* (2020)
Eulophidae	Eulophinae	*Hemiptarsenus zilahisebessi* Erdös, 1951	*Hemiptarsenus zilahisebessi*	Iran	Talebi *et al.* (2005); Asadi *et al.* (2006); Dousti *et al.* (2008)
Eulophidae	Eulophinae	*Hemiptarsenus zilahisebessi* Erdös, 1951	*Hemiptarsenus zilahisebessi*	South Korea	Cheol *et al.* (2002)
Eulophidae	Eulophinae	*Hemiptarsenus zilahisebessi* Erdös, 1951	*Hemiptarsenus zilahisebessi*	Spain	Cabello *et al.* (1994)
Eulophidae	Eulophinae	*Hemiptarsenus zilahisebessi* Erdös, 1951	*Hemiptarsenus zilahisebessi*	Turkey	Çikman (2006); Çikman & Çömlekçioğlu (2006); Çikman *et al.* (2006)
Eulophidae	Eulophinae	*Meruacesa liriomyzae* (Bouček 1988)	*Meruna liriomyzae*	Kenya	Bouček (1988)
Eulophidae	Eulophinae	*Pnigalio cristatus* (Ratzeburg, 1848)	*Ratzeburgiola cristatus*	Iran	Dousti *et al.* (2008)
Eulophidae	Eulophinae	*Pnigalio incompletus* (Bouček , 1971)	*Ratzeburgia incompleta*	Israel	Freidberg & Gijswijtz (1983)
Eulophidae	Eulophinae	*Pnigalio katonis* (Ishii, 1953)	*Pnigalio katonis*	Japan	Saito *et al.* (1996); Amano *et al.* (2008); Saito *et al.* (2008); Cai & Saito (2011)
Eulophidae	Eulophinae	*Pnigalio katonis* (Ishii, 1953)	*Pnigalio katonis*	Philippines	Baucas *et al.* (2003); Arida *et al.* (2013)
Eulophidae	Eulophinae	*Pnigalio soemius* (Walker, 1839)	*Pnigalio soemias*	Israel	Freidberg & Gijswijtz (1983)
Eulophidae	Eulophinae	*Pnigalio* sp.	*Pnigalio* sp.	Italy	Del Bene (1989)
Eulophidae	Eulophinae	*Pnigalio* sp.	*Pnigalio* sp.	USA	Hernández *et al.* (2009)
Eulophidae	Eulophinae	*Pnigalio* sp.- *nr. pectinicornis*	*Pnigalio* sp.- *nr. pectinicornis*	Iran	Talebi *et al.* (2005)
Eulophidae	Eulophinae	*Sympiesis acalle* (Walker, 1848)	*Sympiesis acalle*	Iran	Shahreki *et al.* (2016)
Eulophidae	Eulophinae	*Sympiesis gordius* (Walker, 1839)	*Sympiesis gordi*	Turkey	Çıkman & Uygun (2003)
Eulophidae	Eulophinae	*Sympiesis* sp.	*Sympiesis* sp.	Turkey	Ulubilir & Yabas (2000)
Eulophidae	Eulophinae	*Zagrammosoma americanum* Girault, 1916	*Zagrammosoma americanum*	USA	Chandler (1982)
Eulophidae	Eulophinae	*Zagrammosoma lineaticeps* (Girault, 1915)	*Mirzagrammosoma lineaticeps*	USA	Stegmaier (1966)
Eulophidae	Eulophinae	*Zagrammosoma* sp.	*Zagrammosoma* sp.	Egypt	Neama & Hegazi (2014); Ali *et al.* (2020)

Table S1 Parasitoid species reared from *Liriomyza trifolii*

Family	Sub-family	Parasitoid (current)	Parasitoid (in reference)	Location	Reference
Eulophidae	Eulophinae	*Zagrammosoma* sp.	*Zagrammosoma* sp.	USA	Hernández *et al.* (2009)
Eulophidae	Eulophinae	*Zagrammosoma talitzkii* Bouček, 1961	*Cirrospilus talitzkii*	Turkey	Çikman & La Salle (2011)
Eulophidae	Eulophinae	*Zagrammosoma variegatum* (Masi, 1907)	*Zagrammosoma variegatum*	India	Kapadia (1995)
Eulophidae	Tetrastichinae	*Aprostocetus* sp.	*Aprostocetus* sp.	Turkey	Çikman & Çömlekçioğlu (2006)
Eulophidae	Tetrastichinae	*Baryscapus impeditus* (Nees, 1834)	*Baryscapus impeditus*	Iran	Dousti *et al.* (2008)
Eulophidae	Tetrastichinae	*Neotrichoporoides szelenyii* (Erdös, 1951)	*Neotrichoporoides szelenyii*	Iran	Shahreki *et al.* (2016)
Eulophidae	Tetrastichinae	*Oomyzus* sp.	*Oomyzus* sp.	Japan	Saito *et al.* (1996)
Eulophidae	Tetrastichinae	*Quadrastichus liriomyzae* Hansson & LaSalle, 1996	*Quadrastichus liriomyzae*	Japan	Hansson & LaSalle (1996); Arakaki & Kinjo (1998)
Eulophidae	Tetrastichinae	*Quadrastichus liriomyzae* Hansson & LaSalle, 1996	*Quadrastichus liriomyzae*	Philippines	Baucas *et al.* (2003)
Eulophidae	Tetrastichinae	*Quadrastichus liriomyzae* Hansson & LaSalle, 1996	*Quadrastichus liriomyzae*	Taiwan	Hansson & LaSalle (1996); Chien & Ku (1998)
Eulophidae	Tetrastichinae	*Quadrastichus plaquoi* Reina & La Salle, 2004	*Quadrastichus plaquoi*	India	Reina & La Salle (2004); Sharma & Kumar (2017)
Eulophidae	Tetrastichinae	*Quadrastichus* sp.	*Quadrastichus* sp.	Japan	Cai & Saito (2011)
Figitidae	Eucoilinae	*Banacuniculus nigrimanus* (Kieffer, 1907)	*Ganaspidium nigrimanus*	Mexico	Valenzuela-Escoboza *et al.* (2010)
Figitidae	Eucoilinae	*Banacuniculus nigrimanus* (Kieffer, 1907)	*Ganaspidium nigrimanus*	USA	Hernández *et al.* (2009)
Figitidae	Eucoilinae	*Banacuniculus utilis* (Beardsley, 1988)	*Ganaspidium utilis*	Guam	Nafus & Schreiner (1989)
Figitidae	Eucoilinae	*Banacuniculus utilis* (Beardsley, 1988)	*Ganaspidium hunteri*	Tonga	Johnson (1993)
Figitidae	Eucoilinae	*Banacuniculus utilis* (Beardsley, 1988)	*Ganaspidium hunteri*	USA	Hara (1986); Lynch& Johnson (1987)
Figitidae	Eucoilinae	*Banacuniculus utilis* (Beardsley, 1988)	*Ganaspidius utilis*	Venezuela	Chirinos *et al.* (2014)
Figitidae	Eucoilinae	*Ganaspidium pusillae* Weld, 1955	*Ganaspidium pusillae*	USA	Hernández *et al.* (2009)
Figitidae	Eucoilinae	*Ganaspidium* sp.	*Ganaspidium* sp.	USA	Schuster & Price (1985)
Figitidae	Eucoilinae	*Gronotoma fetura* (Quinlan, 1986)	*Eucoilidea fetura*	Senegal	Neuenschwander *et al.* (1987)
Figitidae	Eucoilinae	*Gronotoma guamensis* (Yoshimoto, 1962)	*Eucoilidea guamensis*	Guam	Schreiner *et al.* (1986)
Figitidae	Eucoilinae	*Gronotoma micromorpha* (Perkins, 1910)	*Eucoilidea micromorpha*	Guam	Schreiner *et al.* (1986)

Table S1 Parasitoid species reared from *Liriomyza trifolii*

Family	Sub-family	Parasitoid (current)	Parasitoid (in reference)	Location	Reference
Figitidae	Eucoilinae	*Gronotoma micromorpha* (Perkins, 1910)	*Gronotoma micromorpha*	Japan	Arakaki & Kinjo (1998)
Figitidae	Eucoilinae	*Kleidotoma* sp.	*Kleidotoma* sp.	Japan	Saito *et al.* (1996)
Figitidae	Eucoilinae	*Nordlanderia plowa* Quinlan, 1986	*Nordlanderia plowa*	Senegal	Neuenschwander *et al.* (1987)
Figitidae	Eucoilinae	*Sinatra pacifica* (Yoshimoto, 1962)	*Cothonaspis pacifica*	Guam	Schreiner *et al.* (1986)
Figitidae	Eucoilinae	*Sinatra pacifica* (Yoshimoto, 1962)	*Disorygma pacifica*	Mexico	Valenzuela-Escoboza *et al.* (2010)
Figitidae	Eucoilinae	*Sinatra pacifica* (Yoshimoto, 1962)	*Disorygma pacifica*	USA	Hernández *et al.* (2009)
Figitidae	Eucoilinae	*Zaeucoila robusta* (Ashmead, 1894)	*Agrostocynips robusta*	USA	Hernández *et al.* (2009)
Herbertiidae		*Herbertia indica* Burks, 1959	*Herbertia indica*	India	Bhat *et al.* (2009)
Pteromalidae	Miscogastrinae	*Halticoptera aenea* (Walker, 1833)	*Halticoptera aenea*	USA	Palumbo *et al.* (1994)
Pteromalidae	Miscogastrinae	*Halticoptera circulus* (Walker, 1833)	*Halticoptera circulus*	Egypt	Neama & Hegazi (2014); Bayoumy *et al.* (2018); Ali *et al.* (2020)
Pteromalidae	Miscogastrinae	*Halticoptera circulus* (Walker, 1833)	*Halticoptera circulus*	Japan	Saito *et al.* (1996)
Pteromalidae	Miscogastrinae	*Halticoptera circulus* (Walker, 1833)	*Halticoptera circulus*	Senegal	Neuenschwander *et al.* (1987)
Pteromalidae	Miscogastrinae	*Halticoptera circulus* (Walker, 1833)	*Halticoptera circulus*	Trinidad	Neuenschwander *et al.* (1987)
Pteromalidae	Miscogastrinae	*Halticoptera circulus* (Walker, 1833)	*Halticoptera circulus*	USA	Stegmaier (1966); Trumble & Nakakihara (1983); Schuster & Price (1985); Neuenschwander *et al.* (1987); Schuster *et al.* (1991); Schuster & Wharton (1993)
Pteromalidae	Miscogastrinae	*Halticoptera longipetiolus* Hedqvist, 1975	*Halticoptera longipetiolus*	Turkey	Doganlar (2006)
Pteromalidae	Miscogastrinae	*Halticoptera nr. circulus*	*Halticoptera nr. circulus*	USA	Hernández *et al.* (2009)
Pteromalidae	Miscogastrinae	*Halticoptera patellana* (Dalman, 1818)	*Halticoptera patellana*	Turkey	Çikman (2012)
Pteromalidae	Miscogastrinae	*Halticoptera* sp.	*Halticoptera* sp.	Egypt	Neama & Hegazi (2014); Ali *et al.* (2020)
Pteromalidae	Miscogastrinae	*Halticoptera* sp. *1*	*Halticoptera* sp. *1*	Taiwan	Lin & Wang (1992)
Pteromalidae	Miscogastrinae	*Halticoptera* sp. *2*	*Halticoptera* sp. *2*	Taiwan	Lin & Wang (1992)
Pteromalidae	Pteromalinae	*Cyrtogaster vulgaris* Walker, 1833	*Cyrtogaster vulgaris*	Turkey	Çikman & Çömlekçioğlu (2006); Çikman *et al.* (2006)
Pteromalidae	Pteromalinae	*Sphegigaster brevicornis* (Walker, 1833)	*Sphegigaster brevicornis*	Turkey	Çikman *et al.* (2006)

Table S1 Parasitoid species reared from *Liriomyza trifolii*

**Family**	**Sub-family**	**Parasitoid (current)**	**Parasitoid (in reference)**	**Location**	**Reference**
Pteromalidae	Pteromalinae	*Sphegigaster hamugurivora* Ishii, 1953	*Sphegigaster hamugurivora*	Japan	Saito *et al.* (1996); Arakaki & Kinjo (1998)
Pteromalidae	Pteromalinae	*Sphegigaster* sp.	*Sphegigaster* sp.	Japan	Arakaki & Kinjo (1998)
Pteromalidae	Pteromalinae	*Trichomalopsis oryzae* Kamijo & Grissell, 1982	*Trichomalopsis oryzae*	Japan	Cai & Saito (2011)
Tetracampidae	Tetracampinae	*Epiclerus nomocerus* (Masi, 1934)	*Epiclerus nomocerus*	France	Franco & Panis (1991)

Table S1 Parasitoid species reared from *Liriomyza trifolii*

References

Abate T. 1987. New records of arthropod pests of grain legumes in Ethiopia. *Bean Improvement Cooperative Annual Report* **30**, 62–63.

Abul Fadl HAA & El-Khawas MAM. 2009. Incidence of parasitoids on the leaf-miner species, *Liriomyza trifolii* (Burgess) (Diptera: Agromyzidae), in tomato fields, at Qaluobia Governorate, Egypt. *Egyptian Journal of Biological Pest Control*, **19**, 93–97.

Al-Ghabeish I & Allawi TF. 2001. Agromyzid leaf miners and their parasitoids in Jordan. *Dirasat, Agricultural Sciences*, **28**, 172–177.

Ali SAM, Youssif MAI & Helaly SMMY. 2020. The leaf miner, *Liriomyza trifolii* (Burgess) and its parasitoids on faba bean plants. *Plant Archives* **20**, 1669-1680.

Amano K, Suzuki A, Hiromori H & Saito T (2008). Relative abundance of parasitoids reared during field exposure of sentinel larvae of the leafminers *Liriomyza trifolii* (Burgess), *L. sativae* Blanchard, and *Chromatomyia horticola* (Goureau) (Diptera: Agromyzidae). *Applied Entomology and Zoology* **43**, 625–630.

Arakaki N & Kinjo K. 1998. Notes on the parasitoid fauna of the serpentine leaf miner *Liriomyza trifolii* (Burgess) (Diptera: Agromyzidae) in Okinawa, southern Japan. *Applied Entomology and Zoology* **33**, 577–581.

Arida GS, Punzal BS, Shepard BM & Rajotte EG. 2013. Parasitism of leafminer *Liriomyza trifolii* (Burgess) (Diptera: Agromyzidae) larvae on onion grown after rice in Central Luzon Philippines. *The Philippine Entomologist* **27**, 100–108.

Asadi R, Talebi AA, Fathipour Y, Moharramipour S & Rakhshani E. 2006. Identification of parasitoids and seasonal parasitism of the agromyzid leafminers genus *Liriomyza* (Dip.: Agromyzidae) in Varamin, Iran. *Journal of Agricultural Science and Technology*. **8**, 293–303

Bassiony RA, Abou-Attia FA, Samy MA, Youssef AE & Ueno T. 2017. Parasitoid wasps attacking the American serpentine leafminer *Liriomyza trifolii* in Kafr EL-Shiekh, Egypt. *International Journal of Zoological Investigations* **3**, 15–20.

Baucas NS, Joshi RC, Verzola EA & Sacla GL. 2003. Exploratory survey of leafminer flies (Diptera: Agromyzidae) and their parasitoids in the highlands of Cordillera, Philippines. *Journal of Agriculture & Life Sciences* **37**, 43–58.

Bayoumy MH, Awadalla HS, Michaud JP & Ramadan MM. 2018. A life table for *Liriomyza trifolii* (Diptera: Agromyzidae) in a temperate zone of northeast Egypt with key factor analysis. *Environmental Entomology* **47**, 1047–1056.

Bhat DM, Bhagat R C & Qureshi AA. 2009. Records of some hymenopterous parasitoids of serpentine leaf miner, *Liriomyza trifolii* in vegetable ecosystems in Kashmir. *Indian Journal of Plant Protection* **37**, 188–189.

Bouček Z 1988. Australasian Chalcidoidea (Hymenoptera). *A biosystematic revision of genera of fourteen families, with a reclassification of species*. CAB International, Wallingford, UK, 832 pp.

Cabello T, Jaimez R & Pascual F. 1994. Distribución espacial y temporal de *Liriomyza* spp. y sus parasitoides en cultivos hortícolas en invernaderos del sur de España (Dip., Agromyzidae). *Boletín de Sanidad Vegetal* **20**, 445–455.

Cai D & Saito T. 2011. Current status of *Liriomyza* leafminers and their associated parasitoids in Shizuoka Prefecture. *Annual Report of the Kansai Plant Protection Society* **53**, 47–49.

Chandler LD 1982. Parasitization of cantaloupe infesting agromyzid leafminers in the Lower Rio Grande Valley, Texas. *The Southwestern Entomologist* 7, 94–97.

Chen W-L, P H, Liu Q-Y Ou , Gu D & Li Z-Z. 2006. [Research progress on parasitical natural enemies of leafminer in China]. *Guizhou Agricultural Sciences* **34**, 132-135.

Cheol MH, Sik CJ &Yeon H. 2002. Parasitism of *Liriomyza trifolii* (Dip.: Agromyzidae) by *Hemiptarsenus zilahisebessi* (Hymenoptera: Eulophidae) on tomato. *Korean Journal of Applied Entomology* **41**, 61–65.

Chien CC, & Ku HC 1998. The occurrence of *Liriomyza trifolii* (Diptera: Agromyzidae) and its parasitoids on fields of *Gerbera jamesonii*. *Chinese Journal of Entomology* **18**, 187–197 (in Chinese with English summary).

Chirinos DT, Díaz A & Geraud-Pouey F. 2014. Control biológico natural ejercido por parasitoides sobre el minador de la hoja *Liriomyza trifolii* (Burgess) (Diptera: Agromyzidae) en cebollín (*Allium fistulosum* L.). *Entomotropica* **29**, 129–138.

Civelek HS & La Salle J. 2005. Checklist of leafminer (Diptera: Agromyzidae) parasitoids in Turkey, with two new records. *Mitteilungen des Internationalen Entomologischen Vereinst* **30**, 21–28.

Çıkman & La Salle 2011. Parasitoids of the leafminers (Diptera: Agromyzidae) in Malatya, Turkey. *Türkiye Entomoloji Dergisi* **35**, 475–484.

Çıkman E & Çömlekçioğlu N. 2006. Effects of *Bacillus thuringiens*is on larval serpentine leafminers *Liriomyza trifolii* (Burgess) (Diptera: Agromyzidae) in bean. *Pakistan Journal of Biological Sciences* **9**, 2082-2086.

Çıkman E & Uygun N. 2003. The determination of leafminers (Diptera: Agromyzidae) and their parasitoids in cultivated and non-cultivated areas in Şanlıurfa province, southern Turkey. *Tu̇rkiye Entomoloji Dergisi*, **27**, 305–318.

Çıkman E, Beyarslan A & Civelek HS. 2006. Parasitoids of leafminers (Diptera: Agromyzidae) from southeast Turkey with 3 new records. *Turkish Journal of Zoology* **30**, 167–173.

Çıkman E. 2006. Parasitoids of the leafminers (Diptera: Agromyzidae) from Adıyaman province. *Tu̇rkiye Entomoloji Dergisi* **30**, 99–111.

Çıkman E. 2012. Parasitoids of the leafminers (Diptera: Agromyzidae) from Elazığ Province, Turkey. *African Journal of Agricultural Research* **7**, 1937-1943.

Del Bene G. 1989. Nemici naturali di *Liriomyza trifolii* (Burgess), *Chromatomyia horticola* (Goureau) e *Chromatomyia syngenesiae* Hardy (Diptera Agromyzidae) in Toscana. *Redia* **72**, 529–544.

Doganlar M. 2006. Systematic studies on some species of *Halticoptera* Spinola, 1811 (Hymenoptera: Chalcidoidea, Pteromalidae), with descriptions of new species. *Journal of Applied Sciences Research* **2**, 168–183.

Dousti AF, Kamali K, Ganbalani GN & Ostovan H. 2008. Report of four hymenopteran species of Eulophidae, parasitoids of *Liriomyza trifolii* (Dip.: Agromyzidae) in Shiraz, Iran. *Journal of Entomological Society of Iran* **27**, 9–10.

Franco E & Panis A. 1991. *Epiclerus nomocerus* (Masi) (Hym., Tetracampidae), nouveau parasitoide de *Liriomyza trifolii* Burgess (Dip., Agromyzidae) en culture sous serre. *International Organisation for Biological and Integrated Control /West Palaearctic Regional Section Bulletin* **14 (5)**, 129–133.

Freidberg A & Gijswijt MJ. 1983. A list and preliminary observations on natural enemies of the leaf miner, *Liriomyza trifolii* (Burgess) (Diptera: Agromyzidae) in Israel. *Israel Journal of Entomology* **17**, 115–116.

Gençer L. 2004. A study on the chalcidoid (Hymenoptera: Chalcidoidea) parasitoids of leafminers (Diptera: Agromyzidae) in Ankara Province. *Turkish Journal of Zoology* **28**, 119–122.

Gençer L. 2009. Contribution to the knowledge of the chalcid parasitoid complex (Hymenoptera: Chalcidoidea) of agromyzid leafminers (Diptera: Agromyzidae) from Turkey, with new hosts and records. *Journal of Plant Protection Research* **49**, 158-161.

Grenouillet C, Martinez M & Rasplus JY. 1993. Liste des parasitoïdes et des prédateurs des *Liriomyza* d'importance economique dans le monde (Diptera: Agromyzidae). *Colloque sur les mouches mineuses des plantes cultivees*, 24-26 Mar 1993, Montpellier, France, 143-156

Godinho M & Mexia A. 2000. Leafminers (*Liriomyza* sp.) importance in greenhouses in the Oeste region of Portugal and its natural parasitoids as control agents in IPM programs. *International Organisation for Biological and Integrated Control /West Palaearctic Regional Section Bulletin* **23 (1)**, 157—161.

Gupta A & Poorani J. 2009. Taxonomic studies on a collection of Chalcidoidea (Hymenoptera) from India with new distribution records. *Journal of Threatened Taxa* **1**, 300–304.

Hansson C & LaSalle J. 1996. Two new eulophid parasitoids (Hymenoptera: Chalcidoidea: Eulophidae) of *Liriomyza trifolii* (Burgess) (Diptera: Agromyzidae). *Oriental Insects* **30**, 193–202.

Hansson C & Navone P. 2017. Review of the European species of *Diglyphus* Walker (Hymenoptera: Eulophidae) including the description of a new species. *Zootaxa* **4269** (2), 197–229.

Hara AH. 1986. Effects of certain insecticides on *Liriomyza trifolii* (Burgess) (Diptera: Agromyzidae) and its parasitoids on chrysanthemums in Hawaii. *Proceedings of the Hawaiian Entomological Society* **6**, 65–70.

Hegazi E, Karam H, Neama A & Khafagi W. 2014. *Pseudopezomachus masii* Nixon (Hymenoptera: Braconidae: Alysiinae) a newly recorded parasitoid on *Liriomyza* spp. in Egypt. *Egyptian Journal of Biological Pest Control* **24**, 163–167.

Heinz KM & Parrella MP (1990). Holarctic distribution of the leafminer parasitoid *Diglyphus begini* (Ashmead) (Hymenoptera: Eulophidae) and notes on its life history attacking *Liriomyza trifolii* (Burgess) (Diptera: Agromyzidae) in chrysanthemum. *Annals of the Entomological Society of America* **83**, 916–924.

Hernández R, Harris M, Crosby K & Liu T-X. 2009 *Liriomyza* (Diptera: Agromyzidae) and parasitoid species on pepper in the Lower Rio Grande Valley of Texas. *The Southwestern Entomologist* **35**, 33–43

Holguín-Peña, RJ, Ramírez-Ahuja ML, Medina-Hernández D Torres, REP & Servín-Villegas R. 2019. Parasitoid wasps associated with *Liriomyza trifolii* mortality in pepper at Baja California Sur, Mexico. *The Southwestern Entomologist* **44**, 867–875.

Johnson MW. 1993. Biological control of *Liriomyza* leafminers in the Pacific basin. *Micronesica, Supplement* **4,** 81–92.

Kapadia MN. 1995, Varietal preference of castor leaf miner, *Liriomyza trifolii* (Burgess) and its parasitoids. *International Journal of Tropical Agriculture* **13**, 269–271.

Li J, Seal DR, Leibee GL & Liburd OE. 2012. Seasonal abundance and spatial distribution of the leafminer, *Liriomyza trifolii* (Diptera: Agromyzidae), and its parasitoid, *Opius dissitus* (Hymenoptera: Braconidae), on bean in southern Florida. *The Florida Entomologist* **95**, 128–135.

Lin F-C & Wang C-L. 1992. The occurrence of parasitoids of *Liriomyza trifolii* (Burgess) in Taiwan. *Chinese Journal of Entomology* **12**, 247–257.

Liu C-Y, Liu H, Zeng L & Lu Y-Y. 2012. [Research on spatial niche between two vegetable leafminers and their parasitoids]. *Journal of Environmental Entomology* **34**, 14–21 (in Chinese).

Lynch JA & Johnson MW. 1987. Stratified sampling of *Liriomyza* spp. (Diptera: Agromyzidae) and associated hymenopterous parasites on watermelon. *Journal of Economic Entomology* **80**, 1254-1261.

Murugan M & Dhandapani N. 2006. Bio-inoculant induced resistance in tomato, *Lycopersicon esculentum* Mill. attracts leafminer *Liriomyza trifolii* (Burgess) larval parasitoid *Chrysonotomyia rexia* Narendran (Hymenoptera: Eulophidae). *Journal of Plant Protection and Environment* **3**, 1–10.

Nafus D & Schreiner I. 1989. Biological control activities in the Mariana Islands from 1911 to 1988. *Micronesica* **22**, 65–106.

Neama AA & Hegazi EM. 2014. Parasitoids of the leaf miners *Liriomyza* spp. (Diptera: Agromyzidae) attacking faba bean in Alexandria, Egypt. *Egyptian Journal of Biological Pest Control* **24**, 301–305.

Neuenschwander P, Murphy ST & Coly EV. 1987. Introduction of exotic parasitic wasps for the control of *Liriomyza trifolii* (Dipt., Agromyzidae) in Senegal. *Tropical Pest Management* **33**, 290–297.

Nishino S & Uchida Y. 1999. [Occurrence of parasitic wasps of *Liriomyza trifolii* (Burgess) and influence on the harvested amount of crops of the eggplant]. *Bulletin of the Nara Agricultural Experiment Station* **30**, 11–16. (in Japanese).

Nishino S, Uchida Y, Fukui T & Kunimoto Y. 1997. [Occurrence of parasites of *Liriomyza trifolii* (Burgess) in Nara Prefecture]. *Proceedings of the Kansai Plant Protection Society* **39**, 9–10. (in Japanese).

Noyes JS. 1994. The reliability of published host-parasitoid records: a taxonomist's view. *Norwegian Journal of Agricultural Sciences Supplement* **16**, 59–69.

Olivera CR & Bordat D. 1996. Influence of *Liriomyza* species (Diptera: Agromyzidae) and their host plants, on oviposition by *Opius dissitus* females (Hymenoptera: Braconidae). *Annals of Applied Biology* **128**, 399–404.

Palumbo JC, Mullis H & Reyes FJ. 1994. Composition, seasonal abundance, and parasitism of *Liriomyza* (Diptera: Agromyzidae) species on lettuce in Arizona. *Journal of Economic Entomology* **87**, 1074–1076.

Parrella MP, Robb KL, Christie GD & Bethke JA. 1982. Control of *Liriomyza trifolii* with biological agents and insect growth regulators. *California Agriculture* **36**, 17–19.

Parshuram MS & Agnihotri M. 2017. A new species of *Chrysonotomyia* Ashmead (Hymenoptera: Eulophidae: Entedoninae) from Uttarakhand, India. *Journal of Entomology and Zoology Studies* **5**, 324–325.

Patel KJ & Schuster DJ. 1992. Hyperparasitism of *Liriomyza trifolii* (Burgess) on tomato. *The Florida Entomologist* **75**, 162.

Reina P & La Salle J. 2004. Two new species of *Quadrastichus* Girault (Hymenoptera: Eulophidae): parasitoids of the leafminers *Phyllocnistis citrella* Stainton (Lepidoptera: Gracillariidae) and *Liriomyza trifolii* (Burgess) (Diptera: Agromyzidae). *Journal of Hymenoptera Research* **13**, 108–119.

Reji GV, Prathapan KD & Rai H. 2003. Record of hymenopteran parasitoids of *Liriomyza trifolii* (Burgess) from Kerala. *Insect Environment* **9**, 30–31.

Rizzo MC & Massa B. 2002. Ecology of the eulophid parasitoid community living on hosts of spontaneous flora linked to citrus grove (Hymenoptera: Chalcidoidea: Eulophidae). *In:* (eds G Melika & C Thuróczy) *Parasitic wasps: evolution, systematics, biodiversity and biological control*. pp. 351–361. Agroinform Kiadó & Nyomda, Budapest, Hungary.

Saito T, Doi M, Tagami Y & Sugiyama K. 2008. Hymenopterous parasitoids of the exotic leafminers *Liriomyza trifolii* (Burgess) and *Liriomyza sativae* Blanchard (Diptera: Agromyzidae) in Shizuoka Prefecture, Japan. *Japanese Journal of Applied Entomology and Zoology* **52**, 225–229.

Saito T, Ikeda F & Ozawa A. 1996. Effect of pesticides on parasitoid complex of serpentine leafminer *Liriomyza trifolii* (Burgess) in Shizuoka Prefecture. *Japanese Journal of Applied Entomology and Zoology* **40**, 127–133.

Schreiner I, Nafus D & Bjork C. 1986. Control of *Liriomyza trifolii* (Burgess) (Dip.: Agromyzidae) on yard-long (*Vigna unguiculata*) and pole beans (*Phaseolus vulgaris*) on Guam: effect on yield loss and parasite numbers. *Tropical Pest Management* **32**, 333–337.

Schuster DJ & Price JF. 1985. Impact of insecticides on lepidopterous larval control and leafminer parasite emergence on tomato. *Proceedings of the Florida State Horticultural Society*. **98**, 248–251.

Schuster DJ & Wharton RA. 1993. Hymenopterous parasitoids of leaf-mining *Liriomyza* spp. (Diptera: Agromyzidae) on tomato in Florida. *Environmental Entomology* **22**, 1188–1191.

Schuster DJ, Gilreath JP, Wharton RA & Seymour PR. 1991. Agromyzidae (Diptera) leafminers and their parasitoids in weeds associated with tomato in Florida. *Environmental Entomology* **20**, 720–723.

Shahreki Z, Rakhshani E & Gumovsky A. 2016. Identification parasitoids of leafminer flies in Sistan region – Iran. *International Archive of Applied Sciences and Technology* **7**, 9–13.

Sharma PL & Kumar R. 2017. Diversity and abundance of parasitoids of *Liriomyza trifolii* in north-western Himalayas, India. *The Bioscan* **12**, 715–720.

Shaw MR. 1994. Parasitoid host ranges. In: *Parasitoid community ecology*. (eds BA Hawkins & Sheehan W). pp 451–471. Oxford University Press, Oxford, UK.

Srinivasan K, Viraktamath CA, Gupta M & Tewari GC. 1995. Geographical distribution, host range and parasitoids of serpentine leaf miner, *Liriomyza trifolii* (Burgess) in south India. *Pest Management in Horticultural Ecosystems* **1**, 93–100.

Stegmaier CE Jr. 1966. Host plants and parasites of *Liriomyza trifolii* in Florida (Diptera: Agromyzidae). *The Florida Entomologist* **49**, 75–80.

Stegmaier CE Jr. 1972. Parasitic Hymenoptera bred from the family Agromyzidae (Diptera) with special reference to south Florida. *The Florida Entomologist* **55**, 273–282.

Talebi AA, Asadi R, Fathipour Y, Kamali K, Moharramipour S & Rakhsbani E. 2005. Eulophid parasitoids of agromyzid leafminers genus *Liriomyza* (Dip.: Agromyzidae) in Tehran, Iran. *International Organisation for Biological and Integrated Control /West Palaearctic Regional Section Bulletin* **28 (1)**, 263–266.

Trumble JT & Nakakihara H. 1983. Occurrence, parasitism and sampling of *Liriomyza* species (Diptera: Agromyzidae) infesting celery in California. *Environmental Entomology* **12**, 810–814.

Ulubilir A & Yabas C. 2000.Studies on population development of leafminers (*Liriomyza* spp.) and parasitation situation. *Bulletin Section Regionale Ouest Palaearctique, Organisation Internationale de Lutte Biologique* **23 (1)**, 151–156

Valenzuela-Escoboza FA, Bautista-Martínez N, Lomelí-Flores JR, Cortez-Mondaca E & Valdez-Carrasco J. 2010. Natural parasitism of leafminer *Liriomyza trifolii* (Burgess) in jalapeño pepper in northern Sinaloa, Mexico. *The Southwestern Entomologist* **35**, 569–572.

Wang R, Cai D & Li J. 2017. Species of *Liriomyza* leafminers and their associated parasitoids in the cowpea planted in winter in Hainan Island. *Journal of Tropical Biology* **8**, 330–334.

Xing Z-L, Zhang L-Y, Wu S-Y, Yi H, Gao Y & Lei Z-G (2017). Niche comparison among two invasive leafminer species and their parasitoid *Opius biroi*: implications for competitive displacement. *Scientific Reports* 7, 4246 doi:10.1038/s41598–017–04562–3.

Yasa IW, Supartha IW & Susila IW. 2020. Kelimpahan populasi dan tingkat parasitisasi parasitoid indigenus terhadap hama invasif *Liriomyza trifolii* (Burgess) (Diptera: Agromyzidae) pada tanaman Asteraceae di Bali. *Agritrop* **10,** 59–66.

Zhu C-D, LaSalle J & Huang D-W. 2000. A review of the Chinese *Diglyphus* Walker (Hymenoptera: Eulophidae). *Oriental Insects* **34**, 263–288.

Table S2 Parasitoid species reared from *Liriomyza huidobrensis*

Specimens reared from mixed collections of *Liriomyza* spp., which included *L. huidobrensis*, were not included in the list. This table is based on data from Weintraub *et al.* (2017) with supplementary data added. As with any compilation of species names from the scientific literature, there will be uncertainty about the validity of some species and sampling intensity varies dramatically between countries, which usually reflects the scientific input into the problem rather than the actual size of the problem (Grenouillet *et al.* 1993; Noyes 1994; Shaw 1994). Given the nomenclatural changes that have occurred in time, the currently accepted name is given, together with the name used in the original reference. All references for the All references for the records are included.

**Family**	**Sub-family**	**Full Name**	**Parasitoid name in text**	**Location**	**Reference**
Braconidae	Alysiinae	*Dacnusa sasakawai* Takada, 1977	*Dacnusa sasakawai*	Japan	Shindo *et al.* (2005)
Braconidae	Alysiinae	*Dacnusa sibirica* Telenga, 1935	*Dacnusa sibirica*	Austria	Stolz & Blümel (1998)
Braconidae	Alysiinae	*Dacnusa sibirica* Telenga, 1935	*Dacnusa sibirica*	China	Pan *et al.* (2019)
Braconidae	Alysiinae	*Dacnusa sibirica* Telenga, 1935	*Dacnusa sibirica*	Italy	Burgio *et al.* (2005)
Braconidae	Alysiinae	*Dacnusa sibirica* Telenga, 1935	*Dacnusa sibirica*	Portugal	Godinho & Mexia (2000); Mexia *et al.* (2004)
Braconidae	Alysiinae	*Dacnusa sibirica* Telenga, 1935	*Dacnusa sibirica*	Réunion	Vayssieres *et al.* (2001)
Braconidae	Alysiinae	*Dacnusa sibirica* Telenga, 1935	*Dacnusa sibirica*	The Netherlands	van der Linden (1990, 1991)
Braconidae	Alysiinae	*Dacnusa* sp.	*Dacnusa* sp.	Canada	Bahlai *et al.* (2006)
Braconidae	Alysiinae	*Dacnusa* sp.	*Dacnusa* sp.	China	Pan *et al.* (2019)
Braconidae	Alysiinae	*Oenonogastra* sp.	*Oenonogastra* sp.	Costa Rica	Carballo *et al.* (1990); Rodriguez (1997); Weintraub *et al.* (2017)
Braconidae	Braconinae	*Bracon intercessor* Nees, 1834	*Bracon intercessor*	Turkey	Civelek *et al.* (2002)
Braconidae	Opiinae	*Opius caricivorae* Fischer, 1964	*Opius caricivorae*	Taiwan	Chien & Chang (2012)
Braconidae	Opiinae	*Opius chromatomyiae* Belokobylskij & Wharton, 2004	*Opius chromatomyiae*	Indonesia	Hidrayani *et al.* (2005)
Braconidae	Opiinae	*Opius dimidiatus* (Ashmead, 1889)	*Opius dimidiatus*	China	Chen *et al.* (2000)
Braconidae	Opiinae	*Opius dimidiatus* (Ashmead, 1889)	*Opius dimidiatus*	Guatemala	Weintraub *et al.* (2017)
Braconidae	Opiinae	*Opius dissitus* Muesebeck, 1963	*Opius dissitus*	China	Song *et al.* (2003)
Braconidae	Opiinae	*Opius dissitus* Muesebeck, 1963	*Opius dissitus*	Réunion	Olivera & Bordat (1996)
Braconidae	Opiinae	*Opius dissitus* Muesebeck, 1963	*Opius dissitus*	Kenya	Foba *et al.* (2015)
Braconidae	Opiinae	*Opius dissitus* Muesebeck, 1963	*Opius dissitus*	Guatemala	Weintraub *et al.* (2017)
Braconidae	Opiinae	*Opius mandibularis* Gahan, 1915	*Opius mandibularis*	Guatemala	Weintraub *et al.* (2017)
Braconidae	Opiinae	*Opius meracus* Fischer, 1960	*Opius meracus*	Turkey	Civelek *et al.* (2002)
Braconidae	Opiinae	*Opius pallipes* Wesmael, 1835	*Opius pallipes*	Malaysia	Nor Ahya & Nur Liyana (2018)
Braconidae	Opiinae	*Opius pallipes* Wesmael, 1835	*Opius pallipes*	The Netherlands	van der Linden (1990)

Table S2 Parasitoid species reared from *Liriomyza huidobrensis*

Family	Sub-family	Full Name	Parasitoid name in text	Location	Reference
Braconidae	Opiinae	*Opius* sp.	*Opius* sp.	Brazil	Pereira *et al.* (2002); Guimarães *et al.* (2009); Dequech *et al.* (2010)
Braconidae	Opiinae	*Opius* sp.	*Opius* sp.	Canada	Bahlai *et al.* (2006)
Braconidae	Opiinae	*Opius* sp.	*Opius* sp.	China	Chen *et al.* (2000); Luo *et al.* (2001); Chen *et al.* (2011)
Braconidae	Opiinae	*Opius* sp.	*Opius* sp.	Columbia	Hincapie *et al.* (1993)
Braconidae	Opiinae	*Opius* sp.	*Opius* sp.	Costa Rica	Carballo *et al.* (1990); Rodriguez (1997); Weintraub *et al.* (2017)
Braconidae	Opiinae	*Opius* sp.	*Opius* sp.	Indonesia	Prijono *et al.* (2004)
Braconidae	Opiinae	*Opius* sp.	*Opius* sp.	Israel	Weintraub *et al.* (2017)
Braconidae	Opiinae	*Opius* sp.	*Opius* sp.	Japan	Shindo *et al.* (2005)
Braconidae	Opiinae	*Opius* sp.	*Opius* sp.	Jordan	Al-Ghabeish & Allawi (2001)
Braconidae	Opiinae	*Opius* sp.	*Opius* sp.	Malaysia	Sivapragasm *et al.* (1999)
Braconidae	Opiinae	*Opius* sp.	*Opius* sp.	Peru	Delgado & Aguilar (1980); Redolfi *et al.* (1985)
Braconidae	Opiinae	*Opius* sp.	*Opius* sp.	Philippines	Baucas *et al.* (2003)
Braconidae	Opiinae	*Opius* sp.	*Opius* sp.	Sri Lanka	Nugaliyada (2000)
Braconidae	Opiinae	*Opius (Phaedrotoma) luteoclypealis* van Achterberg & Salvo, 1997	*Phaedrotoma luteoclypealis*	Argentina	van Achterberg & Salvo (1997); Salvo *et al.* (2005)
Braconidae	Opiinae	*Opius (Phaedrotoma) mesoclypealis* van Achterberg & Salvo, 1997	*Phaedrotoma mesoclypealis*	Argentina	van Achterberg & Salvo (1997); Salvo *et al.* (2005)
Braconidae	Opiinae	*Opius (Phaedrotoma) scabriventris* Nixon, 1955	*Phaedrotoma scabriventris*	Argentina	van Achterberg & Salvo (1997); Salvo & Valladares (2002); Salvo *et al.* (2005)
Braconidae	Opiinae	*Opius (Phaedrotoma) scabriventris* Nixon, 1955	*Phaedrotoma scabriventris*	Peru	Delgado & Aguilar (1980)
Braconidae	Opiinae	*Opius (Phaedrotoma)* sp.	*Phaedrotoma* sp.	Argentina	van Achterberg & Salvo (1997)
Diapriidae	Diapriinae	*Trichopria* sp.	*Trichopria* sp.	Portugal	Mexia *et al.* (2004)
Eulophidae	Entedoninae	*Asecodes delucchii* (Bouček, 1971)	*Asecodes delucchii*	Indonesia	Hidrayani *et al.* (2005)
Eulophidae	Entedoninae	*Asecodes delucchii* (Bouček, 1971)	*Asecodes delucchii*	Philippines	Baucas *et al.* (2003)
Eulophidae	Entedoninae	*Asecodes* sp.	*Asecodes* sp.	Indonesia	Rauf & Shepard (2001)
Eulophidae	Entedoninae	*Chrysocharis ainsliei* Crawford, 1912	*Chrysocharis ainsliei*	Peru	Delgado & Aguilar (1980)

Table S2 Parasitoid species reared from *Liriomyza huidobrensis*

Family	Sub-family	Full Name	Parasitoid name in text	Location	Reference
Eulophidae	Entedoninae	*Chrysocharis bedius* (Walker, 1842)	*Chrysocharis bedius*	Brazil	Dequech *et al.* (2010)
Eulophidae	Entedoninae	*Chrysocharis bedius* (Walker, 1842)	*Chrysocharis* sp. cerca *bedius*	Peru	Sanchez & Redolfi (1985)
Eulophidae	Entedoninae	*Chrysocharis bedius* (Walker, 1842)	*Chrysocharis bedius*	Reunion	Vayssieres *et al.* (2001)
Eulophidae	Entedoninae	*Chrysocharis c.f. aluta*	*Chrysocharis c.f. aluta*	Guatemala	Weintraub *et al.* (2017)
Eulophidae	Entedoninae	*Chrysocharis caribea* Bouček, 1977	*Chrysocharis caribea*	Argentina	Salvo & Valladares (1997); Salvo *et al.* (2005)
Eulophidae	Entedoninae	*Chrysocharis caribea* Bouček, 1977	*Chrysocharis caribea*	Peru	Weintraub *et al.* (2017)
Eulophidae	Entedoninae	*Chrysocharis flacilla* (Walker, 1842)	*Chrysocharis flacilla*	Argentina	Arce de Hamity & Neder de Román (1984); Salvo & Valladares (1997); Salvo *et al.* (2005)
Eulophidae	Entedoninae	*Chrysocharis flacilla* (Walker, 1842)	*Chrysocharis flacilla*	Chile	Neder de Román & Arce de Hamity (1984)
Eulophidae	Entedoninae	*Chrysocharis flacilla* (Walker, 1842)	*Chrysocharis phytomyzae*, *Chrysocharis flacilla*	Peru	Redolfi *et al.* (1985), Iannacone (1998); Delgado & Aguilar (1980); Weintraub *et al.* (2017)
Eulophidae	Entedoninae	*Chrysocharis ignota* Hansson, (1987)	*Chrysocharis ignota*	Guatemala	MacVean & Perez (1997)
Eulophidae	Entedoninae	*Chrysocharis orbicularis* (Nees, 1834)	*Chrysocharis orbicularis*	Jordan	Al-Ghabeish & Allawi (2001)
Eulophidae	Entedoninae	*Chrysocharis oscinidis* Ashmead, 1888	*Chrysocharis oscinidis*	Canada	Bahlai *et al.* (2006)
Eulophidae	Entedoninae	*Chrysocharis pentheus* (Walker, 1839)	*Chrysocharis pentheus*	China	Chen *et al.* (2000); Pan *et al.* (2019)
Eulophidae	Entedoninae	*Chrysocharis pentheus* (Walker, 1839)	*Chrysocharis pentheus*	Israel	Weintraub *et al.* (2017)
Eulophidae	Entedoninae	*Chrysocharis pentheus* (Walker, 1839)	*Chrysocharis pentheus*	Japan	Shindo *et al.* (2005)
Eulophidae	Entedoninae	*Chrysocharis pentheus* (Walker, 1839)	*Chrysocharis pentheus*	Malaysia	Sivapragasm *et al.* (1999); Nor Ahya & Nur Liyana (2018)
Eulophidae	Entedoninae	*Chrysocharis pentheus* (Walker, 1839)	*Chrysocharis pentheus*	Taiwan	Chien & Chang (2013)
Eulophidae	Entedoninae	*Chrysocharis pubicornis* (Zetterstedt, 1838)	*Chrysocharis pubicornis*	China	Song *et al.* (2003); Pan et a. (2019)
Eulophidae	Entedoninae	*Chrysocharis pubicornis* (Zetterstedt, 1838)	*Chrysocharis pubicornis*	Japan	Shindo *et al.* (2005)
Eulophidae	Entedoninae	*Chrysocharis pubicornis* (Zetterstedt, 1838)	*Chrysocharis pubicornis*	Jordan	Al-Ghabeish & Allawi (2001)
Eulophidae	Entedoninae	*Chrysocharis* sp.	*Chrysocharis* sp.	Argentina	Salvo & Valladares (1995)
Eulophidae	Entedoninae	*Chrysocharis* sp.	*Chrysocharis* sp.	China	Pan *et al.* (2019)

Table S2 Parasitoid species reared from *Liriomyza huidobrensis*

Family	Sub-family	Full Name	Parasitoid name in text	Location	Reference
Eulophidae	Entedoninae	*Chrysocharis* sp.	*Chrysocharis* sp.	Costa Rica	Carballo *et al.* (1990); Hidalgo & Carballo (1991); Rodriguez (1997); Weintraub *et al.* (2017)
Eulophidae	Entedoninae	*Chrysocharis* sp.	*Chrysocharis* sp.	Indonesia	Rauf & Shepard (2001)
Eulophidae	Entedoninae	*Chrysocharis* sp.	*Chrysocharis* sp.	Peru	Delgado & Aguilar (1980); Redolfi *et al.* (1985); Weintraub *et al.* (2017)
Eulophidae	Entedoninae	*Chrysocharis* sp.	*Chrysocharis* sp.	Portugal	Mexia *et al.* (2004)
Eulophidae	Entedoninae	*Chrysocharis tristis* Hansson, 1987	*Chrysocharis tristis*	Guatemala	Weintraub *et al.* (2017)
Eulophidae	Entedoninae	*Chrysocharis vonones* (Walker, 1839)	*Chrysocharis vonones*	Argentina	Salvo *et al.* (2005); Salvo & Valladares (1997)
Eulophidae	Entedoninae	*Chrysocharis vonones* (Walker, 1839)	*Chrysocharis brethesi*	Peru	Weintraub *et al.* (2017)
Eulophidae	Entedoninae	*Chrysonotomyia* sp.	*Chrysonotomyia* sp.	Argentina	Salvo & Valladares (1995); Salvo *et al.* (2005)
Eulophidae	Entedoninae	*Chrysonotomyia* sp.	*Chrysonotomyia* sp.	Peru	Weintraub *et al.* (2017)
Eulophidae	Entedoninae	*Closterocerus cinctipennis* Ashmead, 1888	*Closterocerus cinctipennis*	Peru	Weintraub *et al.* (2017)
Eulophidae	Entedoninae	*Closterocerus pulcher* (Howard, 1897)	*Closterocerus pulcher*	Guatemala	Weintraub *et al.* (2017)
Eulophidae	Entedoninae	*Closterocerus* sp.	*Closterocerus* sp.	Brazil	Guimarães *et al.* (2010)
Eulophidae	Entedoninae	*Closterocerus* sp.	*Closterocerus* sp.	Indonesia	Rauf & Shepard (2001)
Eulophidae	Entedoninae	*Closterocerus* sp.	*Closterocerus* sp.	Peru	Delgado & Aguilar (1980)
Eulophidae	Entedoninae	*Neochrysocharis beasleyi* Fisher & La Salle, 2005	*Neochrysocharis beasleyi*	Indonesia	Fisher & La Salle (2005)
Eulophidae	Entedoninae	*Neochrysocharis beasleyi* Fisher & La Salle, 2005	*Neochrysocharis beasleyi*	Vietnam	Fisher & La Salle (2005)
Eulophidae	Entedoninae	*Neochrysocharis diastatae* (Howard, 1881)	*Neochrysocharis diastatae*	Guatemala	Weintraub *et al.* (2017)
Eulophidae	Entedoninae	*Neochrysocharis formosa* (Westwood, 1833)	*Neochrysocharis formosa*	China	Chen *et al.* (2000); Pan *et al.* (2019)
Eulophidae	Entedoninae	*Neochrysocharis formosa* (Westwood, 1833)	*Neochrysocharis formosa*	Indonesia	Hidrayani *et al.* (2005)
Eulophidae	Entedoninae	*Neochrysocharis formosa* (Westwood, 1833)	*Neochrysocharis formosa*	Israel	Weintraub *et al.* (2017)
Eulophidae	Entedoninae	*Neochrysocharis formosa* (Westwood, 1833)	*Neochrysocharis formosa*	Jordan	Al-Ghabeish & Allawi (2001)
Eulophidae	Entedoninae	*Neochrysocharis formosa* (Westwood, 1833)	*Neochrysocharis formosa*	Kenya	Foba *et al.* (2016)
Eulophidae	Entedoninae	*Neochrysocharis formosa* (Westwood, 1833)	*Neochrysocharis formosa*	Malaysia	Sivapragasm *et al.* (1999)
Eulophidae	Entedoninae	*Neochrysocharis formosa* (Westwood, 1833)	*Neochrysocharis formosa*	Turkey	Civelek *et al.* (2002)

Table S2 Parasitoid species reared from *Liriomyza huidobrensis*

Family	Sub-family	Full Name	Parasitoid name in text	Location	Reference
Eulophidae	Entedoninae	*Neochrysocharis okazakii* Kamijo, 1978	*Neochrysocharis okazakii*	Japan	Shindo *et al.* (2005)
Eulophidae	Entedoninae	*Neochrysocharis okazakii* Kamijo, 1978	*Neochrysocharis okazakii*	Philippines	Baucas *et al.* (2003)
Eulophidae	Entedoninae	*Neochrysocharis okazakii* Kamijo, 1978	*Closterocerus okazakii*	Taiwan	Chien & Chang (2013)
Eulophidae	Entedoninae	*Neochrysocharis* sp.	*Neochrysocharis* sp.	Indonesia	Rauf & Shepard (2001)
Eulophidae	Entedoninae	*Pediobius metallicus* (Nees, 1834)	*Pediobius metallicus*	China	Song *et al.* (2003); Pan *et al.* (2019)
Eulophidae	Entedoninae	*Pediobius metallicus* (Nees, 1834)	*Pediobius metallicus*	Israel	Weintraub *et al.* (2017)
Eulophidae	Entedoninae	*Pediobius metallicus* (Nees, 1834)	*Pediobius metallicus*	Jordan	Al-Ghabeish & Allawi (2001)
Eulophidae	Entedoninae	*Proacrias* sp.	*Proacrias* sp.	Easter Island	Ripa *et al.* (1995)
Eulophidae	Entedoninae	*Proacrias thysanoides* (De Santis, 1972)	*Chrysonotomyia thysanoides*	Argentina	Salvo & Valladares (1995); Salvo *et al.* (2005)
Eulophidae	Entedoninae	*Proacrias thysanoides* (De Santis, 1972)	*Chrysonotomyia thysanoides*	Peru	Weintraub *et al.* (2017)
Eulophidae	Entedoninae	*Proacrias xenodice* (Walker, 1842)	*Chrysonotomyia xenodice*	Argentina	Salvo & Valladares (1995); Salvo *et al.* (2005); Videla *et al.* (2006)
Eulophidae	Entedoninae	*Proacrias xenodice* (Walker, 1842)	*Chrysonotomyia xenodice*	Easter Island	Ripa *et al.* (1995)
Eulophidae	Eulophinae	*Burkseus vittatus* (Walker, 1838)	*Cirrospilus vittatus*	China	Pan *et al.* (2019)
Eulophidae	Eulophinae	*Burkseus vittatus* (Walker, 1838)	*Cirrospilus vittatus*	Jordan	Al-Ghabeish & Allawi (2001)
Eulophidae	Eulophinae	*Cirrospilus ambiguus* Hansson & LaSalle, 1996	*Cirrospilus ambiguus*	Indonesia	Rauf & Shepard (2001)
Eulophidae	Eulophinae	*Diaulinopsis callichroma* Crawford, 1912	*Diaulinopsis callichroma*	Peru	Weintraub *et al.* (2017)
Eulophidae	Eulophinae	*Diaulinopsis* sp.	*Diaulinopsis* sp.	Argentina	Salvo & Valladares (1995)
Eulophidae	Eulophinae	*Diaulinopsis* sp.	*Diaulinopsis* sp.	Peru	Weintraub *et al.* (2017)
Eulophidae	Eulophinae	*Diglyphus albiscapus*	*Diglyphus albiscapus*	Japan	Shindo *et al.* (2005)
Eulophidae	Eulophinae	*Diglyphus begini* (Ashmead, 1904)	*Diglyphus begini*	Argentina	Salvo *et al.* (2005)
Eulophidae	Eulophinae	*Diglyphus begini* (Ashmead, 1904)	*Diglyphus begini*	China	Song *et al.* (2003)
Eulophidae	Eulophinae	*Diglyphus begini* (Ashmead, 1904)	*Diglyphus begini*	Columbia	Cure & Cantor (2003)
Eulophidae	Eulophinae	*Diglyphus begini* (Ashmead, 1904)	*Diglyphus begini*	Peru	Galantini & Redolfi (1992); Weintraub *et al.* (2017)
Eulophidae	Eulophinae	*Diglyphus bimaculatus* Zhu, LaSalle & Huang, 2000	*Diglyphus bimaculatus*	China	Pan *et al.* (2019)
Eulophidae	Eulophinae	*Diglyphus crassinervis* Erdös, 1958	*Diglyphus crassinervis*	China	Song *et al.* (2003)
Eulophidae	Eulophinae	*Diglyphus crassinervis* Erdös, 1958	*Diglyphus crassinervis*	Israel	Weintraub *et al.* (2017)
Eulophidae	Eulophinae	*Diglyphus crassinervis* Erdös, 1958	*Diglyphus crassinervis*	Jordan	Al-Ghabeish & Allawi (2001)

Table S2 Parasitoid species reared from *Liriomyza huidobrensis*

Family	Sub-family	Full Name	Parasitoid name in text	Location	Reference
Eulophidae	Eulophinae	*Diglyphus crassinervis* Erdös, 1958	*Diglyphus crassinervis*	Portugal	Godinho & Mexia (2000); Mexia *et al.* (2004)
Eulophidae	Eulophinae	*Diglyphus crassinervis* Erdös, 1958	*Diglyphus crassinervis*	Turkey	Civelek *et al.* (2002); Çıkman *et al.* (2006)
Eulophidae	Eulophinae	*Diglyphus intermedius* (Girault, 1916)	*Diglyphus intermedius*	China	Chen *et al.* (2000)
Eulophidae	Eulophinae	*Diglyphus intermedius* (Girault, 1916)	*Diglyphus intermedius*	Columbia	Andrade *et al.* (1989)
Eulophidae	Eulophinae	*Diglyphus intermedius* (Girault, 1916)	*Diglyphus intermedius*	Costa Rica	Carballo *et al.* (1990)
Eulophidae	Eulophinae	*Diglyphus intermedius* (Girault, 1916)	*Diglyphus intermedius*	Guatemala	Weintraub *et al.* (2017)
Eulophidae	Eulophinae	*Diglyphus isaea* (Walker, 1833)	*Diglyphus isaea*	Portugal	Mexia *et al.* (2004)
Eulophidae	Eulophinae	*Diglyphus isaea* (Walker, 1838)	*Diglyphus isaea*	Austria	Stolz & Blümel (1998)
Eulophidae	Eulophinae	*Diglyphus isaea* (Walker, 1838)	*Diglyphus isaea*	China	Chen *et al.* (2000); Luo *et al.* (2000); Luo *et al.* (2001); Chen *et al.* (2011); Pan *et al.* (2019)
Eulophidae	Eulophinae	*Diglyphus isaea* (Walker, 1838)	*Diglyphus isaea*	Costa Rica	Rodriguez (1997)
Eulophidae	Eulophinae	*Diglyphus isaea* (Walker, 1838)	*Diglyphus isaea*	Guatemala	Weintraub *et al.* (2017)
Eulophidae	Eulophinae	*Diglyphus isaea* (Walker, 1838)	*Diglyphus isaea*	Israel	Weintraub & Horowitz (1996); Weintraub (1999)
Eulophidae	Eulophinae	*Diglyphus isaea* (Walker, 1838)	*Diglyphus isaea*	Italy	Calabretta *et al.* (1995); Burgio *et al.* (2005)
Eulophidae	Eulophinae	*Diglyphus isaea* (Walker, 1838)	*Diglyphus isaea*	Japan	Shindo *et al.* (2005)
Eulophidae	Eulophinae	*Diglyphus isaea* (Walker, 1838)	*Diglyphus isaea*	Jordan	Al-Ghabeish & Allawi (2001)
Eulophidae	Eulophinae	*Diglyphus isaea* (Walker, 1838)	*Diglyphus isaea*	Kenya	Foba *et al.* (2016)
Eulophidae	Eulophinae	*Diglyphus isaea* (Walker, 1838)	*Diglyphus isaea*	Lebanon	Noujeim *et al.* (2013)
Eulophidae	Eulophinae	*Diglyphus isaea* (Walker, 1838)	*Diglyphus isaea*	Philippines	Baucas *et al.* (2003)
Eulophidae	Eulophinae	*Diglyphus isaea* (Walker, 1838)	*Diglyphus isaea*	Portugal	Godinho & Mexia (2000)
Eulophidae	Eulophinae	*Diglyphus isaea* (Walker, 1838)	*Diglyphus isaea*	Sri Lanka	Nugaliyada (2000)
Eulophidae	Eulophinae	*Diglyphus isaea* (Walker, 1838)	*Diglyphus isaea*	The Netherlands	van der Linden (1990)
Eulophidae	Eulophinae	*Diglyphus isaea* (Walker, 1838)	*Diglyphus isaea*	Turkey	Civelek *et al.* (2002)
Eulophidae	Eulophinae	*Diglyphus minoeus* (Walker, 1838)	*Diglyphus minoeus*	China	Pan *et al.* (2019)
Eulophidae	Eulophinae	*Diglyphus minoeus* (Walker, 1838)	*Diglyphus minoeus*	Portugal	Mexia *et al.* (2004)
Eulophidae	Eulophinae	*Diglyphus minoeus* (Walker, 1838)	*Diglyphus minoeus*	Turkey	Çıkman *et al.* (2006)
Eulophidae	Eulophinae	*Diglyphus pachyneurus* Graham, 1963	*Diglyphus pachyneurus*	China	Song *et al.* (2003)

Table S2 Parasitoid species reared from *Liriomyza huidobrensis*

**Family**	**Sub-family**	**Full Name**	**Parasitoid name in text**	**Location**	**Reference**
Eulophidae	Eulophinae	*Diglyphus pedicellus* Gordh & Hendrickson, 1979	*Diglyphus pedicellus*	Argentina	Salvo & Valladares (1995); Salvo *et al.* (2005)
Eulophidae	Eulophinae	*Diglyphus poppoea* Walker, 1848	*Diglyphus poppoea*	Portugal	Godinho & Mexia (2000); Mexia *et al.* (2004)
Eulophidae	Eulophinae	*Diglyphus pulchripes* (Crawford, 1912)	*Diglyphus pulchripes*	China	Song *et al.* (2003)
Eulophidae	Eulophinae	*Diglyphus* sp.	*Diglyphus* sp.	Argentina	Arce de Hamity & Neder de Roman (1984)
Eulophidae	Eulophinae	*Diglyphus* sp.	*Diglyphus* sp.	China	Chen *et al.* (2000)
Eulophidae	Eulophinae	*Diglyphus* sp.	*Diglyphus* sp.	China	Pan *et al.* (2019)
Eulophidae	Eulophinae	*Diglyphus* sp.	*Diglyphus* sp.	Columbia	Hincapie *et al.* (1993)
Eulophidae	Eulophinae	*Diglyphus* sp.	*Diglyphus* sp.	Costa Rica	Weintraub *et al.* (2017)
Eulophidae	Eulophinae	*Diglyphus* sp.	*Diglyphus* sp.	Peru	Iannacone & Reyes (2001)
Eulophidae	Eulophinae	*Diglyphus* sp.	*Diglyphus* sp.	South Africa	Visser & Weintraub (2001)
Eulophidae	Eulophinae	*Diglyphus* sp. *(near intermedius)*	*Diglyphus* sp. *(near intermedius)*	Costa Rica	Hidalgo & Carballo (1991)
Eulophidae	Eulophinae	*Diglyphus wani* Liu, Zhu & Yefremova, 2018	*Diglyphus wani*	China	Pan *et al.* (2019)
Eulophidae	Eulophinae	*Diglyphus websteri* (Crawford, 1912)	*Diglyphus websteri*	Argentina	Salvo & Valladares (1995); Salvo *et al.* (2005); Videla *et al.* (2006)
Eulophidae	Eulophinae	*Diglyphus websteri* (Crawford, 1912)	*Diglyphus websteri*	Guatemala	Weintraub *et al.* (2017)
Eulophidae	Eulophinae	*Diglyphus websteri* (Crawford, 1912)	*Solenotus websteri* , *Diglyphus websteri*	Peru	Campos (1977), Redolfi *et al.* (1985); Sanchez & Redolfi (1985); Iannacone (1998); Weintraub *et al.* (2017)
Eulophidae	Eulophinae	*Hemiptarsenus fulvicollis* Westwood, 1833	*Hemiptarsenus fulvicollis*	Portugal	Mexia *et al.* (2004)
Eulophidae	Eulophinae	*Hemiptarsenus ornatus* (Nees, 1834)	*Hemiptarsenus ornatus*	Jordan	Al-Ghabeish & Allawi (2001)
Eulophidae	Eulophinae	*Hemiptarsenus* sp.	*Hemiptarsenus* sp.	China	Pan *et al.* (2019)
Eulophidae	Eulophinae	*Hemiptarsenus* sp.	*Hemiptarsenus* sp.	Jordan	Al-Ghabeish & Allawi (2001)
Eulophidae	Eulophinae	*Hemiptarsenus unguicellus* (Zetterstedt, 1838)	*Hemiptarsenus unguicellus*	China	Song *et al.* (2003)
Eulophidae	Eulophinae	*Hemiptarsenus varicornis* (Girault, 1913)	*Hemiptarsenus varicornis*	China	Chen *et al.* (2000); Chen *et al.* (2011)

Table S2 Parasitoid species reared from *Liriomyza huidobrensis*

Family	Sub-family	Full Name	Parasitoid name in text	Location	Reference
Eulophidae	Eulophinae	*Hemiptarsenus varicornis* (Girault, 1913)	*Hemiptarsenus varicornis*	Indonesia	Shepard *et al.* (1998); Prijono *et al.* (2004)
Eulophidae	Eulophinae	*Hemiptarsenus varicornis* (Girault, 1913)	*Hemiptarsenus varicornis*	Kenya	Foba *et al.* (2016)
Eulophidae	Eulophinae	*Hemiptarsenus varicornis* (Girault, 1913)	*Hemiptarsenus varicornis*	Malaysia	Sivapragasm *et al.* (1999); Nor Ahya & Nur Liyana (2018)
Eulophidae	Eulophinae	*Hemiptarsenus varicornis* (Girault, 1913)	*Hemiptarsenus varicornis*	Philippines	Baucas *et al.* (2003)
Eulophidae	Eulophinae	*Hemiptarsenus varicornis* (Girault, 1913)	*Hemiptarsenus varicornis*	Sri Lanka	Nugaliyada (2000)
Eulophidae	Eulophinae	*Hemiptarsenus zilahisebessi* Erdös, 1951	*Hemiptarsenus zilahisebessi*	China	Song *et al.* (2003)
Eulophidae	Eulophinae	*Hemiptarsenus zilahisebessi* Erdös, 1951	*Hemiptarsenus zilahisebessi*	Israel	Weintraub *et al.* (2017)
Eulophidae	Eulophinae	*Hemiptarsenus zilahisebessi* Erdös, 1951	*Hemiptarsenus zilahisebessi*	Jordan	Al-Ghabeish & Allawi (2001)
Eulophidae	Eulophinae	*Meruacesa* sp.	*Meruacesa* sp.	Kenya	Foba *et al.* (2016)
Eulophidae	Eulophinae	*Pnigalio incompletus* (Bouček, 1971)	*Pnigalio incompletus*	Jordan	Al-Ghabeish & Allawi (2001)
Eulophidae	Eulophinae	*Pnigalio katonis* (Ishii, 1953)	*Pnigalio katonis*	China	Song *et al.* (2003)
Eulophidae	Eulophinae	*Pnigalio katonis* (Ishii, 1953)	*Pnigalio katonis*	Philippines	Baucas *et al.* (2003)
Eulophidae	Eulophinae	*Pnigalio soemius* (Walker, 1839)	*Pnigalio soemius*	Israel	Weintraub *et al.* (2017)
Eulophidae	Eulophinae	*Pnigalio* sp.	*Pnigalio* sp.	China	Pan *et al.* (2019)
Eulophidae	Eulophinae	*Pnigalio* sp.	*Pnigalio* sp.	Indonesia	Rauf & Shepard (2001)
Eulophidae	Eulophinae	*Zagrammosoma latilineatum* Ubaidillah, 2000	*Zagrammosoma latilineatum*	Indonesia	Ubaidillah *et al.* (2000)
Eulophidae	Eulophinae	*Zagrammosoma multilineatum* (Ashmead, 1888)	*Zagrammosoma multilineatum*	Peru	Weintraub *et al.* (2017)
Eulophidae	Eulophinae	*Zagrammosoma* sp.	*Zagrammosoma* sp.	Peru	Delgado & Aguilar (1980)
Eulophidae	Eulophinae	*Zagrammosoma* sp.	*Zagrammosoma* sp.	Philippines	Baucas *et al.* (2003)
Eulophidae	Tetrastichinae	*Quadrastichus liriomyzae* Hansson & LaSalle, 1996	*Quadrastichus liriomyzae*	Philippines	Baucas *et al.* (2003)
Eulophidae	Tetrastichinae	*Quadrastichus* sp.	*Quadrastichus* sp.	Guatemala	Weintraub *et al.* (2017)
Eulophidae	Tetrastichinae	*Quadrastichus* sp.	*Quadrastichus* sp.	Indonesia	Rauf & Shepard (2001)
Eulophidae	Tetrastichinae	*Quadrastichus* sp.	*Quadrastichus* sp.	Israel	Weintraub *et al.* (2017)
Figitidae	Eucoilinae	*Alloxysta* sp.	*Alloxysta* sp.	Easter Island	Ripa *et al.* (1995)
Figitidae	Eucoilinae	*Ganaspidium* sp.	*Ganaspidium* sp.	Peru	Redolfi *et al.* (1985); Sánchez & Redolfi (1985); Iannacone (1998); Weintraub *et al.* (2017)

Table S2 Parasitoid species reared from *Liriomyza huidobrensis*

Family	Sub-family	Full Name	Parasitoid name in text	Location	Reference
Figitidae	Eucoilinae	*Gronotoma adachiae* Beardsley, 1988	*Gronotoma adachiae*	China	Abe & Konishi (2012)
Figitidae	Eucoilinae	*Gronotoma micromorpha* (Perkins, 1910)	*Gronotoma micromorpha*	Indonesia	Prijono *et al.* (2004); Tantowijoyo & Hoffmann (2010); La Daha (2011)
Figitidae	Eucoilinae	*Gronotoma* sp.	*Gronotoma sp*	China	Pan *et al.* (2019)
Figitidae	Eucoilinae	*Gronotoma* sp.	*Gronotoma* sp.	Guatemala	MacVean & Perez (1997)
Figitidae	Eucoilinae	*Gronotoma* sp.	*Gronotoma* sp.	Jordan	Al-Ghabeish & Allawi (2001)
Figitidae	Eucoilinae	*Moneucoela* sp.	*Moneucoela* sp.	Guatemala	MacVean & Perez (1997)
Figitidae	Eucoilinae	*Sinatra pacifica*	*Disorygma pacifica*	Guatemala	MacVean & Perez (1997)
Figitidae	Eucoilinae	*Tribliographa* sp.	*Tribliographa* sp.	Columbia	Andrade *et al.* (1989)
Figitidae	Eucoilinae	*Zaeucoila clavatus* (Diaz, 1976)	*Agrostocynips clavatus*	Argentina	Salvo & Valladares (1995); Salvo *et al.* (2005); Videla *et al.* (2006)
Figitidae	Eucoilinae	*Zaeucolia* sp.	*Zaeucolia* sp.	Guatemala	Weintraub *et al.* (2017)
Pteromalidae		Pteromalidae species	Pteromalidae species	Argentina	Salvo & Valladares (1995)
Pteromalidae		Pteromalidae species	Pteromalidae species	Israel	Weintraub *et al.* (2017)
Pteromalidae		Pteromalidae species	Pteromalidae species	Portugal	Mexia *et al.* (2004)
Pteromalidae	Miscogastrinae	*Halticoptera arduine* (Walker, 1843)	*Halticoptera arduine*	Argentina	de Santis (1987)
Pteromalidae	Miscogastrinae	*Halticoptera arduine* (Walker, 1843)	*Halticoptera arduine*	Argentina	Neder de Román (2000)
Pteromalidae	Miscogastrinae	*Halticoptera arduine* (Walker, 1843)	*Halticoptera arduine*	Chile	Neder de Román (2000)
Pteromalidae	Miscogastrinae	*Halticoptera arduine* (Walker, 1843)	*Halticoptera arduine*	Peru	Redolfi *et al.* (1985); Sanchez & Redolfi (1985); Iannacone (1998); Iannacone & Reyes (2001); Weintraub *et al.* (2017)
Pteromalidae	Miscogastrinae	*Halticoptera circulus* (Walker, 1833)	*Halticoptera circulus*	Austria	Stolz & Blümel (1998)
Pteromalidae	Miscogastrinae	*Halticoptera circulus* (Walker, 1833)	*Halticoptera circulus*	Canada	Bahlai *et al.* (2006)
Pteromalidae	Miscogastrinae	*Halticoptera circulus* (Walker, 1833)	*Halticoptera circulus*	China	Chen *et al.* (2000)
Pteromalidae	Miscogastrinae	*Halticoptera circulus* (Walker, 1833)	*Halticoptera circulus*	China	Song *et al.* (2003)
Pteromalidae	Miscogastrinae	*Halticoptera circulus* (Walker, 1833)	*Halticoptera circulus*	Guatemala	Weintraub *et al.* (2017)
Pteromalidae	Miscogastrinae	*Halticoptera circulus* (Walker, 1833)	*Halticoptera circulus*	Jordan	Al-Ghabeish & Allawi (2001)
Pteromalidae	Miscogastrinae	*Halticoptera helioponi* De Santis ,1976	*Halticoptera helioponi*	Argentina	Salvo & Valladares (1995); Salvo & Valladares (2002); Salvo *et al.* (2005); Videla *et al.* (2006)

Table S2 Parasitoid species reared from *Liriomyza huidobrensis*

Family	Sub-family	Full Name	Parasitoid name in text	Location	Reference
Pteromalidae	Miscogastrinae	*Halticoptera patellana* (Dalman, 1818)	*Halticoptera patellana*	Peru	Delgado & Aguilar (1980)
Pteromalidae	Miscogastrinae	*Halticoptera peruviana* De Santis, 1987	*Halticoptera peruviana*	Argentina	de Santis (1987)
Pteromalidae	Miscogastrinae	*Halticoptera* sp.	*Halticoptera* sp.	Argentina	Salvo *et al.* (2005)
Pteromalidae	Miscogastrinae	*Halticoptera* sp.	*Halticoptera* sp.	Columbia	Hincapie *et al.* (1993)
Pteromalidae	Miscogastrinae	*Halticoptera* sp.	*Halticoptera* sp.	Easter Island	Ripa *et al.* (1995)
Pteromalidae	Miscogastrinae	*Halticoptera* sp.	*Halticoptera* sp.	Guatemala	MacVean & Perez (1997)
Pteromalidae	Miscogastrinae	*Halticoptera* sp.	*Halticoptera* sp.	Jordan	Al-Ghabeish & Allawi (2001)
Pteromalidae	Miscogastrinae	*Halticoptera* sp.	*Halticoptera* sp.	Peru	Delgado & Aguilar (1980); Weintraub *et al.* (2017)
Pteromalidae	Miscogastrinae	*Halticoptera* sp.	*Halticoptera* sp.	Portugal	Mexia *et al.* (2004)
Pteromalidae	Miscogastrinae	*Thinodytes cyzicus* (Walker, 1839)	*Thinodytes cyzicus*	China	Chen *et al.* (2000)
Pteromalidae	Miscogastrinae	*Thinodytes* sp.	*Thinodytes* sp.	Argentina	Salvo *et al.* (2005)
Pteromalidae	Pteromalinae	*Heteroschema* sp.	*Heteroschema* sp.	Peru	Delgado & Aguilar (1980)
Pteromalidae	Pteromalinae	*Notoglyptus tzeltales* Heydon, 1988	*Notoglyptus tzeltales*	Guatemala	MacVean & Perez (1997)
Pteromalidae	Pteromalinae	*Sphegigaster* sp.	*Sphegigaster* sp.	Indonesia	Rauf & Shepard (2001)
Pteromalidae	Pteromalinae	*Trichomalopsis* sp.	*Trichomalopsis* sp.	China	Chen *et al.* (2000)
Tetracampidae	Platynocheilinae	*Platynocheilus cuprifrons* (Nees, 1834)	*Platynocheilus cuprifrons*	Israel	Weintraub *et al.* (2017)
Tetracampidae	Tetracampinae	*Epiclerus* sp.	*Epiclerus* sp.	Portugal	Mexia *et al.* (2004)

Table S2 Parasitoid species reared from *Liriomyza huidobrensis*

References

Abe Y & Konishi K. 2012. New distributional and host records for the parasitoid *Gronotoma adachiae* (Hymenoptera: Figitidae: Eucoilinae) in Asia. *Entomological Science* **15**, 346–348.

Al-Ghabeish I & Allawi TF. 2001. Agromyzid leaf miners and their parasitoids in Jordan. *Dirasat, Agricultural Sciences*, **28**, 172–177.

Andrade ME, Briceno JA, de Hoyos PM & Jimenez J. 1989. Busqueda y recononcimiento de los enemigos naturales y hospedantes alternos de las principales plagas. En flores bajo invernadero en la sabana de Bogato. *Acta Biológica Colombiana* **1**(5), 45–57.

Arce de Hamity MG & Neder de Román LE. 1984. Detección de los insectos dañinos y benéficos al cultivo de Vicia faba en zonas de altura. *Revista de la Sociedad Entomológica Argentina* **43**, 7-11.

Bahlai CA, Goodfellow SA, Stanley-Horn DE & Hallett RH. 2006. Endoparasitoid assemblage of the pea leafminer, *Liriomyza huidobrensis* (Diptera: Agromyzidae), in southern Ontario. *Environmental Entomology* **35**, 351–357.

Baucas NS, Joshi RC, Verzola EA & Sacla GL. 2003. Exploratory survey of leafminer flies (Diptera: Agromyzidae) and their parasitoids in the highlands of Cordillera, Philippines. *Journal of Agriculture & Life Sciences* **37**, 43–58.

Burgio G, Lanzoni A, Masetti A & Manucci F. 2005. Spatial patterns and sampling plan for *Liriomyza huidobrensis* (Diptera: Agromyzidae) and related parasitoids on lettuce. *Environmental Entomology* **34**, 178–183.

Calabretta C, Calabro M, Colombo A & Campo G. 1995. Diffusione di *Liriomyza huidobrensis* (Blanchard) (Diptera, Agromyzidae) in colture protette della Sicilia *Informatore Fitopatologico* **45**, 24–30.

Campos RG. 1978. Control quimico de la "Mosca Minadora" (*Liriomyza huidobrensis*) en el valle de Cañete. *Revista Peruana de Entomología* **21**, 105–108.

Carballo M, Leon GR & Ramirez A. 1990. Combate biologico de *Liriomyza* sp. (Diptera: Agromyzidae) en cultivos horticolas de Costa Rica. *Manejo Integrado de Plagas (Costa Rica)* **16**, 4–11.

Chen Z-Q, Luo K-J, Hua Q-J & Zhang Q. 2000. A preliminary study on the parasitic insects of *Liriomyza huidobrensis* in Yunnan Province. *Journal of Yunnan Agricultural University* **15**, 337–338.

Chen B, Wang JJ, Zhang LM, Liu, ZY & Xiao G-L. 2011. Effect of intercropping pepper with sugarcane on populations of *Liriomyza huidobrensis* (Diptera: Agromyzidae) and its parasitoids. *Crop Protection* **30**, 253-258.

Chien C-C & Chang S-C. 2012. Effect of insecticides on survival and fertility of *Liriomyza huidobrensis* (Diptera: Agromyzidae) and its parasitoid, *Opius caricivorae* (Hymenoptera: Braconidae). *Journal of Taiwan Agricultural Research* **61**, 316–329.

Chien C-C & Chang S-C. 2013. Effect of temperature on killing of *Liriomyza huidobrensis* (Diptera: Agromyzidae) by the parasitoids *Chrysocharis pentheus* and *Closterocerus okazakii* (Hymenoptera: Eulophidae). *Journal of Taiwan Agricultural Research* **62**, 71–82.

Civelek HS, Yoldas Z & Weintraub P. 2002. The parasitoid complex of *Liriomyza huidobrensis* in cucumber greenhouses in Izmir Province, western Turkey. *Phytoparasitica* **30,** 285–287.

Çıkman E, Beyarslan A & Civelek HS. 2006. Parasitoids of leafminers (Diptera: Agromyzidae) from southeast Turkey with 3 new records. *Turkish Journal of Zoology* **30**, 167–173.

Cure JR & Cantor F. 2003. Predacious and parasitic activity of *Diglyphus begini* (Ashm.) (Hymenoptera: Eulophidae) on *Liriomyza huidobrensis* (Blanch.) (Diptera: Agromyzidae), in *Gypsophila paniculata* L. *Neotropical Entomology* **32**, 85–89.

de Santis L. 1987. Las especies peruanas de *Halticoptera* (Insecta, Hymenoptera, Pteromalidae). *Revista Peruana de Entomología* **28**, 1–3.

Delgado JJ & Aguilar PG. 1980. Apuntes sobre el control biológico y el control integrado de las plagas agrícolas en el Perú. - V. El cultivo de la papa. *Revista Peruana de Entomología* **23**, 102–104.

Dequech STB, Sturza VS, Ribeiro LP, Sausen CD, Egewarth R, Milani M & Schirmann J. 2010. Inseticidas botânicos sobre *Liriomyza huidobrensis* Blanchard (Diptera: Agromyzidae) e seus parasitóides em feijão-de-vagem cultivado em estufa. *Biotemas* **23**(2), 37–43.

Fisher N & La Salle J. 2005. A new species of *Neochrysocharis* Kurdjumov (Hymenoptera: Eulophidae), a parasitoid of serpentine leafminers (Diptera: Agromyzidae) in southeast Asia. *Zootaxa* **1044**, 27–34.

Foba CN, Lagat ZO, Gitonga LM, Akutse KS & Fiaboe KKM. 2015. Interaction between *Phaedrotoma scabriventris* Nixon and *Opius dissitus* Muesebeck (Hymenoptera: Braconidae): endoparasitoids of Liriomyza Leafminer. *African Entomology* **23**, 120-131.

Foba CN, Salifu D, Lagat ZO, Gitonga LM, Akutse KS & Fiaboe KKM. 2016. *Liriomyza* leafminer (Diptera: Agromyzidae) parasitoid complex in different agroecological zones, seasons, and host plants in Kenya. *Environmental Entomology* **45**, 357–366.

Galantini LV & Redolfi I. 1992. Niveles de infestación y parasitismo de *Liriomyza huidobrensis* en papa cultivada sin aplicación de insecticida. *Revista Peruana de Entomologia* **35**, 101-106.

Godinho M & Mexia A. 2000. Leafminers (*Liriomyza* sp.) importance in greenhouses in the Oeste region of Portugal and its natural parasitoids as control agents in IPM programs. *International Organisation for Biological and Integrated Control /West Palaearctic Regional Section Bulletin* **23**(1), 157–161.

Grenouillet C, Martinez M & Rasplus JY. 1993. Liste des parasitoïdes et des prédateurs des *Liriomyza* d'importance economique dans le monde (Diptera: Agromyzidae). *Colloque sur les mouches mineuses des plantes cultivees*, 24-26 Mar 1993, Montpellier, France, pp. 143-156.

Guimarães JA, Filho MM, Oliveira VR, De Liz RS, & Araujo EL. 2009. Biologia e manejo de mosca minadora no meloeiro. *Circular Técnica* 77, EMBRAPA.

Guimarães JA, Oiveira VR, Michereff M & Liz RZ. 2010. Ocorrência da mosca minadora sul-americana e seus himenópteros parasitóides em meloeiro no Distrito Federal. *Horticultura Brasileira* **28**, S790-S794.

Hidalgo, J.E & V.M. Carballo. 1991. Influencia de las malezas sobre los insectos controladores naturales de *Liriomyza huidobrensis* (Blanchard), (Diptera:Agromyzidae). *Manejo Integrado de Plagas (Costa Rica)* **20–21**, 49–54.

Hidrayani, Purnomo, Rauf A, Ridland PM & Hoffmann AA. 2005. Pesticide applications on Java potato fields are ineffective in controlling leafminers, and have antagonistic effects on natural enemies of leafminers. *International Journal of Pest Management* **51**, 181–187.

Hincapie CMC, Saavedra HME & Trochez PAL. 1993. Life cycle, behavior and natural enemies of *Liriomyza huidobrensis* (Blanchard) on bulb onion (*Allium cepa* L.). *Revista Colombiana de Entomología* **19**, 51–57.

Iannacone JAO & Reyes MU. 2001. Efecto de la retenona y neem sobre *Bemisia tabaci* Gennadius (Homoptera: Aleyrodidae) y *Liriomyza huidobrensis* Blanchard (Diptera: Agromyzidae) plagas del tomate en el Peru. *Agronomia Tropical* **15**, 65–79.

Iannacone JAO. 1998. Diversidad de la fauna de parasitoides de la mosca minadora, *Liriomyza huidobrensis* (Diptera: Agromyzidae) en frijol cultivado en la zona de Lima, Perú. *Revista Colombiana de Entomología* **24**, 103–107.

La Daha. 2011. Parasitoid quality of *Gronotoma micromorpha* parasitizing *Liriomyza huidobrensis* on Chinese cabbage and soybean. *Hayati Journal of Biosciences* **18,** 113–117.

Luo K-J, Chen Z-Q & Chen A-D. 2000. Activity of *Diglyphus isaea* (Hymenoptera; Eulophidae) in broad bean fields. *Natural Enemies of Insects* 22, 68-71.

Luo K-J, Chen Z-Q, Hua Q-J & Chen A-D. 2001. Seasonal incidence of *Liriomyza huidobrensis* (Diptera: Agromyzidae) and its parasitoids on vegetables in Yunnan. *Plant Protection* **27**(3), 7–9.

MacVean C & Pérez R. 1997. A taxonomic survey of the snowpea leaf mining species (Agromyzidae) in the Guatemalan highlands. Virginia Tech IPM/CRSP, Final report. 8 pp. (cited in Weintraub et al. (2017)

Mexia A, Figueiredo E & do Céu Godinho M. 2004. Natural control against pests on vegetables in Portugal: important species and their role. *International Organisation for Biological and Integrated Control /West Palaearctic Regional Section Bulletin* **27**(6), 1–8.

Neder de Román LE. 2000. Análisis de la relación *Halticoptera arduine* (Walker) (Hym. Pteromalidae) - *Liriomyza huidobrensis* Blanchard (Dipt. Agromyzidae) en la Prepuna Argentina. *Idesia* **18**, 49–60.

Neder de Roman, L & Arce de Hamity M. 1984. Revision y nuevas aportes al conocimiento bioecologico de *Liriomyza huidobrensis* (Diptera, Agromyzidae). *Acta Zoológica Lilloana* **37**, 295–301.

Nor Ahya M & Nur Liyana I. 2018. Relationship of leaf miner populations with biotic and abiotic factors in tomato farms in Cameron Highlands. *Journal of Tropical Agriculture and Food Science* **46**, 107–115.

Noujeim E, Sakr J, & Nemer N. 2013. Potential of entomopathogenic nematodes application against *Liriomyza huidobrensis* Blanchard in Lebanon, pp. 692–698, *In Proceedings, 4th International Scientific Symposium "Agrosym 2013"*, Faculty of Agriculture, University of East Sarajevo. Bosnia and Herzegovina.

Noyes JS. 1994. The reliability of published host-parasitoid records: a taxonomist's view. *Norwegian Journal of Agricultural Sciences Supplement* **16**, 59–69.

Nugaliyada M. 2000. A new pest, leaf miner, *Liriomyza huidobrensis* (Blanchard) of potato in Sri Lanka. *South and West Asia International Potato Center Newsletter* **3(2)**, 1-4.

Olivera CR & Bordat D. 1996. Influence of *Liriomyza* species (Diptera: Agromyzidae) and their host plants, on oviposition by *Opius dissitus* females (Hymenoptera: Braconidae). *Annals of Applied Biology* **128**, 399–404.

Pan L-T, Xu Y-Q, Du S-J, Wang W, Yefremova Z, Dawa & Liu W-X. 2019. First record of *Liriomyza huidobrensis* (Diptera: Agromyzidae) and a survey of its parasitoids in Tibet, China. *Acta Entomologica Sinica* **62**, 1072–1080.

Pereira DIP, Souza JC, Santa-Cecília LVC, Reis PR & Souza MA. 2002. Parasitismo de larvas da mosca-minadora *Liriomyza huidobrensis* Blanchard (Diptera: Agromyzidae) pelo parasitóide *Opius* sp. (Hymenoptera: Braconidae) na cultura da batata com faixas de feijoeiro intercaladas. *Ciência e Agrotecnologia* **26**, 955–963.

Prijono D, Robinson M, Rauf A, Bjorksten T & Hoffmann AA. 2004. Toxicity of chemicals commonly used in Indonesian vegetable crops to *Liriomyza huidobrensis* populations and Indonesian parasitoids *Hemiptarsenus varicornis, Opius* sp., and *Gronotoma micromorpha,* as well as the Australian parasitoids *Hemiptarsenus varicornis* and *Diglyphus isaea*. *Journal of Economic Entomology* **97**, 1191–1197.

Rauf A, Shepard BM & Johnson MW (2000). Leafminers in vegetables, ornamental plants and weeds in Indonesia: surveys of host crops, species composition and parasitoids. *International Journal of Pest Management* 46, 257–266.

Redolfi I, Palacios M & Alcazar J. 1985. Hymenoptera parasitoides de *Liriomyza huidobrensis* en papa cultivada en Rímac, Canete e Ica. *Revista Peruana de Entomología* **28**, 19–21.

Ripa SR, Rojas PS & Velasco G. 1995. Releases of biological control agents of insect pests on Easter Island (Pacific Ocean). *Entomophaga* 40, 427–440.

Rodríguez CL. 1997. La investigacion en *Liriomyza huidobrensis* en el cultivo de pape en Cartago Costa Rica. *Manejo Integrado de Plagas,* (*Costa Rica)* **46**, 1–8.

Salvo A & Valladares G. 1995. Complejo parasítico (Hymenoptera: Parasitica) de *Liriomyza huidobrensis* (Diptera: Agromyzidae) en haba. *Agriscientia* **12**, 39–47.

Salvo A & Valladares G. 1997. An analysis of leaf-miner and plant host ranges of three *Chrysocharis* species (Chalcidoidea: Eulophidae) from Argentina. *Entomophaga*, **42**, 387–396.

Salvo A & Valladares G. 2002. Plant-related intraspecific size variation in parasitoids (Hymenoptera: Parasitica) of a polyphagous leafminer (Diptera: Agromyzidae). *Environmental Entomology* **31**, 874–879.

Salvo A, Fenoglio MS & Videla M. 2005. Parasitism of a leafminer in managed versus natural habitats. *Agriculture, Ecosystems and Environment* **109**, 213–220.

Sánchez GA & Redolfi I. 1985. Parasitoides de *Liriomyza huidobrensis* y *Scrobipalpula absoluta* en papa cultivada en Lima, 1984. *Revista Peruana de Entomologia* **28**, 31-33.

Shaw MR. 1994. Parasitoid host ranges. In: *Parasitoid community ecology.* (eds BA Hawkins & Sheehan W). pp 451–471. Oxford University Press, Oxford, UK.

Shepard BM, Samsudin & Braun AR. 1998. Seasonal incidence of *Liriomyza huidobrensis* (Diptera: Agromyzidae) and its parasitoids on vegetables in Indonesia. *International Journal of Pest Management* **44**, 43–47.

Shindo, J-I, Kinota M, Inokuchi S, Kimura Y & Fujimura T. 2005. Occurrence of pea leafminer, *Liromyza huidobrensis* (Blanchard) (Diptera: Agromyzidae), in Aomori Prefecture. Ann. Rep. Plant Prot. North Japan 56: 145–148.

Sivapragasam, A., Syed, AR, LaSalle J, Ruwaida, M. 1999. Parasitoids of invasive agromyzid leafminers on vegetables in peninsular Malaysia, pp 8–19. In *Proceedings of symposium on Biological Control in the Tropics, 18–19 March*. MARDI Training Centre, Malaysia.

Song L, Xu Z & Gu D. 2003. A review on the parasitoids of *Liriomyza huidobrensis* Blanchard. *Natural Enemies of Insects* **25**, 37–41.

Stolz M & Blümel S. 1998, Occurrence of agromyzid leafminer parasitoids in three green-houses with different ornamental crops in Austria. *Zeitschrift für Pflanzenkrankheiten und Pflanzenschutz* **105**, 71–77.

Tantowijoyo W & Hoffmann AA. 2010. Identifying factors determining the altitudinal distribution of the invasive pest leafminers *Liriomyza huidobrensis* and *Liriomyza sativae*. *Entomologia Experimentalis Applicata* **135**, 141–153.

Ubaidillah R, LaSalle J & Rauf A. 2000. A new species of *Zagrammosoma* (Hymenoptera: Eulophidae) from the Indo-Australian region, a parasitoid of the invasive pest species *Liriomyza huidobrensis* (Diptera: Agromyzidae). *Oriental Insects* **34**, 221–228.

van Achterberg C & Salvo A. 1997. Reared Opiinae (Hymenoptera: Braconidae) from Argentina. *Zoologische Mededelingen* **71**, 189–214.

van der Linden, A. 1991. Prospects for the biological control of *Liriomyza huidobrensis* (Blanchard) in Dutch glasshouse tomatoes. *Mededelingen van de Faculteit Landbouwwetenschappen Rijksuniversiteit Gen*t **56** (2a), 265–271.

van der Linden, A. 1990. Prospects for the biological control of *Liriomyza huidobrensis* (Blanchard), a new leafminer for Europe. *International Organisation for Biological and Integrated Control /West Palaearctic Regional Section Bulletin* **13**(5), 100–103.

Vayssieres, J. F., G. Delvare, J.M. Maldes, and H.P. Aberlenc. 2001. Auxilliaires des cultures maraicheres sur l'Ile de la Reunion. *Insect Science and Its Application* **21**, 1–22.

Videla, M., G. Valladares, and A. Salvo. 2006. A tritrophic analysis of host preference and performance in a polyphagous leafminer. *Entomologia Experimentalis et Applicata* **121**, 105–114.

Visser D & Weintraub P. 2001. Outbreak of the potato leafminer, comparisons between Israel and South Africa. *Chips* **15**(2), 38–39.

Weintraub PG & Horowitz AR. 1996. Spatial and diel activity of the pea leafminer (Diptera: Agromyzidae) in potatoes, *Solanum tuberosum*. *Environmental Entomology* **25**, 722–726.

Weintraub PG. 1999. Effects of cryomazine and abamectin on the leafminer, *Liriomyza huidobrensis* and its parasitoid, *Diglyphus isaea* in celery. *Annals of Applied Biology* **135**, 547–554.

Weintraub PG, Scheffer SJ, Visser D, Valladares G, Correa AS, Shepard BM, Rauf A, Murphy ST, Mujica N, MacVean C, Kroschel J, Kishinevsky M, Joshi RC, Johansen NS, Hallett RH, Çivelek HS, Chen B & Metzler HB (2017). The invasive *Liriomyza huidobrensis* (Diptera: Agromyzidae): understanding its pest status and management globally. *Journal of Insect Science* **17(1)**, 28: 1–27. doi: 10.1093/jisesa/iew121

Table S3 Parasitoid species reared from *Liriomyza sativae*

Specimens reared from mixed collections of *Liriomyza* spp., which included *L. sativae*, were not included in the list. This table is based on data from Ridland *et al.* (2020) with supplementary data added. As with any compilation of species names from the scientific literature, there will be uncertainty about the validity of some species and sampling intensity varies dramatically between countries, which usually reflects the scientific input into the problem rather than the actual size of the problem (Grenouillet *et al.* 1993; Noyes 1994; Shaw 1994). Given the nomenclatural changes that have occurred in time, the currently accepted name is given, together with the name used in the original reference. All references for the Appendix are appended.

Family	Sub-family	Full Name	Name in Reference	Location	Reference
Eulophidae	Entedoninae	*Achrysocharoides zwoelferi* Delucchi, 1954	*Achrysocharoides zwoelferi*	USA	Gates *et al.* (2002)
Eulophidae	Entedoninae	*Asecodes delucchii* (Bouček, 1971)	*Asecodes deluchii*	Indonesia	Tantowijoyo & Hoffmann (2010); Herlinadewi et al. (2013)
Eulophidae	Entedoninae	*Asecodes delucchii* (Bouček, 1971)	*Asecodes deluchii*	Japan	Amano *et al.* (2008)
Eulophidae	Entedoninae	*Asecodes delucchii* (Bouček, 1971)	*Asecodes deluchii*	Vietnam	Tran (2009)
Eulophidae	Entedoninae	*Asecodes erxias* (Walker, 1848)	*Asecodes erxias*	Japan	Amano *et al.* (2008)
Eulophidae	Entedoninae	*Chrysocharis ainsliei* Crawford, 1912	*Chrysocharis ainsliei*	USA	Johnson *et al.* (1980); Chandler (1982)
Eulophidae	Entedoninae	*Chrysocharis ainsliei* Crawford, 1912	*Chrysocharis ainsliei*	USA	Oatman (1959)
Eulophidae	Entedoninae	*Chrysocharis caribea* Bouček, 1977	*Chrysocharis caribea*	Barbados	Bouček (1977)
Eulophidae	Entedoninae	*Chrysocharis caribea* Bouček, 1977	*Chrysocharis caribea*	Peru	Mujica & Kroschel (2011)
Eulophidae	Entedoninae	*Chrysocharis caribea* Bouček, 1977	*Chrysocharis caribea*	Trinidad	Bouček (1977)
Eulophidae	Entedoninae	*Chrysocharis caribea* Bouček, 1977	*Chrysocharis caribea*	Venezuela	Bouček (1977)
Eulophidae	Entedoninae	*Chrysocharis crassiscapus* (Thomson, 1878)	*Chrysocharis mallochi*	Canada	McClanahan (1977)
Eulophidae	Entedoninae	*Chrysocharis flacilla* (Walker, 1842)	*Chrysocharis flacilla*	Peru	Mujica & Kroschel (2011)
Eulophidae	Entedoninae	*Chrysocharis giraulti* Yoshimoto, 1973	*Chrysocharis giraulti*	USA	Oatman & Johnson (1981)
Eulophidae	Entedoninae	*Chrysocharis oscinidis* Ashmead, 1888	*Chrysocharis viridis*	Canada	McClanahan (1977)
Eulophidae	Entedoninae	*Chrysocharis oscinidis* Ashmead, 1888	*Chrysocharis oscinidis*	China	Xu *et al.* (1999)
Eulophidae	Entedoninae	*Chrysocharis oscinidis* Ashmead, 1888	*Chrysocharis parksi*	USA	Johnson *et al.* (1980); Johnson (1987)
Eulophidae	Entedoninae	*Chrysocharis oscinidis* Ashmead, 1888	*Chrysocharis parksi*	USA	Oatman (1959)
Eulophidae	Entedoninae	*Chrysocharis pentheus* (Walker, 1839)	*Chrysocharis pentheus*	Bangladesh	Mazumdar & Bhuiya (2016)
Eulophidae	Entedoninae	*Chrysocharis pentheus* (Walker, 1839)	*Chrysocharis pentheus*	China	Xu *et al.* (1999)
Eulophidae	Entedoninae	*Chrysocharis pentheus* (Walker, 1839)	*Chrysocharis pentheus*	Japan	Amano *et al.* (2008)
Eulophidae	Entedoninae	*Chrysocharis pentheus* (Walker, 1839)	*Chrysocharis pentheus*	Taiwan	Chien & Chang (2011)

Table S3 Parasitoid species reared from *Liriomyza sativae*

Family	Sub-family	Full Name	Name in Reference	Location	Reference
Eulophidae	Entedoninae	*Chrysocharis pentheus* (Walker, 1839)	*Chrysocharis pentheus*	Vietnam	Tran (2009)
Eulophidae	Entedoninae	*Chrysocharis vonones* (Walker, 1839)	*Chrysocharis vonones*	Brazil	Costa-Lima *et al.* (2014)
Eulophidae	Entedoninae	*Chrysocharis vonones* (Walker, 1839)	*Chrysocharis brethesi*	Peru	Mujica & Kroschel (2011)
Eulophidae	Entedoninae	*Chrysonotomyia reticulata* Zheng & Zhan 2000	*Chrysonotomyia reticulata*	China	Sheng & Zhan (2000)
Eulophidae	Entedoninae	*Closterocerus cincinnatus* Girault, 1916	*Closterocerus cincinnatus*	USA	Gates *et al.* (2002)
Eulophidae	Entedoninae	*Closterocerus cinctipennis* Ashmead, 1888	*Closterocerus cinctipennis*	USA	Harding (1965), Stegmaier (1972)
Eulophidae	Entedoninae	*Closterocerus purpureus* (Howard, 1897)	*Chrysonotomyia purpurea*	Trinidad	Bouček (1977)
Eulophidae	Entedoninae	*Closterocerus trifasciatus* Westwood, 1833	*Closterocerus trifasciatus*	USA	Oatman (1959)
Eulophidae	Entedoninae	*Closterocerus utahensis* Crawford, 1912	*Closterocerus utahensis*	USA	Johnson *et al.* (1980a), Gates *et al.* (2002)
Eulophidae	Entedoninae	*Neochrysocharis agromyzae* (Crawford, 1913)	*Achrysocharella agromyzae*	USA	Harding (1965), Stegmaier (1972)
Eulophidae	Entedoninae	*Neochrysocharis arizonensis* (Crawford, 1913)	*Neochrysocharis arizonensis*	USA	Gates *et al.* (2002)
Eulophidae	Entedoninae	*Neochrysocharis arizonensis* (Crawford, 1913)	*Derostenus arizonensis*	USA	Oatman (1959)
Eulophidae	Entedoninae	*Neochrysocharis beasleyi* Fisher & La Salle, 1985	*Neochrysocharis beasleyi*	Vietnam	Tran (2009)
Eulophidae	Entedoninae	*Neochrysocharis diastatae* (Howard, 1881)	*Chrysonotomyia punctiventris*	China	Xu *et al.* (1999)
Eulophidae	Entedoninae	*Neochrysocharis diastatae* (Howard, 1881)	*Achrysocharella diastatae*	USA	Stegmaier (1972)
Eulophidae	Entedoninae	*Neochrysocharis diastatae* (Howard, 1881)	*Chrysonotomyia punctiventris*	USA	Johnson *et al.* (1980a), Johnson (1987)
Eulophidae	Entedoninae	*Neochrysocharis formosa* (Westwood, 1833)	*Neochrysocharis formosa*	Bangladesh	Mazumdar & Bhuiya (2016)
Eulophidae	Entedoninae	*Neochrysocharis formosa* (Westwood, 1833)	*Chrysonotomyia formosus*	China	Xu *et al.* (1999)
Eulophidae	Entedoninae	*Neochrysocharis formosa* (Westwood, 1833)	*Neochrysocharis formosa*	Indonesia	Tantowijoyo & Hoffmann (2010); Herlinadewi et al. (2013)
Eulophidae	Entedoninae	*Neochrysocharis formosa* (Westwood, 1833)	*Closterocerus formosus*	Iran	Asadi *et al.* (2006); Shareki *et al.* (2016)
Eulophidae	Entedoninae	*Neochrysocharis formosa* (Westwood, 1833)	*Neochrysocharis formosa*	Japan	Amano *et al.* (2008)
Eulophidae	Entedoninae	*Neochrysocharis formosa* (Westwood, 1833)	*Achrysocharella fullowayi*	USA	Harding (1965)
Eulophidae	Entedoninae	*Neochrysocharis formosa* (Westwood, 1833)	*Achrysocharella variipes*	USA	Harding (1965); Stegmaier (1972)

Table S3 Parasitoid species reared from *Liriomyza sativae*

**Family**	**Sub-family**	**Full Name**	**Name in Reference**	**Location**	**Reference**
Eulophidae	Entedoninae	*Neochrysocharis formosa* (Westwood, 1833)	*Chrysonotomyia formosus*	USA	Lema & Poe (1979); Johnson (1987)
Eulophidae	Entedoninae	*Neochrysocharis formosa* (Westwood, 1833)	*Chrysonotomyia formosa*	USA	Tryon & Poe (1981)
Eulophidae	Entedoninae	*Neochrysocharis formosa* (Westwood, 1833)	*Derostenus variipes*	USA	Oatman (1959)
Eulophidae	Entedoninae	*Neochrysocharis formosa* (Westwood, 1833)	*Neochrysocharis formosa*	Vietnam	Tran (2009)
Eulophidae	Entedoninae	*Neochrysocharis formosa* (Westwood, 1833)	*Neochrysocharis formosa*	Indonesia	Herlinadewi *et al.* (2013)
Eulophidae	Entedoninae	*Neochrysocharis okazakii* Kamijo, 1978	*Neochrysocharis okazakii*	Taiwan	Chien & Chang (2011)
Eulophidae	Entedoninae	*Neochrysocharis okazakii* Kamijo, 1978	*Neochrysocharis okazakii*	Vietnam	Tran (2009)
Eulophidae	Entedoninae	*Proacrias thysanoides* (De Santis, 1972)	*Proacrias thysanoides*	Peru	Mujica & Kroschel (2011)
Eulophidae	Eulophinae	*Cirrospilus brevicorpus* Shafee & Rizvi, 1988	*Cirrospilus ambiguus*	Malaysia	Sivapragasam et al. (1999)
Eulophidae	Eulophinae	*Cirrospilus brevicorpus* Shafee & Rizvi, 1988	*Cirrospilus ambiguus*	Vietnam	Tran (2009)
Eulophidae	Eulophinae	*Burkseus vittatus* (Walker, 1838)	*Cirrospilus vittatus*	Iran	Asadi *et al.* (2006)
Eulophidae	Eulophinae	*Diaulinopsis arenaria* (Erdös, 1951)	*Diaulinopsis arenaria*	China	Shi *et al.* (2000)
Eulophidae	Eulophinae	*Diaulinopsis callichroma* Crawford, 1912	*Diaulinopsis callichroma*	Peru	Mujica & Kroschel (2011)
Eulophidae	Eulophinae	*Diaulinopsis callichroma* Crawford, 1912	*Diaulinopsis callichroma*	USA	Oatman (1959), Stegmaier (1972), Gates *et al.* (2002)
Eulophidae	Eulophinae	*Diglyphus albiscapus* Erdös, 1951	*Diglyphus albiscapus*	Japan	Amano *et al.* (2008)
Eulophidae	Eulophinae	*Diglyphus begini* (Ashmead, 1904)	*Diglyphus begini*	Canada	McClanahan (1975, 1977)
Eulophidae	Eulophinae	*Diglyphus begini* (Ashmead, 1904)	*Diglyphus begini*	Colombia	de la Cruz *et al.* (1989)
Eulophidae	Eulophinae	*Diglyphus begini* (Ashmead, 1904)	*Diglyphus begini*	USA	Trumble & Nakakihara (1983), Heinz & Parrella (1990)
Eulophidae	Eulophinae	*Diglyphus begini* (Ashmead, 1904)	*Solenotus begini*	USA	Oatman (1959)
Eulophidae	Eulophinae	*Diglyphus chabrias* (Walker, 1838)	*Diglyphus chabrias*	Turkey	Yefremova *et al.* (2011)
Eulophidae	Eulophinae	*Diglyphus crassinervis* Erdös, 1958	*Diglyphus crassinervis*	Iran	Talebi *et al.* (2011)
Eulophidae	Eulophinae	*Diglyphus crassinervis* Erdös, 1958	*Diglyphus crassinervis*	Turkey	Yefremova *et al.* (2011)
Eulophidae	Eulophinae	*Diglyphus intermedius* (Girault, 1916)	*Diglyphus intermedius*	USA	Johnson *et al.* (1980a), Tryon & Poe (1981), Trumble & Nakakihara (1983)
Eulophidae	Eulophinae	*Diglyphus isaea* (Walker, 1838)	*Diglyphus isaea*	China	Xu *et al.* (1999)
Eulophidae	Eulophinae	*Diglyphus isaea* (Walker, 1838)	*Diglyphus isaea*	Iran	Asadi *et al.* (2006), Fathipour *et al.* (2006)

Table S3 Parasitoid species reared from *Liriomyza sativae*

**Family**	**Sub-family**	**Full Name**	**Name in Reference**	**Location**	**Reference**
Eulophidae	Eulophinae	*Diglyphus isaea* (Walker, 1838)	*Diglyphus isaea*	Japan	Amano *et al.* (2008)
Eulophidae	Eulophinae	*Diglyphus isaea* (Walker, 1838)	*Diglyphus isaea*	Turkey	Yefremova *et al.* (2011)
Eulophidae	Eulophinae	*Diglyphus isaea* (Walker, 1838)	*Diglyphus isaea*	Vietnam	Tran *et al.* (2005)
Eulophidae	Eulophinae	*Diglyphus minoeus* (Walker, 1838)	*Diglyphus minoeus*	Japan	Amano *et al.* (2008)
Eulophidae	Eulophinae	*Diglyphus pulchripes* (Crawford, 1912)	*Diglyphus pulchripes*	Canada	McClanahan (1977), Stegmaier (1972)
Eulophidae	Eulophinae	*Diglyphus pulchripes* (Crawford, 1912)	*Solenotus pulchripes*	USA	Oatman (1959)
Eulophidae	Eulophinae	*Diglyphus pusztensis* (Erdös & Novicky, 1951)	*Diglyphus pusztensis*	Japan	Amano *et al.* (2008)
Eulophidae	Eulophinae	*Diglyphus sensilis* Yefremova, 2011	*Diglyphus sensilis*	Turkey	Yefremova *et al.* (2011)
Eulophidae	Eulophinae	*Diglyphus wani* Liu, Zhu & Yefremova, 2018	*Diglyphus wani*	China	Ye *et al.* (2018)
Eulophidae	Eulophinae	*Diglyphus websteri* (Crawford, 1912)	*Diglyphus websteri*	Argentina	Valladares *et al.* (1999)
Eulophidae	Eulophinae	*Diglyphus websteri* (Crawford, 1912)	*Diglyphus websteri*	Peru	Mujica & Kroschel (2011)
Eulophidae	Eulophinae	*Diglyphus websteri* (Crawford, 1912)	*Solenotus websteri; Diglyphus websteri*	USA	Oatman (1959); Harding (1965)
Eulophidae	Eulophinae	*Hemiptarsenus varicornis* (Girault, 1913)	*Hemiptarsenus varicornis*	Australia	Elia Pirtle (unpublished data)
Eulophidae	Eulophinae	*Hemiptarsenus varicornis* (Girault, 1913)	*Hemiptarsenus varicornis*	China	Xu *et al.* (1999)
Eulophidae	Eulophinae	*Hemiptarsenus varicornis* (Girault, 1913)	*Hemiptarsenus semialbiclavus*	Guam	Johnson (1993)
Eulophidae	Eulophinae	*Hemiptarsenus varicornis* (Girault, 1913)	*Hemiptarsenus varicornis*	Indonesia	Rauf *et al.* (2000); Herlinadewi et al. (2013)
Eulophidae	Eulophinae	*Hemiptarsenus varicornis* (Girault, 1913)	*Hemiptarsenus varicornis*	Japan	Amano *et al.* (2008)
Eulophidae	Eulophinae	*Hemiptarsenus varicornis* (Girault, 1913)	*Hemiptarsenus varicornis*	Sri Lanka	Nagalingam *et al.* (2007)
Eulophidae	Eulophinae	*Hemiptarsenus varicornis* (Girault, 1913)	*Hemiptarsenus varicornis*	Taiwan	Chien & Chang (2007)
Eulophidae	Eulophinae	*Hemiptarsenus varicornis* (Girault, 1913)	*Hemiptarsenus semialbiclavus*	USA	Johnson (1987)
Eulophidae	Eulophinae	*Hemiptarsenus varicornis* (Girault, 1913)	*Hemiptarsenus varicornis*	Vietnam	Tran (2009)
Eulophidae	Eulophinae	*Hemiptarsenus waiIeseIIae* Nowicki, 1929	*Hemiptarsenus waiIeseIIae*	Iran	Talebi *et al.* (2011)
Eulophidae	Eulophinae	*Hemiptarsenus zilahisebessi* Erdös, 1951	*Hemiptarsenus zilahisebessi*	Iran	Talebi *et al.* (2011), Shareki *et al.* (2016)
Eulophidae	Eulophinae	*Meruacesa liriomyzae* (Bouček, 1988)	*Meruana liriomyzae*	Mauritius	Bouček (1988)
Eulophidae	Eulophinae	*Meruacesa liriomyzae* (Bouček, 1988)	*Meruana liriomyzae*	Réunion	Bouček (1988)
Eulophidae	Eulophinae	*Pnigalio minio* (Walker, 1847)	*Pnigalio flavipes*	Canada	McClanahan (1977)
Eulophidae	Eulophinae	*Zagrammosoma americanum* Girault, 1916	*Zagrammosoma americanum*	Canada	McClanahan (1977)

Table S3   Parasitoid species reared from *Liriomyza sativae*

**Family**	**Sub-family**	**Full Name**	**Name in Reference**	**Location**	**Reference**
Eulophidae	Eulophinae	*Zagrammosoma americanum* Girault, 1916	*Zagrammosoma americanum*	USA	Chandler (1982)
Eulophidae	Eulophinae	*Zagrammosoma mirum* Girault, 1916	*Zagrammosoma mirum*	USA	Oatman (1959)
Eulophidae	Eulophinae	*Zagrammosoma multilineatum* (Ashmead, 1888)	*Zagrammosoma multilineatum*	USA	Stegmaier (1972)
Eulophidae	Eulophinae	*Zagrammosoma variegatum* (Nasi, 1907)	*Zagrammosoma variegatum*	Iran	Hesami *et al.* (2006)
Pteromalidae	Miscogastrinae	*Halticoptera aenea* (Walker, 1833)	*Halticoptera aenea*	USA	Oatman (1959); Palumbo *et al.* (1994)
Pteromalidae	Miscogastrinae	*Halticoptera arduine* (Walker, 1833)	*Halticoptera arduine*	Peru	Mujica & Kroschel (2011)
Pteromalidae	Miscogastrinae	*Halticoptera circulus* (Walker, 1833)	*Halticoptera circulus*	Guatemala	Rodríguez-Castañeda *et al.* (2017)
Pteromalidae	Miscogastrinae	*Halticoptera circulus* (Walker, 1833)	*Halticoptera circulus*	USA	Stegmaier (1972), Oatman & Johnson (1981)
Pteromalidae	Miscogastrinae	*Halticoptera patellana* (Dalman, 1918)	*Halticoptera patellana*	Canada	McClanahan (1977)
Pteromalidae	Miscogastrinae	*Halticoptera patellana* (Dalman, 1918)	*Halticoptera patellana*	USA	Jensen & Koehler (1970), Stegmaier (1972)
Pteromalidae	Pteromalinae	*Cyrtogaster vulgaris* Walker, 1833	*Cyrtogaster vulgaris*	Iran	Lotfalizadeh & Gharali (2008)
Figitidae	Eucoilinae	*Banacuniculus utilis* (Beardsley, 1988)	*Ganaspidium hunteri*	USA	Johnson (1987)
Figitidae	Eucoilinae	*Ganaspidium pusillae* Weld, 1955	*Ganaspidium pusillae*	USA	Harding (1965)
Figitidae	Eucoilinae	*Gronotoma adachiae* Beardsley, 1988	*Gronotoma adachiae*	Vietnam	Abe & Konishi (2012)
Figitidae	Eucoilinae	*Gronotoma micromorpha* (Perkins, 1910)	*Gronotoma micromorpha*	Indonesia	Herlinadewi et al. (2013)
Figitidae	Eucoilinae	*Sinatra pacifica* (Yoshimoto, 1962)	*Cothonaspis pacifica*	USA	Johnson (1987)
Braconidae	Opiinae	*Desmiostoma parvulum* (Wesmael 1835)	*Desmiostoma parvulum*	USA	Marsh (1979
Braconidae	Opiinae	*Oenongastra microrhopalae* (Ashmead, 1896)	*Oenongastra microrhopalae*	Canada	McClanahan (1977)
Braconidae	Opiinae	*Opius aridis* Gahan 1913	*Opius aridis*	USA	Harding (1965)
Braconidae	Opiinae	*Opius bruneipes* Gahan, 1913	*Opius bruneipes*	USA	Harding (1965)
Braconidae	Opiinae	*Opius caricivorae* Fischer, 1964	*Opius caricivorae*	China	Chen *et al.* (2003)
Braconidae	Opiinae	*Opius caricivorae* Fischer, 1964	*Opius caricivorae*	Taiwan	Chien & Chang (2012)
Braconidae	Opiinae	*Opius chromatomyiae* Belokobylskij & Wharton, 2004	*Opius chromatomyiae*	Indonesia	Wayhuni *et al.* (2017)
Braconidae	Opiinae	*Opius chromatomyiae* Belokobylskij & Wharton, 2004	*Opius chromatomyiae*	Vietnam	Tran (2009)
Braconidae	Opiinae	*Opius dimidiatus* (Ashmead, 1889)	*Opius dimidiatus*	Canada	McClanahan (1975, 1977)
Braconidae	Opiinae	*Opius dimidiatus* (Ashmead, 1889)	*Opius dimidiatus*	China	Xu *et al.* (1999)

Table S3 Parasitoid species reared from *Liriomyza sativae*

Family	Sub-family	Full Name	Name in Reference	Location	Reference
Braconidae	Opiinae	*Opius dimidiatus* (Ashmead, 1889)	*Opius dimidiatus*	USA	Harding (1965); Lema & Poe (1979)
Braconidae	Opiinae	*Opius dissitus* Muesebeck, 1963	*Opius dissitus*	China	Xu *et al.* (1999)
Braconidae	Opiinae	*Opius dissitus* Muesebeck, 1963	*Opius dissitus*	Guatemala	Rodríguez-Castañeda *et al.* (2017)
Braconidae	Opiinae	*Opius dissitus* Muesebeck, 1963	*Opius dissitus*	USA	Stegmaier (1972), Johnson (1987)
Braconidae	Opiinae	*Opius liriomyzae* Fischer, 1964	*Opius liriomyzae*	USA	Marsh (1979)
Braconidae	Opiinae	*Opius (Phaedrotoma) biroi* (Fischer, 1960)	*Phaedrotoma biroi*	China	Xing *et al.* (2017)
Braconidae	Opiinae	*Opius (Phaedrotoma) scabriventris* (Nixon, 1955)	*Opius (Gastrosema) scabriventris*	Brazil	Costa-Lima *et al.* (2014)
Braconidae	Opiinae	*Opius (Phaedrotoma) suturalis* (Gahan, 1913)	*Opius suturalis*	USA	Oatman (1959); Harding (1965)

Table S3 Parasitoid species reared from *Liriomyza sativae*

References

Abe Y & Konishi K. 2012. New distributional and host records for the parasitoid *Gronotoma adachiae* (Hymenoptera: Figitidae: Eucoilinae) in Asia. *Entomological Science* **15**, 346–348.

Amano K, Suzuki A, Hiromori H & Saito T. 2008. Relative abundance of parasitoids reared during field exposure of sentinel larvae of the leafminers *Liriomyza trifolii* (Burgess), *L. sativae* Blanchard, and *Chromatomyia horticola* (Goureau) (Diptera: Agromyzidae). *Applied Entomology and Zoology* **43**, 625–630.

Asadi R, Talebi AA, Fathipour Y, Moharramipour S & Rakhshani E. 2006. Identification of parasitoids and seasonal parasitism of the agromyzid leafminers genus *Liriomyza* (Dip.: Agromyzidae) in Varamin, Iran. *Journal of Agricultural Science and Technology* **8**, 293–303.

Bouček Z. 1977. Descriptions of two new species of Neotropical Eulophidae (Hymenoptera) of economic interest, with taxonomic notes on related species and genera. *Bulletin of Entomological Research* **67**, 1–15.

Bouček Z. 1988. *Australasian Chalcidoidea (Hymenoptera). A biosystematic revision of genera of fourteen families, with a reclassification of species*. CAB International, Wallingford, UK, 832 pp.

Chandler LD (1982). Parasitization of cantaloupe infesting agromyzid leafminers in the Lower Rio Grande Valley, Texas. *Southwestern Entomologist* **7**, 94–97.

Chen X-X, Lang F-Y, Xu Z-H, He J-H & Ma Y 2003. The occurrence of leafminers and their parasitoids on vegetables and weeds in Hangzhou area, Southeast China. *Biological Control* **48,** 515–527.

Chien C-C & Chang S-C 2007. Morphology, life history and life table of *Liriomyza sativae* (Diptera: Agromyzidae). *Formosan Entomologist* **27**, 207–227. (in Chinese with English abstract).

Chien C-C & Chang S-C 2011. Effect of insecticides on *Closterocerus okazakii* and *Chrysocharis pentheus* (Hymenoptera: Eulophidae), parasitoids of *Liriomyza sativae* (Diptera: Agromyzidae). *Journal of Taiwan Agricultural Research* **60**, 185–196 (in Chinese with English abstract).

Chien C-C & Chang S-C 2012. Morphology and life history of *Opius caricivorae* (Hymenoptera: Braconidae). *Journal of Taiwan Agricultural Research* **61**, 144–157 (in Chinese with English abstract).

Costa-Lima TC, Chagas MCM & Parra JRP. 2014. Temperature-dependent development of two neotropical parasitoids of *Liriomyza sativae* (Diptera: Agromyzidae). *Journal of Insect Science* **14**, 245, https://doi.org/10.1093/jisesa/ieu107.

de la Cruz AMR, Cardona CJ & de la Cruz JL. 1989. Ciclo de vida, habitos y enemigos naturales de *Liriomyza sativae* Blanchard (Diptera: Agromyzidae), minador del frijol. *Acta Agonomica* **39**, 133-141.

Fathipour Y, Haghani M, Talebi AA, Baniameri V, Zamani AA 2006. Natural parasitism of *Liriomyza sativae* (Diptera: Agromyzidae) on cucumber in field and greenhouse conditions. *IOBC/WPRS Bulletin* **29** (4), 155–160.

Gates MW, Heraty JM, Schauff ME, Wagner DL, Whitfield JB & Wahl DB 2002. Survey of the parasitic Hymenoptera on leafminers in California. *Journal of Hymenoptera Research* **11**, 213–270.

Grenouillet C, Martinez M & Rasplus JY. 1993. Liste des parasitoïdes et des prédateurs des *Liriomyza* d'importance economique dans le monde (Diptera: Agromyzidae). *Colloque sur les mouches mineuses des plantes cultivees*, 24-26 Mar 1993, Montpellier, France, 143-156.

Harding JA 1965. Parasitism of the leaf miner *Liriomyza munda* in the Winter Garden area of Texas. *Journal of Economic Entomology* **58**, 442–443.

Heinz KM & Parrella MP 1990. Holarctic distribution of the leafminer parasitoid *Diglyphus begini* (Ashmead) (Hymenoptera: Eulophidae) and notes on its life history attacking *Liriomyza trifolii* (Burgess) (Diptera: Agromyzidae) in chrysanthemum. *Annals of the Entomological Society of America* **83**, 916–924.

Herlinadewi NMS, Supartha IW & Sunari AS. 2013. Struktur komunitas parasitoid yang berasosiasi dengan *Liriomyza sativae* (Blanchard) (Diptera:Agromyzidae) pada berbagai tanaman inangdi dataran rendah. *Jurnal Agroekoteknologi Tropika* **2**, 244-251.

Hesami S, Yefremova Z, & Seyedebrahimi S. 2006. Report of *Cirrospilus variegatus* (Hym.: Eulophidae), parasitoid of dipterous leafminers from Iran. *Journal of the Entomological Society of Iran* **26,** 93–94.

Jensen GL & Koehler CS. 1970. Seasonal and distributional abundance and parasites of leafminers of alfalfa in California. *Journal of Economic Entomology* **63**, 1623–1628.

Johnson MW. 1987. Parasitization of Liriomyza spp. (Diptera: Agromyzidae) infesting commercial watermelon plantings in Hawaii. Journal of Economic Entomology 80, 56–61.

Johnson MW. 1993. Biological control of *Liriomyza* leafminers in the Pacific basin. *Micronesica, Supplement* **4,** 81–92.

Johnson MW, Oatman ER & Wyman JA. 1980. Natural control of *Liriomyza sativae* in pole tomatoes in southern California. *Entomophaga* **25**, 193–198.

Lema K-M & Poe SL. 1979. Age specific mortality of *Liriomyza sativae* due to *Chrysonotomyia formosa* and parasitization by *Opius dimidiatus* and *Chrysonotomyia formosa*. *Environmental Entomology* **8**, 935–937.

Lotfalizadeh H & Gharali B. 2008. Pteromalidae (Hymenoptera: Chalcidoidea) of Iran: New records and a preliminary checklist. *Entomofauna* **29**, 93–120.

Marsh PM. 1979. Braconidae. *In* Krombein K, Hurd P, Smith D & Burks B *[eds.]. Catalog of Hymenoptera in America North of Mexico* Vol. 1. 1198 pp. Smithsonian Institution Press, Washington, D.C.

Mazumdar S & Bhuiya BA. 2016. Parasitoids (Hymenoptera) of leafminer flies (Diptera: Agromyzidae) from Bangladesh. *Journal of Threatened Taxa* **8**, 8714–8718.

McClanahan RJ. 1975. Notes on the vegetable leafminer, *Liriomyza sativae* (Diptera: Agromyzidae), in Ontario. *Proceedings of the Entomological Society of Ontario* **105**, 40–44.

McClanahan RJ. 1977. Biological control of the leafminer *Liriomyza sativae* in greenhouse crops. pp 45–48. *In* F.F. Smith & FF Webb RE *[eds]. Pest Management in Protected Culture Crops*. USDA ARS-NE-85.

Mujica N & Kroschel J. 2011. Leafminer fly (Diptera: Agromyzidae) occurrence, distribution and parasitoid associations in field and vegetable crops along the Peruvian coast. *Environmental Entomology* **40**, 217–230.

Nagalingam T, Wijayagunasekara HNP, Hemachandra KS & Nugaliyadde L. 2007. Parasitoids of *Liriomyza sativae* Blanchard (Diptera: Agromyzidae) in the mid country of Sri Lanka. *Tropical Agricultural Research* **19**, 59–68.

Noyes JS. 1994. The reliability of published host-parasitoid records: a taxonomist's view. *Norwegian Journal of Agricultural Sciences Supplement* **16**, 59–69.

Oatman ER. 1959. Natural control studies of the melon leaf miner, *Liriomyza pictella* (Thomson). *Journal of Economic Entomology* **52**, 895–898.

Oatman ER & Johnson MW. 1981. Leafminer parasite interactions, pp. 99–105. *In* D.J. Schuster [ed.], *Proceedings Institute Food and Agricultural Science-Industry Conference Biology and Control of* Liriomyza *Leafminers*, 3–4 November 1981, Lake Buena Vista, Florida.

Palumbo JC, Mullis CH, Jr & Reyes FJ. 1994. Composition, seasonal abundance, and parasitism of *Liriomyza* (Diptera: Agromyzidae) species on lettuce in Arizona. *Journal of Economic Entomology* **87**, 1070–1077.

Perry RK & Heraty JM. 2019. A tale of two setae: how morphology and ITS2 help delimit a cryptic species complex in Eulophidae (Hymenoptera: Chalcidoidea). *Insect Systematics and Diversity* **3**, 10; 1–1 doi: 10.1093/isd/ixz021

Rauf A, Shepard BM & Johnson MW. 2000. Leafminers in vegetables, ornamental plants and weeds in Indonesia: surveys of host crops, species composition and parasitoids. *International Journal of Pest Management* **46**, 257–266.

Ridland PM, Umina PA, Pirtle EI & Hoffmann AA (2020). Potential for biological control of the vegetable leafminer, *Liriomyza sativae* (Diptera: Agromyzidae), in Australia with parasitoid wasps. *Austral Entomology* **59**, 16-36.

Rodríguez-Castañeda G, MacVean C, Cardona C & Hof AR. 2017. What limits the distribution of *Liriomyza huidobrensis* and its congener *Liriomyza sativae* in their native niche: when temperature and competition affect species' distribution range in Guatemala. *Journal of Insect Science* **17(4),** 88: 1–13.

Shahreki Z, Rakhshani E & Gumovsky A. 2016. Identification parasitoids of leafminer flies in Sistan region – Iran. *International Archives of Applied Science and Technology* **7(2)**, 9–13.

Shaw MR. 1994. Parasitoid host ranges. In: *Parasitoid community ecology.* (eds BA Hawkins & Sheehan W). pp 451–471. Oxford University Press, Oxford, UK.

Sheng J-K & Zhan G-X. 2000. A new species of *Chrysonotomyia* Ashmead (Hymenoptera: Eulophidae) from China. *In* Y-L Zhang *[ed.] Systematic and faunistic research on Chinese insects. Proceedings of the 5th National Congress on Insect Taxonomy*. pp.258–259 China Agriculture Press.

Shi B, Zhang Z, Lu H, Gong Y & Wang J. 2000. Study on *Lirimyza* [sic] sativae. (Abstract 2822) Abstracts, XXI International Congress of Entomology, Brazil, August 20–26, 2000, 2: 712. http://www.nhm.ac.uk/resources/research-curation/projects/chalcidoids/pdf_X/ShiZhLu2000.pdf

Sivapragasam, A., Syed, AR, LaSalle J, Ruwaida, M. 1999. Parasitoids of invasive agromyzid leafminers on vegetables in peninsular Malaysia, pp 8–19. *In* Proceedings of symposium on Biological Control in the Tropics, 18–19 March. MARDI Training Centre, Malaysia.

Stegmaier CE Jr. 1972. Parasitic Hymenoptera bred from the family Agromyzidae (Diptera) with special reference to South Florida. *The Florida Entomologist* **55**, 273–282.

Talebi AA, Khoramabadi AM & Rakhshani E. 2011. Checklist of eulophid wasps (Insecta: Hymenoptera: Eulophidae) of Iran. *Check List* **7**, 708–719.

Tantowijoyo W & Hoffmann AA. 2010. Identifying factors determining the altitudinal distribution of the invasive pest leafminers *Liriomyza huidobrensis* and *Liriomyza sativae*. *Entomologia Experimentalis Applicata* **135**, 141–153.

Tran DH. 2009. Agromyzid leafminers and their parasitoids on vegetables in central Vietnam. *Journal of ISSAAS* **15(2)**, 21–33.

Tran TTA, Tran DH, Konishi K & Takagi M. 2005. The vegetable leafminer *Liriomyza sativae* Blanchard (Diptera: Agromyzidae) and its parasitoids on cucumber in the Ho Chi Minh Region of Vietnam. *Journal of the Faculty of Agriculture, Kyushu University* **50**, 119–124.

Trumble JT & Nakakihara H. 1983. Occurrence, parasitism and sampling of *Liriomyza* species (Diptera: Agromyzidae) infesting celery in California. *Environmental Entomology* **12**, 810–814.

Tryon EH & Poe SL. 1981. Development rates and emergence of vegetable leafminer pupae and their parasites reared from celery foliage. *The Florida Entomologist* **64**, 477–483.

Valladares GR, Salvo A & Videla M. 1999. Moscas minadoras en cultivos de Argentina [Leafmining flies on crops in Argentina]. *Horticultura Argentina* **18**, 56–61.

Wahyuni S, Supartha IW, Ubaidillah R & Wijaya IN (2017). Parasitoid community structure of leaf miner *Liriomyza* spp. (Diptera: Agromyzidae) and the rate of parasitization on vegetable crops in Lesser Sunda Islands, Indonesia. *Biodiversitas* **18**, 593–600.

Xing Z-L, Zhang L-Y, Wu S-Y, Yi H, Gao Y & Lei Z-G. 2017. Niche comparison among two invasive leafminer species and their parasitoid Opius biroi: implications for competitive displacement. Scientific Reports 7, 4246 https://doi.org/10.1038/s41598–017–04562–3

Xu Z, Gao Z, Chen X, Hou R and Zeng L. 1999. Hymenopterous parasitoids of *Liriomyza sativae* Blanchard (Diptera: Agromyzidae) in Guangdong province, China. *Natural Enemies of Insects* **21**, 126–131 (in Chinese).

Ye F-Y, Zhu C-D, Yefremova Z, Liu W-X, Guo J-Y & Wan F-H. 2018. Life history and biocontrol potential of the first female-producing parthenogenetic species of *Diglyphus* (Hymenoptera: Eulophidae) against agromyzid leafminers. *Scientific Reports* **8**, 3222 https://doi.org/10.1038/s41598–018–20972–3

Yefremova Z, Civelek HS, Boyadzhiyev P, Dursun O & Eskin A. 2011. A review of Turkish *Diglyphus* Walker (Hymenoptera: Eulophidae), with description of a new species. *Annales de la Société Entomologique de France (N.S.)* **47**, 273–279.

Table S4 Overlap of parasitoid species recorded from the three *Liriomyza* spp.

(a) parasitoid species recorded from all three *Liriomyza* spp.

Family	Sub-Family	Species	*Liriomyza huidobrensis*	*Liriomyza trifolii*	*Liriomyza sativae*
Braconidae	Opiinae	*Opius dimidiatus*	1	1	1
Braconidae	Opiinae	*Opius dissitus*	1	1	1
Eulophidae	Entedoninae	*Asecodes delucchii*	1	1	1
Eulophidae	Entedoninae	*Chrysocharis ainsliei*	1	1	1
Eulophidae	Entedoninae	*Chrysocharis caribea*	1	1	1
Eulophidae	Entedoninae	*Chrysocharis oscinidis*	1	1	1
Eulophidae	Entedoninae	*Chrysocharis pentheus*	1	1	1
Eulophidae	Entedoninae	*Closterocerus cinctipennis*	1	1	1
Eulophidae	Entedoninae	*Neochrysocharis diastatae*	1	1	1
Eulophidae	Entedoninae	*Neochrysocharis formosa*	1	1	1
Eulophidae	Entedoninae	*Neochrysocharis okazakii*	1	1	1
Eulophidae	Eulophinae	*Burkseus vittatus*	1	1	1
Eulophidae	Eulophinae	*Cirrospilus brevicorpus*	1	1	1
Eulophidae	Eulophinae	*Diaulinopsis callichroma*	1	1	1
Eulophidae	Eulophinae	*Diglyphus albiscapus*	1	1	1
Eulophidae	Eulophinae	*Diglyphus begini*	1	1	1
Eulophidae	Eulophinae	*Diglyphus crassinervis*	1	1	1
Eulophidae	Eulophinae	*Diglyphus intermedius*	1	1	1
Eulophidae	Eulophinae	*Diglyphus isaea*	1	1	1
Eulophidae	Eulophinae	*Diglyphus minoeus*	1	1	1
Eulophidae	Eulophinae	*Diglyphus pulchripes*	1	1	1
Eulophidae	Eulophinae	*Hemiptarsenus varicornis*	1	1	1
Eulophidae	Eulophinae	*Hemiptarsenus zilahisebessi*	1	1	1
Figitidae	Eucoilinae	*Gronotoma micromorpha*	1	1	1
Figitidae	Eucoilinae	*Sinatra pacifica*	1	1	1
Pteromalidae	Miscogastrinae	*Halticoptera circulus*	1	1	1
Pteromalidae	Miscogastrinae	*Halticoptera patellana*	1	1	1
∑			**27**	**27**	**27**

(b) parasitoid species recorded from *L. huidobrensis* and *L. trifolii*, but not *L. sativae*

Table S4 Overlap of parasitoid species recorded from the three *Liriomyza* spp.

Family	Sub-Family	*Species*	*Liriomyza huidobrensis*	*Liriomyza trifolii*	*Liriomyza sativae*
Braconidae	Alysiinae	*Dacnusa sasakawai*	1	1	
Braconidae	Opiinae	*Opius pallipes*	1	1	
Eulophidae	Entedoninae	*Chrysocharis orbicularis*	1	1	
Eulophidae	Entedoninae	*Chrysocharis pubicornis*	1	1	
Eulophidae	Entedoninae	*Pediobius metallicus*	1	1	
Eulophidae	Eulophinae	*Diglyphus pachyneurus*	1	1	
Eulophidae	Eulophinae	*Hemiptarsenus ornatus*	1	1	
Eulophidae	Eulophinae	*Pnigalio incompletus*	1	1	
Eulophidae	Eulophinae	*Pnigalio katonis*	1	1	
Eulophidae	Eulophinae	*Pnigalio soemius*	1	1	
Eulophidae	Tetrastichinae	*Quadrastichus liriomyzae*	1	1	
∑			**11**	**11**	

(c) parasitoid species recorded from *L. huidobrensis* and *L. sativae*, but not *L. trifolii*

Family	Sub-Family	*Species*	*Liriomyza huidobrensis*	*Liriomyza trifolii*	*Liriomyza sativae*
Braconidae	Opiinae	*Opius caricivorae*	1		1
Braconidae	Opiinae	*Opius chromatomyiae*	1		1
Braconidae	Opiinae	*Opius (Phaedrotoma) scabriventris*	1		1
Eulophidae	Entedoninae	*Chrysocharis flacilla*	1		1
Eulophidae	Entedoninae	*Chrysocharis vonones*	1		1
Eulophidae	Entedoninae	*Neochrysocharis beasleyi*	1		1
Eulophidae	Entedoninae	*Proacrias thysanoides*	1		1
Eulophidae	Eulophinae	*Diglyphus wani*	1		1
Eulophidae	Eulophinae	*Diglyphus websteri*	1		1
Eulophidae	Eulophinae	*Zagrammosoma multilineatum*	1		1
Figitidae	Eucoilinae	*Gronotoma adachiae*	1		1
Pteromalidae	Miscogastrinae	*Halticoptera arduine*	1		1
∑			**12**		**12**

Table S4 Overlap of parasitoid species recorded from the three *Liriomyza* spp.

(d) parasitoid species recorded from *L. trifolii* and *L. sativae*, but not *L. huidobrensis*

**Family**	**Sub-Family**	***Species***	***Liriomyza huidobrensis***	***Liriomyza trifolii***	***Liriomyza sativae***
Braconidae	Opiinae	*Opius bruneipes*		1	1
Braconidae	Opiinae	*Opius (Phaedrotoma) biroi*		1	1
Eulophidae	Entedoninae	*Asecodes erxias*		1	1
Eulophidae	Entedoninae	*Closterocerus purpureus*		1	1
Eulophidae	Entedoninae	*Closterocerus utahensis*		1	1
Eulophidae	Entedoninae	*Neochrysocharis agromyzae*		1	1
Eulophidae	Eulophinae	*Diaulinopsis arenaria*		1	1
Eulophidae	Eulophinae	*Diglyphus chabrias*		1	1
Eulophidae	Eulophinae	*Diglyphus pusztensis*		1	1
Eulophidae	Eulophinae	*Meruacesa liriomyzae*		1	1
Eulophidae	Eulophinae	*Zagrammosoma americanum*		1	1
Eulophidae	Eulophinae	*Zagrammosoma variegatum*		1	1
Figitidae	Eucoilinae	*Banacuniculus utilis*		1	1
Figitidae	Eucoilinae	*Ganaspidium pusillae*		1	1
Pteromalidae	Miscogastrinae	*Halticoptera aenea*		1	1
Pteromalidae	Pteromalinae	*Cyrtogaster vulgaris*		1	1
∑				**16**	**16**

(e) parasitoid species recorded only from *L. huidobrensis*

**Family**	**Sub-Family**	***Species***	***Liriomyza huidobrensis***	***Liriomyza trifolii***	***Liriomyza sativae***
Braconidae	Alysiinae	*Dacnusa sibirica*	1		
Braconidae	Braconinae	*Bracon intercessor*	1		
Braconidae	Opiinae	*Opius mandibularis*	1		
Braconidae	Opiinae	*Opius meracus*	1		
Braconidae	Opiinae	*Opius (Phaedrotoma) luteoclypealis*	1		
Braconidae	Opiinae	*Opius (Phaedrotoma) mesoclypealis*	1		
Eulophidae	Entedoninae	*Chrysocharis bedius*	1		
Eulophidae	Entedoninae	*Chrysocharis ignota*	1		
Eulophidae	Entedoninae	*Chrysocharis tristis*	1		
Eulophidae	Entedoninae	*Closterocerus pulcher*	1		

Table S4 Overlap of parasitoid species recorded from the three *Liriomyza* spp.

**Family**	**Sub-Family**	***Species***	***Liriomyza huidobrensis***	***Liriomyza trifolii***	***Liriomyza sativae***
Eulophidae	Entedoninae	*Proacrias xenodice*	1		
Eulophidae	Eulophinae	*Diaulinopsis* sp.	1		
Eulophidae	Eulophinae	*Diglyphus bimaculatus*	1		
Eulophidae	Eulophinae	*Diglyphus pedicellus*	1		
Eulophidae	Eulophinae	*Diglyphus poppoea*	1		
Eulophidae	Eulophinae	*Hemiptarsenus fulvicollis*	1		
Eulophidae	Eulophinae	*Hemiptarsenus unguicellus*	1		
Eulophidae	Eulophinae	*Zagrammosoma latilineatum*	1		
Figitidae	Eucoilinae	*Zaeucoila clavatus*	1		
Pteromalidae	Miscogastrinae	*Halticoptera helioponi*	1		
Pteromalidae	Miscogastrinae	*Halticoptera peruviana*	1		
Pteromalidae	Miscogastrinae	*Thinodytes cyzicus*	1		
Pteromalidae	Pteromalinae	*Notoglyptus tzeltales*	1		
Tetracampidae	Platynocheilinae	*Platynocheilus cuprifrons*	1		
∑			**24**		

(f) parasitoid species recorded only from *L. trifolii*

**Family**	**Sub-Family**	***Species***	***Liriomyza huidobrensis***	***Liriomyza trifolii***	***Liriomyza sativae***
Braconidae	Alysiinae	*Dacnusa nipponica*		1	
Braconidae	Alysiinae	*Pseudopezomachus masii*		1	
Braconidae	Opiinae	*Opius ambiguus*		1	
Braconidae	Opiinae	*Opius basalis*		1	
Braconidae	Opiinae	*Opius exigua*		1	
Braconidae	Opiinae	*Opius gafsaensis*		1	
Braconidae	Opiinae	*Opius osogovoensis*		1	
Eulophidae	Entedoninae	*Apleurotropis kumatai*		1	
Eulophidae	Entedoninae	*Chrysocharis liriomyzae*		1	
Eulophidae	Entedoninae	*Chrysonotomyia rexia*		1	
Eulophidae	Entedoninae	*Chrysonotomyia ricini*		1	
Eulophidae	Entedoninae	*Chrysonotomyia smaragdulus*		1	
Eulophidae	Entedoninae	*Closterocerus agromyzae*		1	

Table S4 Overlap of parasitoid species recorded from the three *Liriomyza* spp.

**Family**	**Sub-Family**	***Species***	***Liriomyza huidobrensis***	***Liriomyza trifolii***	***Liriomyza sativae***
Eulophidae	Entedoninae	*Neochrysocharis ambitiosa*		1	
Eulophidae	Entedoninae	*Neochrysocharis arvensis*		1	
Eulophidae	Entedoninae	*Neochrysocharis chlorogaster*		1	
Eulophidae	Entedoninae	*Neochrysocharis indicus*		1	
Eulophidae	Entedoninae	*Neochrysocharis pictipes*		1	
Eulophidae	Entedoninae	*Neochrysocharis sericea*		1	
Eulophidae	Eulophinae	*Diglyphus horticola*		1	
Eulophidae	Eulophinae	*Pnigalio cristatus*		1	
Eulophidae	Eulophinae	*Sympiesis acalle*		1	
Eulophidae	Eulophinae	*Sympiesis gordius*		1	
Eulophidae	Eulophinae	*Zagrammosoma lineaticeps*		1	
Eulophidae	Eulophinae	*Zagrammosoma talitzkii*		1	
Eulophidae	Tetrastichinae	*Baryscapus impeditus*		1	
Eulophidae	Tetrastichinae	*Neotrichoporoides szelenyii*		1	
Eulophidae	Tetrastichinae	*Quadrastichus plaquoi*		1	
Figitidae	Eucoilinae	*Banacuniculus nigrimanus*		1	
Figitidae	Eucoilinae	*Gronotoma fetura*		1	
Figitidae	Eucoilinae	*Gronotoma guamensis*		1	
Figitidae	Eucoilinae	*Nordlanderia plowa*		1	
Figitidae	Eucoilinae	*Zaeucoila robusta*		1	
Pteromalidae	Miscogastrinae	*Halticoptera longipetiolus*		1	
Pteromalidae	Pteromalinae	*Sphegigaster brevicornis*		1	
Pteromalidae	Pteromalinae	*Sphegigaster hamugurivora*		1	
Pteromalidae	Pteromalinae	*Trichomalopsis oryzae*		1	
Tetracampidae	Tetracampinae	*Epiclerus nomocerus*		1	
Σ				**38**	

Table S4 Overlap of parasitoid species recorded from the three *Liriomyza* spp.

(g) parasitoid species recorded only from *L. sativae*

**Family**	**Sub-Family**	***Species***	***Liriomyza huidobrensis***	***Liriomyza trifolii***	***Liriomyza sativae***
Braconidae	Opiinae	*Desmiostoma parvulum*			1
Braconidae	Opiinae	*Oenongastra microrhopalae*			1
Braconidae	Opiinae	*Opius aridis*			1
Braconidae	Opiinae	*Opius liriomyzae*			1
Braconidae	Opiinae	*Opius (Phaedrotoma) suturalis*			1
Eulophidae	Entedoninae	*Achrysocharoides zwoelferi*			1
Eulophidae	Entedoninae	*Chrysocharis crassiscapus*			1
Eulophidae	Entedoninae	*Chrysocharis giraulti*			1
Eulophidae	Entedoninae	*Chrysonotomyia reticulata*			1
Eulophidae	Entedoninae	*Closterocerus cincinnatus*			1
Eulophidae	Entedoninae	*Closterocerus trifasciatus*			1
Eulophidae	Entedoninae	*Neochrysocharis arizonensis*			1
Eulophidae	Eulophinae	*Burkseus flavoviridis*			1
Eulophidae	Eulophinae	*Diglyphus sensilis*			1
Eulophidae	Eulophinae	*Hemiptarsenus waiIeseIIae*			1
Eulophidae	Eulophinae	*Pnigalio minio*			1
Eulophidae	Eulophinae	*Zagrammosoma mirum*			1
∑					**17**

Table S5 Parasitoid species by location records in descending order (pooling all records for a particular location for each *Liriomyza* species)

**Family**	**Sub-Family**	***Species***	***Liriomyza huidobrensis***	***Liriomyza trifolii***	***Liriomyza sativae***	**∑**
Eulophidae	Eulophinae	*Diglyphus isaea*	15	12	5	32
Eulophidae	Entedoninae	*Neochrysocharis formosa*	7	16	7	30
Eulophidae	Eulophinae	*Hemiptarsenus varicornis*	6	11	9	26
Braconidae	Opiinae	*Opius* sp.	13	6	1	20
Eulophidae	Entedoninae	*Chrysocharis pentheus*	5	5	5	15
Eulophidae	Eulophinae	*Diglyphus crassinervis*	5	6	2	13
Braconidae	Opiinae	*Opius dissitus*	4	5	3	12
Pteromalidae	Miscogastrinae	*Halticoptera circulus*	5	5	2	12
Eulophidae	Entedoninae	*Neochrysocharis okazakii*	3	5	3	11
Eulophidae	Entedoninae	*Chrysocharis* sp.	6	4		10
Braconidae	Opiinae	*Opius dimidiatus*	2	4	3	9
Eulophidae	Eulophinae	*Hemiptarsenus zilahisebessi*	3	5	1	9
Eulophidae	Eulophinae	*Diglyphus begini*	4	2	3	9
Eulophidae	Entedoninae	*Chrysocharis caribea*	2	2	4	8
Eulophidae	Entedoninae	*Chrysocharis oscinidis*	1	4	3	8
Eulophidae	Eulophinae	*Diglyphus* sp.	6	2		8
Pteromalidae	Miscogastrinae	*Halticoptera* sp.	7	1		8
Eulophidae	Entedoninae	*Asecodes delucchii*	2	2	3	7
Eulophidae	Eulophinae	*Diglyphus intermedius*	4	2	1	7
Eulophidae	Eulophinae	*Burkseus vittatus*	2	4	1	7
Braconidae	Alysiinae	*Dacnusa sibirica*	6			6
Eulophidae	Eulophinae	*Diglyphus minoeus*	3	2	1	6
Eulophidae	Eulophinae	*Diglyphus websteri*	3		3	6
Eulophidae	Entedoninae	*Neochrysocharis diastatae*	1	2	2	5
Eulophidae	Entedoninae	*Closterocerus cinctipennis*	1	3	1	5
Eulophidae	Entedoninae	*Neochrysocharis* sp.	1	4		5

Table S5 Parasitoid species by location records in descending order (pooling all records for a particular location for each *Liriomyza* species)

**Family**	**Sub-Family**	***Species***	***Liriomyza huidobrensis***	***Liriomyza trifolii***	***Liriomyza sativae***	**∑**
Eulophidae	Eulophinae	*Diaulinopsis callichroma*	1	2	2	5
Figitidae	Eucoilinae	*Banacuniculus utilis*		4	1	5
Figitidae	Eucoilinae	*Sinatra pacifica*	1	3	1	5
Braconidae	Opiinae	*Opius (Phaedrotoma) scabriventris*	3		1	4
Braconidae	Opiinae	*Opius pallipes*	2	2		4
Eulophidae	Entedoninae	*Pediobius metallicus*	3	1		4
Eulophidae	Entedoninae	*Chrysocharis pubicornis*	3	1		4
Eulophidae	Entedoninae	*Chrysonotomyia* sp.	2	2		4
Eulophidae	Entedoninae	*Chrysocharis vonones*	2		2	4
Eulophidae	Entedoninae	*Chrysocharis flacilla*	3		1	4
Eulophidae	Eulophinae	*Pnigalio* sp.	2	2		4
Eulophidae	Eulophinae	*Pnigalio katonis*	2	2		4
Eulophidae	Eulophinae	*Diglyphus pulchripes*	1	2	1	4
Eulophidae	Eulophinae	*Zagrammosoma* sp.	2	2		4
Eulophidae	Eulophinae	*Cirrospilus brevicorpus*	1	1	2	4
Eulophidae	Eulophinae	*Diglyphus albiscapus*	1	2	1	4
Eulophidae	Tetrastichinae	*Quadrastichus liriomyzae*	1	3		4
Eulophidae	Tetrastichinae	*Quadrastichus* sp.	3	1		4
Figitidae	Eucoilinae	*Gronotoma micromorpha*	1	2	1	4
Pteromalidae	Miscogastrinae	*Halticoptera patellana*	1	1	2	4
Braconidae	Opiinae	*Opius caricivorae*	1		2	3
Braconidae	Opiinae	*Opius chromatomyiae*	1		2	3
Eulophidae	Entedoninae	*Asecodes erxias*		2	1	3
Eulophidae	Entedoninae	*Chrysocharis bedius*	3			3
Eulophidae	Entedoninae	*Closterocerus* sp.	3			3
Eulophidae	Entedoninae	*Chrysocharis ainsliei*	1	1	1	3

Table S5 Parasitoid species by location records in descending order (pooling all records for a particular location for each *Liriomyza* species)

**Family**	**Sub-Family**	***Species***	***Liriomyza huidobrensis***	***Liriomyza trifolii***	***Liriomyza sativae***	**∑**
Eulophidae	Entedoninae	*Proacrias thysanoides*	2		1	3
Eulophidae	Entedoninae	*Neochrysocharis beasleyi*	2		1	3
Eulophidae	Eulophinae	*Hemiptarsenus* sp.	2	1		3
Eulophidae	Eulophinae	*Zagrammosoma americanum*		1	2	3
Eulophidae	Eulophinae	*Meruacesa liriomyzae*		1	2	3
Eulophidae	Eulophinae	*Diglyphus chabrias*		2	1	3
Eulophidae	Eulophinae	*Hemiptarsenus ornatus*	1	2		3
Eulophidae	Eulophinae	*Diaulinopsis arenaria*		2	1	3
Figitidae	Eucoilinae	*Gronotoma* sp.	3			3
Pteromalidae	Miscogastrinae	*Halticoptera arduine*	2		1	3
Pteromalidae	unidentified	Pteromalidae species	3			3
Braconidae	Alysiinae	*Dacnusa* sp.	2			2
Braconidae	Alysiinae	*Dacnusa sasakawai*	1	1		2
Braconidae	Opiinae	*Opius (Phaedrotoma) biroi*		1	1	2
Braconidae	Opiinae	*Opius bruneipes*		1	1	2
Braconidae	Opiinae	*Opius basalis*		2		2
Braconidae	Opiinae	*Opius exigua*		2		2
Eulophidae	Entedoninae	*Closterocerus utahensis*		1	1	2
Eulophidae	Entedoninae	*Neochrysocharis agromyzae*		1	1	2
Eulophidae	Entedoninae	*Proacrias xenodice*	2			2
Eulophidae	Entedoninae	*Asecodes* sp.	1	1		2
Eulophidae	Entedoninae	*Chrysocharis orbicularis*	1	1		2
Eulophidae	Entedoninae	*Closterocerus purpureus*		1	1	2
Eulophidae	Eulophinae	*Pnigalio soemius*	1	1		2
Eulophidae	Eulophinae	*Zagrammosoma multilineatum*	1		1	2
Eulophidae	Eulophinae	*Diaulinopsis* sp.	2			2

Table S5 Parasitoid species by location records in descending order (pooling all records for a particular location for each *Liriomyza* species)

**Family**	**Sub-Family**	***Species***	***Liriomyza huidobrensis***	***Liriomyza trifolii***	***Liriomyza sativae***	**∑**
Eulophidae	Eulophinae	*Diglyphus pachyneurus*	1	1		2
Eulophidae	Eulophinae	*Pnigalio incompletus*	1	1		2
Eulophidae	Eulophinae	*Diglyphus wani*	1		1	2
Eulophidae	Eulophinae	*Diglyphus pusztensis*		1	1	2
Eulophidae	Eulophinae	*Zagrammosoma variegatum*		1	1	2
Eulophidae	Eulophinae	*Cirrospilus* sp. near *cinctiventris*		2		2
Figitidae	Eucoilinae	*Banacuniculus nigrimanus*		2		2
Figitidae	Eucoilinae	*Ganaspidium pusillae*		1	1	2
Figitidae	Eucoilinae	*Gronotoma adachiae*	1		1	2
Figitidae	Eucoilinae	*Ganaspidium* sp.	1	1		2
Pteromalidae	Miscogastrinae	*Halticoptera aenea*		1	1	2
Pteromalidae	Pteromalinae	*Sphegigaster* sp.	1	1		2
Pteromalidae	Pteromalinae	*Cyrtogaster vulgaris*		1	1	2
Braconidae	Alysiinae	*Dacnusa nipponica*		1		1
Braconidae	Alysiinae	*Pseudopezomachus masii*		1		1
Braconidae	Alysiinae	*Oenonogastra* sp.	1			1
Braconidae	Braconinae	*Bracon intercessor*	1			1
Braconidae	Opiinae	*Oenongastra microrhopalae*			1	1
Braconidae	Opiinae	*Opius (Phaedrotoma) luteoclypealis*	1			1
Braconidae	Opiinae	*Opius* sp. 2		1		1
Braconidae	Opiinae	*Opius ambiguus*		1		1
Braconidae	Opiinae	*Opius aridis*			1	1
Braconidae	Opiinae	*Opius (Phaedrotoma) suturalis*			1	1
Braconidae	Opiinae	*Opius* sp. 1		1		1
Braconidae	Opiinae	*Opius meracus*	1			1
Braconidae	Opiinae	*Opius thoracosema* sp. *3*		1		1

Table S5 Parasitoid species by location records in descending order (pooling all records for a particular location for each *Liriomyza* species)

**Family**	**Sub-Family**	***Species***	***Liriomyza huidobrensis***	***Liriomyza trifolii***	***Liriomyza sativae***	**∑**
Braconidae	Opiinae	*Opius* near *brownsvillensis*		1		1
Braconidae	Opiinae	*Opius osogovoensis*		1		1
Braconidae	Opiinae	*Opius (Phaedrotoma) mesoclypealis*	1			1
Braconidae	Opiinae	*Opius gafsaensis*		1		1
Braconidae	Opiinae	*Opius (Phaedrotoma)* sp.	1			1
Braconidae	Opiinae	*Opius liriomyzae*			1	1
Braconidae	Opiinae	*Desmiostoma parvulum*			1	1
Braconidae	Opiinae	*Opius mandibularis*	1			1
Braconidae	Tersilochinae	*Allophrys* sp.		1		1
Diapriidae	Diapriinae	*Trichopria* sp.	1			1
Eulophidae	Entedoninae	*Neochrysocharis indicus*		1		1
Eulophidae	Entedoninae	*Chrysonotomyia smaragdulus*		1		1
Eulophidae	Entedoninae	*Closterocerus agromyzae*		1		1
Eulophidae	Entedoninae	*Chrysocharis tristis*	1			1
Eulophidae	Entedoninae	*Pediobius* sp.		1		1
Eulophidae	Entedoninae	*Chrysocharis* near *aluta*	1			1
Eulophidae	Entedoninae	*Apleurotropis kumatai*		1		1
Eulophidae	Entedoninae	*Closterocerus pulcher*	1			1
Eulophidae	Entedoninae	*Neochrysocharis pictipes*		1		1
Eulophidae	Entedoninae	*Chrysocharis liriomyzae*		1		1
Eulophidae	Entedoninae	*Chrysonotomyia* sp.*F*		1		1
Eulophidae	Entedoninae	*Chrysonotomyia rexia*		1		1
Eulophidae	Entedoninae	*Neochrysocharis chlorogaster*		1		1
Eulophidae	Entedoninae	*Closterocerus trifasciatus*			1	1
Eulophidae	Entedoninae	*Chrysocharis crassiscapus*			1	1
Eulophidae	Entedoninae	*Chrysonotomyia ricini*		1		1

Table S5 Parasitoid species by location records in descending order (pooling all records for a particular location for each *Liriomyza* species)

**Family**	**Sub-Family**	***Species***	***Liriomyza huidobrensis***	***Liriomyza trifolii***	***Liriomyza sativae***	∑
Eulophidae	Entedoninae	*Chrysonotomyia reticulata*			1	1
Eulophidae	Entedoninae	*Neochrysocharis sericea*		1		1
Eulophidae	Entedoninae	*Closterocerus cincinnatus*			1	1
Eulophidae	Entedoninae	*Chrysocharis giraulti*			1	1
Eulophidae	Entedoninae	*Chrysocharis ignota*	1			1
Eulophidae	Entedoninae	*Neochrysocharis ambitiosa*		1		1
Eulophidae	Entedoninae	*Proacrias* sp.	1			1
Eulophidae	Entedoninae	*Neochrysocharis arizonensis*			1	1
Eulophidae	Entedoninae	*Neochrysocharis arvensis*		1		1
Eulophidae	Entedoninae	*Achrysocharoides zwoelferi*			1	1
Eulophidae	Entedoninae	*Chrysonotomyia* sp. near *leptocera*		1		1
Eulophidae	Eulophinae	*Sympiesis* sp.		1		1
Eulophidae	Eulophinae	*Diglyphus horticola*		1		1
Eulophidae	Eulophinae	*Zagrammosoma mirum*			1	1
Eulophidae	Eulophinae	*Burkseus flavoviridis*			1	1
Eulophidae	Eulophinae	*Sympiesis acalle*		1		1
Eulophidae	Eulophinae	*Hemiptarsenus waiIeseIIae*			1	1
Eulophidae	Eulophinae	*Zagrammosoma latilineatum*	1			1
Eulophidae	Eulophinae	*Zagrammosoma talitzkii*		1		1
Eulophidae	Eulophinae	*Diglyphus pedicellus*	1			1
Eulophidae	Eulophinae	*Diglyphus bimaculatus*	1			1
Eulophidae	Eulophinae	*Pnigalio* sp near *pectinicornis*		1		1
Eulophidae	Eulophinae	*Elachertus* sp. L		1		1
Eulophidae	Eulophinae	*Sympiesis gordius*		1		1
Eulophidae	Eulophinae	*Diglyphus sensilis*			1	1
Eulophidae	Eulophinae	*Diglyphus poppoea*	1			1

Table S5 Parasitoid species by location records in descending order (pooling all records for a particular location for each *Liriomyza* species)

**Family**	**Sub-Family**	***Species***	***Liriomyza huidobrensis***	***Liriomyza trifolii***	***Liriomyza sativae***	**∑**
Eulophidae	Eulophinae	*Hemiptarsenus fulvicollis*	1			1
Eulophidae	Eulophinae	*Zagrammosoma lineaticeps*		1		1
Eulophidae	Eulophinae	*Cirrospilus* sp.		1		1
Eulophidae	Eulophinae	*Diglyphus* sp. *(near intermedius)*	1			1
Eulophidae	Eulophinae	*Pnigalio minio*			1	1
Eulophidae	Eulophinae	*Meruacesa* sp.	1			1
Eulophidae	Eulophinae	*Pnigalio cristatus*		1		1
Eulophidae	Eulophinae	*Cirrospilus variegatus group*		1		1
Eulophidae	Eulophinae	*Hemiptarsenus unguicellus*	1			1
Eulophidae	Tetrastichinae	*Quadrastichus plaquoi*		1		1
Eulophidae	Tetrastichinae	*Baryscapus impeditus*		1		1
Eulophidae	Tetrastichinae	*Aprostocetus* sp.		1		1
Eulophidae	Tetrastichinae	*Neotrichoporoides szelenyii*		1		1
Eulophidae	Tetrastichinae	*Oomyzus* sp.		1		1
Figitidae	Eucoilinae	*Alloxysta* sp.	1			1
Figitidae	Eucoilinae	*Gronotoma guamensis*		1		1
Figitidae	Eucoilinae	*Nordlanderia plowa*		1		1
Figitidae	Eucoilinae	*Zaeucoila robusta*		1		1
Figitidae	Eucoilinae	*Tribliographa* sp.	1			1
Figitidae	Eucoilinae	*Gronotoma fetura*		1		1
Figitidae	Eucoilinae	*Moneucoela* sp.	1			1
Figitidae	Eucoilinae	*Zaeucolia* sp.	1			1
Figitidae	Eucoilinae	*Kleidotoma* sp.		1		1
Figitidae	Eucoilinae	*Zaeucoila clavatus*	1			1
Pteromalidae	Herbertiinae	*Herbertia indica*		1		1
Pteromalidae	Miscogastrinae	*Halticoptera* sp. *1*		1		1

Table S5 Parasitoid species by location records in descending order (pooling all records for a particular location for each *Liriomyza* species)

Family	Sub-Family	*Species*	*Liriomyza huidobrensis*	*Liriomyza trifolii*	*Liriomyza sativae*	∑
Pteromalidae	Miscogastrinae	*Thinodytes cyzicus*	1			1
Pteromalidae	Miscogastrinae	*Halticoptera* sp. 2		1		1
Pteromalidae	Miscogastrinae	*Thinodytes* sp.	1			1
Pteromalidae	Miscogastrinae	*Halticoptera nr. circulus*		1		1
Pteromalidae	Miscogastrinae	*Halticoptera longipetiolus*		1		1
Pteromalidae	Miscogastrinae	*Halticoptera peruviana*	1			1
Pteromalidae	Miscogastrinae	*Halticoptera helioponi*	1			1
Pteromalidae	Pteromalinae	*Heteroschema* sp.	1			1
Pteromalidae	Pteromalinae	*Notoglyptus tzeltales*	1			1
Pteromalidae	Pteromalinae	*Trichomalopsis oryzae*		1		1
Pteromalidae	Pteromalinae	*Trichomalopsis* sp.	1			1
Pteromalidae	Pteromalinae	*Sphegigaster hamugurivora*		1		1
Pteromalidae	Pteromalinae	*Sphegigaster brevicornis*		1		1
Tetracampidae	Platynocheilinae	*Platynocheilus cuprifrons*	1			1
Tetracampidae	Tetracampinae	*Epiclerus* sp.	1			1
Tetracampidae	Tetracampinae	*Epiclerus nomocerus*		1		1
		**Grand Total**	228	240	123	591
